# The transcription factor C/EBPβ promotes hyperglycemia-elicited glycolysis and liver cancer progression

**DOI:** 10.1038/s41467-026-76209-9

**Published:** 2026-08-01

**Authors:** Yifan Luo, Zhengjiang Qian, Guandou Yuan, Shuai Yang, Wei Gong, Yangyang Zhai, Wenting Dai, Jiawei An, Yuchu Liu, Qiuyue Jiang, Yang Su, Ning Ren, Min Guan, Yongfeng Yang, Feng Rao, Kunyan He, Keqiang Ye

**Affiliations:** 1 Brain Cognition and Brain Disease Institute (BCBDI), Shenzhen Institutes of Advanced Technology (SIAT), Chinese Academy of Sciences, Shenzhen Guangdong, China; 2 Faculty of Life and Health Sciences, Shenzhen University of Advanced Technology (SUAT), University of Chinese Academy of Science, Shenzhen, Guangdong, China; 3 Division of Hepatobiliary Surgery, The First Affiliated Hospital of Guangxi Medical University, Nanning, China; 4 School of Life Sciences, Westlake University, Hangzhou, Zhejiang China; 5 Department of General Surgery, Shanghai Research Center of Biliary Tract Disease, Shanghai Key Laboratory of Biliary Tract Disease Research, Xinhua Hospital Affiliated to Shanghai Jiao Tong University School of Medicine, Shanghai, China; 6 School of Biological Sciences, University of Hong Kong, Hong Kong SAR, China; 7 School of Biology and Biological Engineering, South China University of Technology, Guangzhou, China; 8 School of Life Sciences, Southern University of Science and Technology, Shenzhen, Guangdong, China; 9 Paul C. Lauterbur Research Center for Biomedical Imaging, Shenzhen Institutes of Advanced Technology, Chinese Academy of Sciences, Shenzhen, China; 10 Research Center for Human Tissues and Organs Degeneration, Institute of Biomedicine and Biotechnology, Shenzhen Institutes of Advanced Technology, Chinese Academy of Sciences, Shenzhen Guangdong, China; 11 Key Laboratory of Systems Biomedicine (Ministry of Education) and Shanghai Center for Systems Biomedicine, Shanghai Jiao Tong University, Shanghai, China

**Keywords:** Cancer metabolism, Transcriptional regulatory elements, Oncogenes

## Abstract

Hyperglycemia and its related diseases, such as diabetes, dramatically accelerate cancer progression. However, the underlying mechanisms remain poorly understood, and the development of anticancer therapies based on them has largely stalled. Transcription factor CCAAT/enhancer binding protein beta (C/EBPβ) is strongly associated with glucose metabolic disorders and cancer progression. Here we show that C/EBPβ is significantly upregulated in hepatocellular carcinoma patients previously diagnosed with diabetes. Notably, high glucose activates C/EBPβ transcription via ROS-dependent PERK-eIF2α-ATF4 signaling. Intriguingly, the LAP isoforms of C/EBPβ, but not the LIP isoform, upregulate key glycolytic effectors (GLUT1 and LDHA) and the oncoprotein HRAS, thereby promoting glycolysis and proliferation in liver cancer cells. Remarkably, hyperglycemia-accelerated hepatocellular glycometabolism and hepatocellular carcinoma progression in male mice are greatly prevented by hepatocyte-specific C/EBPβ deletion or Lucicebtide (ST101) treatment, a clinical phase-II C/EBPβ inhibitory peptide. Moreover, human C/EBPβ isoform LAP1 mimics the effect of hyperglycemia to boost hepatocellular glycometabolism and accelerate hepatocellular carcinoma progression. Thus, C/EBPβ is a critical regulator and potential therapeutic target for glucose-fueled cancer progression.

## Abstract (web summary)

High blood sugar in diabetes is linked to faster cancer growth, but the mechanisms remain unclear. Here, the authors show that the transcription factor C/EBPβ is activated by high glucose to enhance glycolysis and HRAS-driven proliferation, promoting liver cancer progression and revealing a potential therapeutic target.

## Introduction

Glucose is a critical driver for cancer development^1^. Epidemiological studies indicate that each 1 mM increment in fasting blood glucose elevates cancer risk by 5–15%^2,3^. Diabetes mellitus (DM), characterized by chronic hyperglycemia, exhibits a 20–25% overall elevation in cancer incidence and a 57 % rise in cancer mortality, regardless of type 1 or type 2 DM^4^. Notably, liver cancer exhibits one of the strongest associations that individuals with elevated fasting glucose ( ≥ 5.77 mmol/L) face a 50-68% increased risk, and DM patients demonstrate a significantly elevated risk (2.01-fold)^2^. Multiple pro-oncogenic mechanisms of glucose have been identified, involving DNA stability, transcriptional activity dysregulation, aberrant post-transcriptional modifications, metabolic reprogramming, and tumor microenvironment remodeling^5–9^. However, these findings remain insufficient to fully elucidate the oncogenic role of glucose, and their translation into clinical therapeutic strategies has seen limited success.

Cancer cells predominantly rely on glycolysis over oxidative phosphorylation to fuel their progression, a phenomenon widely known as the Warburg effect^10^. Consequently, the expressions of glucose transporters (e.g., GLUT1), glycolytic enzymes (e.g., HK2, LDHA), and oncoproteins (e.g., RAS) are dramatically upregulated in cancer cells, although the precise regulatory mechanisms are not fully understood^11,12^. Transcription factors are able to orchestrate this metabolic shift by directly governing the transcription of critical metabolic genes. CCAAT/enhancer binding protein beta (C/EBPβ), a member of the C/EBP transcription factor family, is strongly implicated in glycometabolic dysfunctions and cancer progression. Specifically, C/EBPβ is an established transcriptional repressor of *ins1*, and its depletion alleviates pancreatic β cell loss in a diabetic mouse model^13,14^. Indeed, C/EBPβ is upregulated across multiple cancer types and correlates with poorer patient survival^15^. C/EBPβ is also reported to promote cancer progression by inhibiting the famous tumor suppressor P53, upregulating antioxidative reductases, or facilitating metastatic behaviors^16–19^. However, the potentially critical function of C/EBPβ in mediating the glucose metabolic reprogramming, which is essential for cancer progression, remains elusive. The functional diversity of C/EBPβ is governed by alternative translation of a single mRNA, generating three isoforms: LAP1 (liver-enriched activating protein 1), LAP2, and LIP (liver-enriched inhibitory protein)^20^. The LAPs primarily function as transcriptional activators, whereas LIP, lacking a transactivation domain, acts as a dominant-negative repressor.

Our current work found that the hepatic expression of C/EBPβ is specifically elevated in hepatocellular carcinoma (HCC) patients with preexisting diabetes mellitus (DM). Importantly, the C/EBPβ-LAP isoforms drive glycolysis and proliferation by upregulating key glycolytic effectors and the oncoprotein HRAS. Hepatocyte-specific depletion of C/EBPβ in mice ameliorates hyperglycemia-elicited HCC progression by suppressing glycolysis and proliferation. Conversely, ectopic expression of human C/EBPβ-LAP1 in mouse liver recapitulates the pro-tumorigenic effects of hyperglycemia, accelerating HCC progression; this effect was abrogated by hepatocyte-specific *Hras* knockdown. Furthermore, pharmacological inhibition of this axis using Lucicebtide (ST101), a C/EBPβ-targeted peptide in phase II clinical trial, attenuates hyperglycemia-driven hepatic glycolysis and HCC progression. Remarkably, ROS-dependent PERK-eIF2α-ATF4 signaling is required for high glucose-driven C/EBPβ activation. Our study establishes that hyperglycemia-induced C/EBPβ activation transcriptionally reprograms cancer metabolism and proliferation, driving tumor progression through coordinated upregulation of key glycolytic genes and HRAS.

## Results

### Hepatic C/EBPβ expression is elevated in diabetic patients and correlates with glycolytic gene expression

**Fig. 1:**
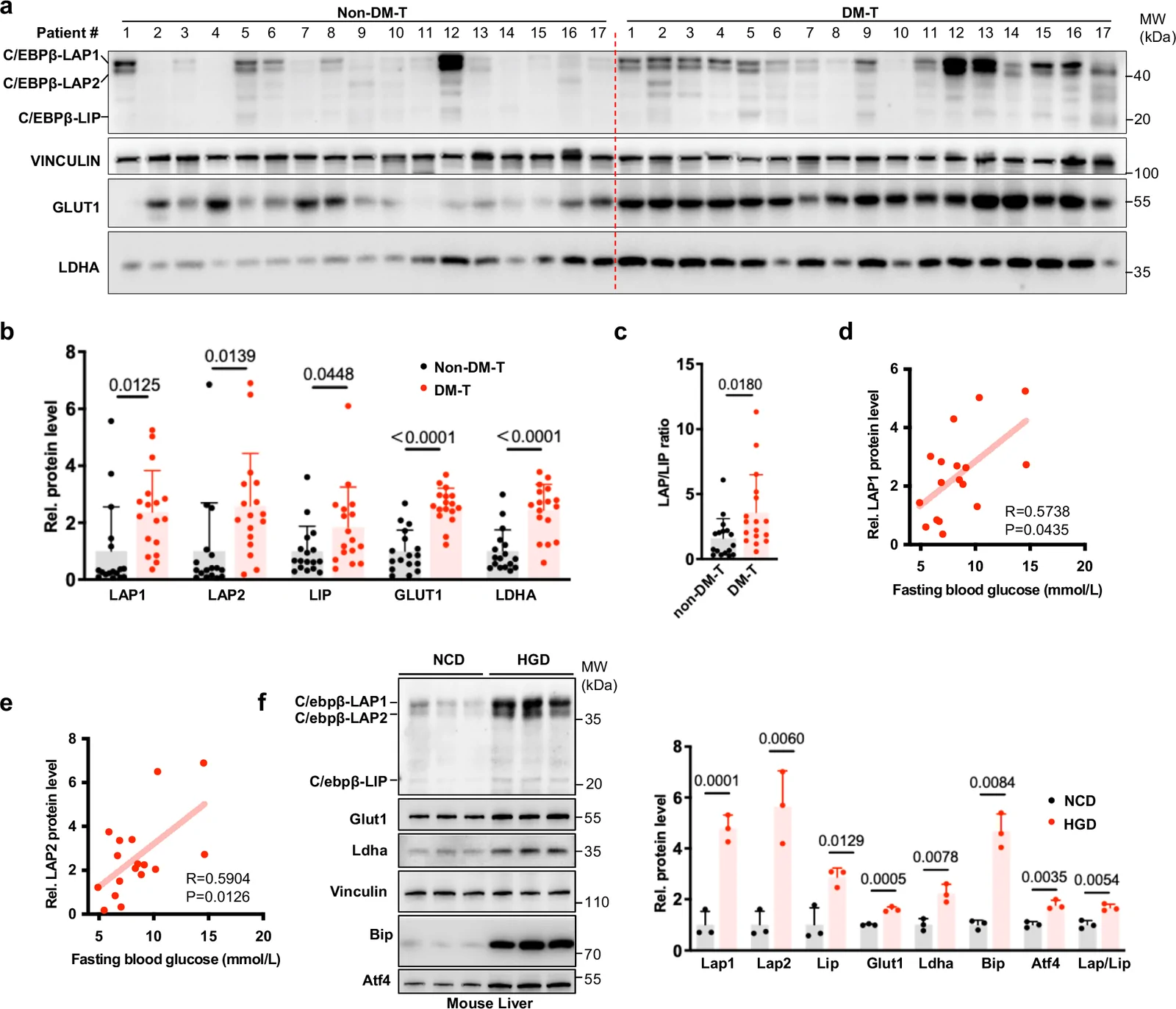
Increased hepatic C/EBPβ expression in patients with DM. **a**, **b** Immunoblotting (**a**) and Quantification (**b**) in tumor (T) from Hepatocellular Carcinoma (HCC) patients with or without Diabetes mellitus (DM). (*N* = 17 patients) *p* values for each Non-DM-T vs. DM-T comparison were: GLUT1 *p* = 1.05E-07; LDHA, *p* = 1.68E-05. **c** The LAP/LIP ratio in tumor from HCC patients with or without DM. LAP/LIP ratio was calculated by adding LAP1 and LAP2 and dividing this value by LIP. (N = 17 patients). **d**,** e** Correlation analysis of FPG (fasting plasma glucose) levels and C/EBPβ-LAP1 (**d**) /LAP2 (**e**) levels of tumor tissue in HCC patients. The regression slope (center line), the correlation coefficient (R) and *P* value are shown in the graph. (N = 17 patients). **f** Immunoblotting (left) and Quantification (right) in liver samples from normal chow diet (NCD) treated mice and high glucose diet treated mice (HGD). (N = 3 mice). Data are mean ± SD. *P* values were computed by unpaired t-test (two-sided) (**b**–**f**). Source data are provided as a Source Data file.

To investigate the effect of hyperglycemia or diabetes mellitus (DM) on cancer progression, we analyzed liver tissues from hepatocellular carcinoma (HCC) patients with pre-existing diabetes mellitus (DM) and paired non-diabetic HCC controls (patient characteristics summarized in Supplementary Table 1-3). Compared to non-diabetic counterparts, both tumor and paracancerous tissues from diabetic patients exhibited markedly elevated protein levels of LAPs (LAP1 and LAP2), LIP, and LAP/LIP ratio (Fig. 1a–c, Supplementary Fig. 1a, b, and Supplementary Fig. 2a–c), along with increased C/EBPβ mRNA abundance (Supplementary Fig. 1c). Notably, key glycolysis effectors (GLUT1, LDHA) were also upregulated in both tumor and paracancerous tissues from diabetic patients, compared with non-diabetic controls (Fig. 1a, b). T-cell infiltration and inflammatory effects (IL-6 and TNFα) were comparable between the DM and non-DM groups, suggesting that these increases were not caused by differences in the inflammatory microenvironment (Supplementary Fig. 1a, b). Strikingly, the levels of all C/EBPβ isoforms in paracancerous tissues correlated with fasting plasma glucose levels in these HCC patients (Supplementary Fig. 3a–c). In tumor tissues, C/EBPβ-LAPs, but not C/EBPβ-LIP, correlated with fasting plasma glucose levels (Fig. 1d, e, and Supplementary Fig. 3d). Clinically, although C/EBPβ mRNA does not increase in HCC patients, high C/EBPβ expression was associated with worse survival in HCC and colon adenocarcinoma patients, malignancies exhibiting strong epidemiological links to hyperglycemia or DM^2^ (Supplementary Fig. 4a–c). These findings strongly implicate C/EBPβ, specifically its LAP isoforms, as central mediators of hyperglycemia-induced glycometabolic reprogramming that fuels cancer progression, though the underlying mechanisms require full elucidation.

Consistent with human data, high glucose diet (HGD)-fed mice (which develop sustained hyperglycemia as previously reported^6,21,22^) and STZ-induced type 1 DM mice showed significantly upregulated hepatic C/EBPβ isoforms compared with controls (Fig. 1f and Supplementary Fig. 1d, e). In human hepatocellular carcinoma HepG2 cells and human colon cancer HCT116 cells, C/EBPβ-LAP levels increased time-dependently upon high-glucose exposure and decreased during glucose starvation (Supplementary Fig. 5a–f). Based on these conserved regulatory patterns, we then examined C/EBPβ‘s functional roles in hyperglycemia-driven cancer progression using these models.

### Glucose stimulates C/EBPβ transcription via ROS-dependent PERK-eIF2α-ATF4 axis

**Fig. 2:**
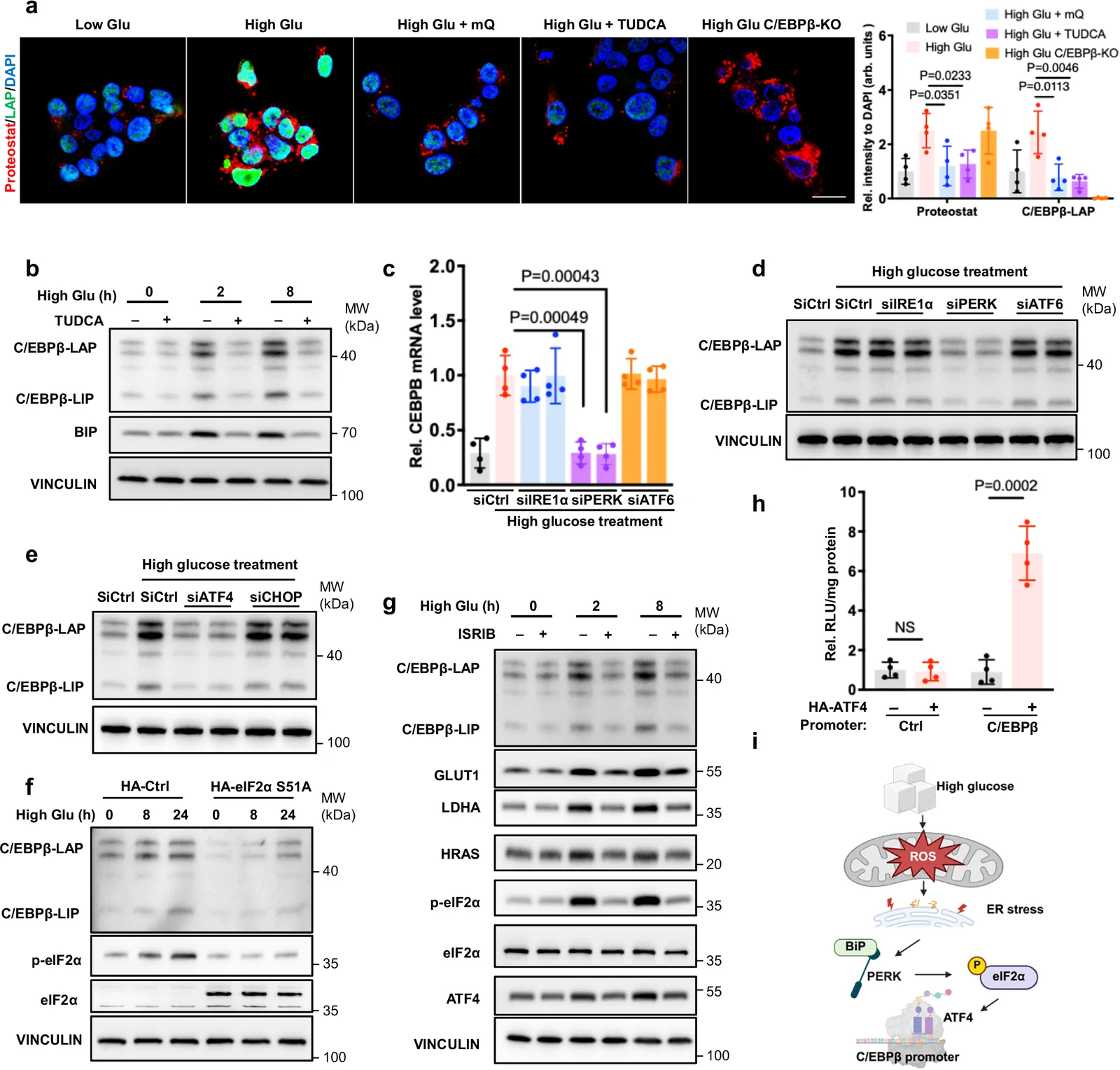
Glucose stimulates C/EBPβ transcription via PERK-eIF2α-ATF4 axis. **a** (left) Representative IF images of WT or C/EBPβ-KO HepG2 cells treated with low glucose (2.8 mM, 8 h), high glucose (12.8 mM, 8 h), high glucose + mQ (0.4 μM) and high glucose + TUDCA (100 μM). Scale bar, 20 μm (right) Quantification of Proteostat and C/EBPβ-LAP. arb. units, arbitrary units. (N  =  4 independent experiments). **b** Immunoblotting in high glucose-treated (0, 2 and 8 h) HepG2 cells, with/without TUDCA (100 μM). **c**,** d** Relative CEBPB mRNA levels (**c**) (N  =  4 independent experiments) and Immunoblotting (**d**) in siCtrl, siIRE1α, siPERK and siATF6 HepG2 cells, with/without high glucose treatment (12.8 mM, 8 h). **e** Immunoblotting in siCtrl, siATF4 and siCHOP HepG2 cells, with/without high glucose treatment (12.8 mM, 8 h). **f** Immunoblotting in high glucose-treated (0, 2 and 8 h) HepG2 cells, transfected with HA-Ctrl or HA-eIF2α S51A. **g** Immunoblotting in high glucose-treated (0, 2 and 8 h) HepG2 cells, with/without ISRIB (250 nM). **h** Relative RLUs (Relative Light Units)/mg protein of Ctrl and CEBPB promoters in HEK293 cells transfected with Ctrl or HA-ATF4. (N  =  4 independent experiments). **i** Schematic illustration showing that glucose stimulates C/EBPβ through ROS-ER stress-PERK-eIF2α-ATF4 axis. Data are mean ± SD. P values were computed by unpaired t-test (two-sided) (**a**, **c**, **h**). NS, non-significant. Schematic in **i** was created in BioRender. L, Y. (https://BioRender.com/2mhqokc). Source data are provided as a Source Data file.

We were particularly curious about how hyperglycemia triggers C/EBPβ activation. The major signaling pathways in cells driven by hyperglycemia associate with the production and accumulation of reactive oxygen species (ROS) through mitochondrial respiratory chain enzymes, typically inducing endoplasmic reticulum (ER) stress^23,24^. While ROS (e.g., hydrogen peroxide (H_2_O_2_), hypoxia) and ER stress (e.g., tunicamycin, Akita diabetic model) have been linked to C/EBPβ activation, glucose-specific regulatory mechanisms of C/EBPβ remained undefined^13,25–27^. Accordingly, we validated that high glucose robustly increases ROS (measured by H_2_DCFDA staining), which was rescued by therapeutic antioxidant mitoquinol (mQ) treatment (Supplementary Fig. 6a)^28,29^. In line with this, clearance of ROS by mQ treatment blocked high glucose-induced C/EBPβ isoforms upregulation, validated by immunofluorescence and immunoblotting (Fig. 2a and Supplementary Fig. 6b). ER stress is usually associated with increased misfolded/aggregated proteins (detected by Proteostat staining) and upregulated BiP (also known as GRP78), a widely known molecular chaperone as an important ER stress sensor^30,31^. We found that high glucose treatment indeed elevated misfolded/aggregated proteins, BiP expression, and C/EBPβ levels in HepG2 cells (Fig. 2a, b). Crucially, alleviating ER stress with tauroursodeoxycholic acid (TUDCA) abolished glucose-induced C/EBPβ upregulation (Fig. 2a, b)^32^. Notably, C/EBPβ deletion did not directly alter misfolded/aggregated protein accumulation, positioning ER stress upstream of C/EBPβ (Fig. 2a). These data suggest that glucose upregulates C/EBPβ via ROS-dependent ER stress.

We next dissected ER stress signaling. ER stress activates the unfolded protein response (UPR) through three well-established ER stress sensors: IRE1α, PERK, and ATF6^31,33^. Among these, only PERK knockdown prevented the elevation of C/EBPβ expression at mRNA/protein levels owing to high glucose treatment (Fig. 2c, d and Supplementary Fig. 6c). PERK signals via eIF2α phosphorylation to activate ATF4 or CHOP^31,34^. Dominant-negative eIF2α (S51A) or *ATF4* knockdown (but not *CHOP* depletion) suppressed high glucose-induced C/EBPβ expression (Fig. 2e, f and Supplementary Fig. 6f, g). Moreover, we further employed ISRIB, a PERK pathway inhibitor, to counteract the effects of eIF2α phosphorylation^35^. Similarly, ISRIB treatment significantly prevented high glucose-induced C/EBPβ expression, associated with down-regulation of ATF4, GLUT1, LDHA and HRAS (Fig. 2g). Luciferase reporter assays confirmed direct transcriptional activation of the C/EBPβ promoter by ATF4 (Fig. 2h). In addition, we validated the upregulation of BIP and ATF4 in HCC tissues from the DM group versus non-DM controls (Supplementary Fig. 1a, b). In HGD mice and STZ-induced Type1 DM mouse livers, BIP and ATF4 expression also increased compared with that in control mice (Fig. 1f and Supplementary Fig. 1e). In contrast, inhibiting NF-κB or mTOR (other well-studied nutrient-responsive regulators) showed no effect on glucose-induced LAP upregulation, suggesting that these regulators are not critical in this process (Supplementary Fig. 6d, e)^36,37^. Thus, we identified a critical ROS-PERK-eIF2α-ATF4 axis driving C/EBPβ transcription in hyperglycemia (illustrated in Fig. 2i).

### C/EBPβ-LAP isoforms drive glycolysis and proliferation in liver cancer cells

**Fig. 3:**
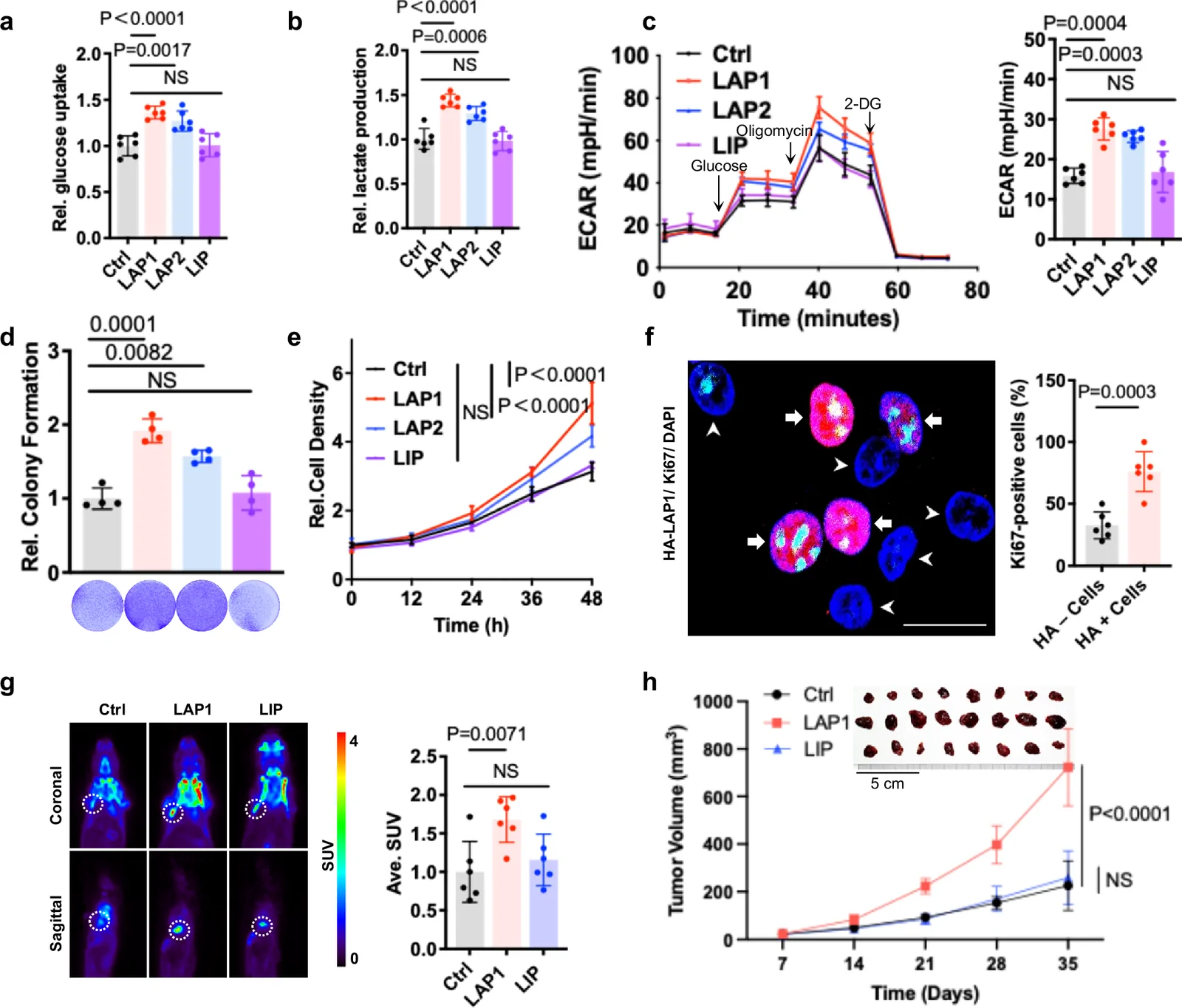
C/EBPβ-LAPs promote glycolysis and proliferation in liver cancer cells. **a**, **b** Relative glucose uptake (**a**) and lactate production (**b**) of HepG2 cells overexpressing C/EBPβ-LAP1/2 or C/EBPβ-LIP. (N = 6 independent experiments) *p* values for Ctrl vs. LAP1 comparison were: glucose uptake, *p* = 4.49E-05; lactate production, *p* = 1.55E-05. **c** (left) Extracellular acidification rates (ECARs) of HepG2 cells expressing C/EBPβ-LAP1/2 or C/EBPβ-LIP in response to glucose, oligomycin, and 2-deoxy-D-glucose (2-DG). (right) ECARs in response to glucose, which indicate glycolysis, of HepG2 cells expressing C/EBPβ-LAP1/2 or C/EBPβ-LIP. (N = 6 independent experiments). **d**,** e** Relative colony formation (**d**) and growth curve (**e**) of HepG2 cells expressing C/EBPβ-LAP1/2 or C/EBPβ-LIP. (N = 4 independent experiments) *p* values for growth curve: Ctrl vs. LAP1 comparison, *p* = 0.000002; Ctrl vs. LAP2 comparison, *p* = 0.000027. **f** (left) Representative IF image of HepG2 cells transfected with HA-LAP1. (right) Ki67-positive percentage of HepG2 cells with or without HA-LAP1. Arrowheads represent HA-LAP1 positive cells; Arrows represent HA-LAP1 negative cells. Scale bar, 20 μm. (N = 6 independent experiments). **g** (left) Representative ^18^F-FDG PET images of in vivo xenograft tumors of C/EBPβ-LAP1 or C/EBPβ-LIP HepG2 cells 14 days post-subcutaneously inoculation in nude mice. The xenograft tumors are circled. (right) Average SUV (Standardized Uptake Value) representing the activity of glucose metabolism of the xenograft tumor. (N = 6 mice). **h** Tumor growth curve of in vivo xenograft tumor of C/EBPβ-LAP1 or C/EBPβ-LIP HepG2 cells subcutaneously inoculated in nude mice (N = 8 mice). *p* value for Ctrl vs. LAP1 comparison: *p* = 1E-15. Data are mean ± SD. *P* values were computed by unpaired t-test (two-sided) (**a**–**d**, **f**, **h**) or two-way ANOVA (two-sided) (**e**, **g**). NS, non-significant. Source data are provided as a Source Data file.

To determine whether C/EBPβ directly regulates glycolysis, which is the vital glycometabolic process providing rapid energy and essential precursors for cancer cells^10^, we generated HepG2 cells stably overexpressing the LAP1, LAP2 or LIP isoform (termed LAP1-HepG2, LAP2-HepG2 or LIP-HepG2, respectively). LAP1/2-HepG2 cells exhibited significantly enhanced glycolysis, evidenced by increased glucose uptake, lactate production, and extracellular acidification rates (ECAR) compared with control cells or LIP-HepG2 cells (Fig. 3a–c). This metabolic reprogramming was accompanied by elevated cancer cell survival and proliferation in LAP1/2-HepG2 cells (Fig. 3d, e). Consistent with this, HA-tagged LAP1 transfection in HepG2 cells increased Ki67-positive cell proportion, confirming accelerated proliferation (Fig. 3f). Notably, in the HepG2 xenograft tumorigenesis model, LAP1-HepG2 robustly enhanced tumor glycometabolism and tumorigenesis, while LIP overexpression showed no significant effect (Fig. 3g, h). Thus, C/EBPβ-LAP isoforms, but not LIP isoform, is a specific driver of glycolytic flux and proliferative expansion in liver cancer.

### C/EBPβ-LAP isoforms transcriptionally activate core glycolytic effectors

**Fig. 4:**
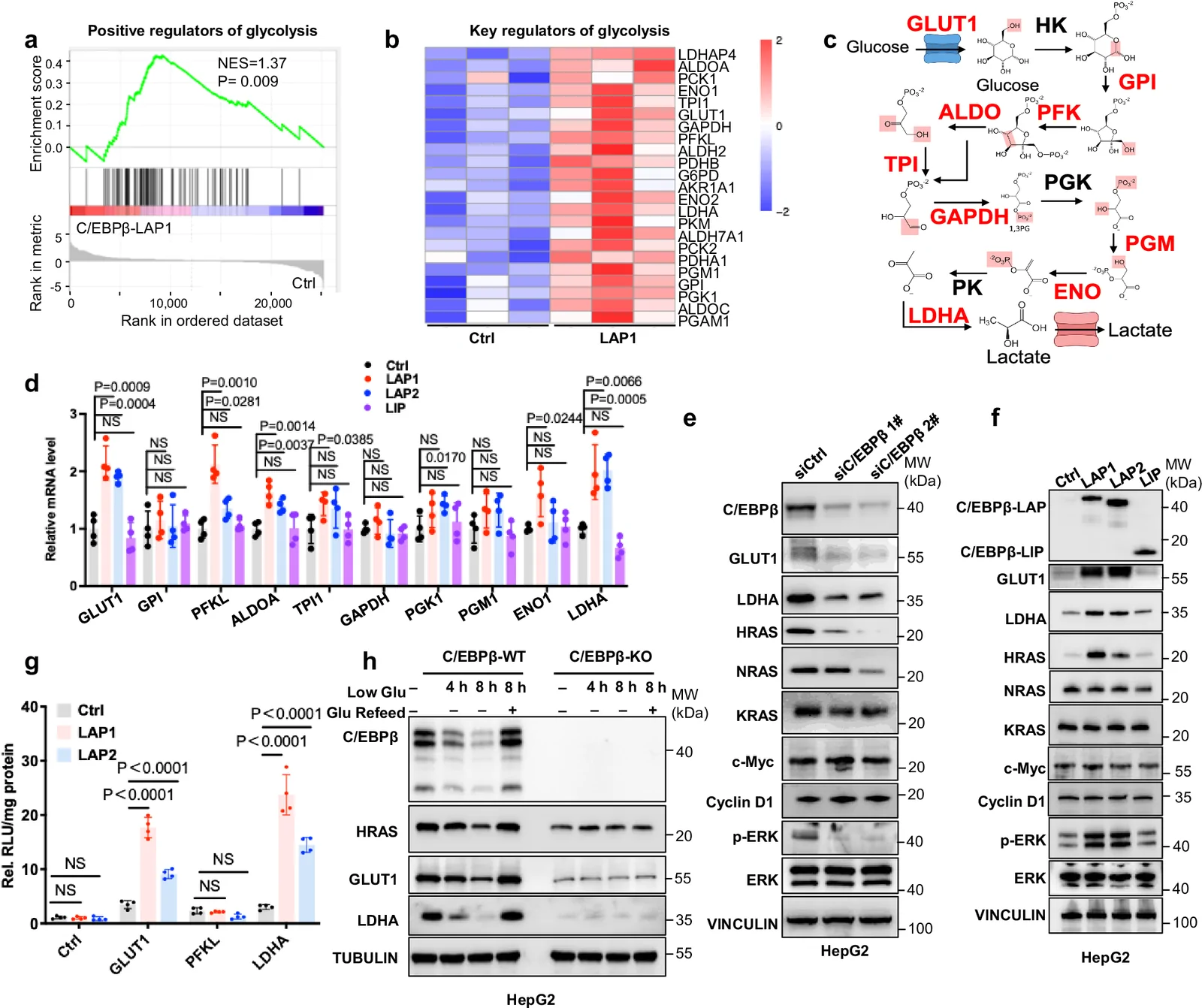
C/EBPβ-LAPs upregulate the expression of key glycolytic genes and HRAS in liver cancer cells. **a** Gene set enrichment analysis (GSEA) showing the positive glycolytic gene signature in the RNA-seq results of HepG2 cells with or without LAP1 overexpression. NES (Normalized Enrichment Score) and *p* value are shown in the graph. *P* values were computed by permutation Tests. **b** Heatmap showing the normalized expression of the key glycolytic genes in the RNA-seq results of HepG2 cells with or without LAP1 overexpression. **c** Schematic illustration showing the process of glycolysis. Key glycolytic processes upregulated in the RNA-seq results in LAP1 HepG2 cells over control cells are highlighted in red. **d** Relative mRNA levels of key glycolytic genes in Ctrl, LAP1/2 and LIP-overexpressing HepG2 cells. The genes most upregulated in LAP-HepG2 are highlighted in red. (N = 4 independent experiments). **e** Immunoblotting in Ctrl, LAP1/2 and LIP-overexpressing HepG2 cells. **f** Immunoblotting in siCtrl, siC/EBPβ 1# and siC/EBPβ 2# cells. **g** Relative RLUs (Relative Light Units) /mg protein of Ctrl, GLUT1, PFKL and LDHA promoters in HEK293 cells transfected with Ctrl, LAP1/2 or LIP. (N = 4 independent experiments) *p* values for Ctrl vs. LAP1 comparison: GLUT1, *p* = 8.0E-06; LDHA, *p* = 3.1E-05. *p* values for Ctrl vs. LAP2 comparison: GLUT1, *p* = 5.4E-05; LDHA, *p *= 1.8E-06. **h** Immunoblotting in C/EBPβ-WT and C/EBPβ-KO HepG2 cells starved by low glucose (2.8 mM) at the indicated time, with/without glucose restoration (12.8 mM, 4 h) afterwards. Data are mean ± SD. *P* values were computed by unpaired t-test (two-sided) (**d**, **g**). NS non-significant. Source data are provided as a Source Data file.

To gain insight into the gene signatures directly mediated by LAPs for cancer cell progression, we performed RNA-seq in LAP1-HepG2 versus control cells. The upregulated DEGs (differentially expressed genes) in LAP1-HepG2 were enriched in various pathways related to glycometabolism (Supplementary Fig. 7a). Consistent with our earlier observations, gene set enrichment analysis (GSEA) revealed significant enrichment of positive regulators of glycolysis in LAP1-HepG2 cells compared with control cells (Fig. 4a). Critically, key glycolytic genes spanning multiple steps of glycolysis, including *ALDOA*, *TPI1*, *ENO1*, *LDHA*, *SLC2A1* (encoding GLUT1), and *PFKL*, were upregulated in LAP1-HepG2 cells (Fig. 4b, c). These findings were also further validated by RT-qPCR, with GLUT1, PFKL and LDHA exhibiting the most significant upregulation (Fig. 4d). Luciferase reporter assays demonstrated that LAP1/2 directly transcriptionally activates the GLUT1 and LDHA promoters, while PFKL activation was indirect (Fig. 4g). Consistently, LAP1/2 overexpression elevated GLUT1 and LDHA protein levels in HepG2 cells, whereas C/EBPβ knockdown ablated their expression (Fig. 4e, f). In contrast, LIP failed to modulate these targets at either mRNA or protein levels (Fig. 4d, e). Importantly, glucose-dependent induction of GLUT1 and LDHA required C/EBPβ, as their upregulation was abolished in C/EBPβ-deficient HepG2 cells or HCT116 cells (Fig. 4h and Supplementary Fig. 7b). We also observed that, in human HCC samples, GLUT1 and LDHA protein levels positively correlated with LAP1 protein levels, but not with LIP protein levels (Supplementary Fig. 3e–j). These results strongly support that C/EBPβ-LAPs drive functional glycolysis by directly mediating the transcription of various key glycolytic effectors (GLUT1 and LDHA).

### C/EBPβ-LAPs transcriptionally activate the expression of oncogene HRAS to drive cancer cell proliferation

While glycolysis supports cancer progression through providing rapid energy production, oncogene activation is also essential for cell division and growth^10,38^. RAS and other oncogenes, particularly *HRAS*, are unusually upregulated in DM patients and animal models, though the underlying mechanisms remain incompletely understood^39–42^. We therefore asked whether C/EBPβ also mediates oncogene expression to drive hyperglycemia-induced cancer proliferation. Strikingly, C/EBPβ depletion selectively suppressed HRAS (but not KRAS, NRAS, c-Myc or Cyclin D1), concomitant with reduced downstream phosphorylated ERK signaling (Fig. 4e and Supplementary Fig. 7c). Conversely, LAP1/2 overexpression significantly increased HRAS expression and ERK phosphorylation, while LIP had no effect (Fig. 4f and Supplementary Fig. 7d). Luciferase reporter and ChIP assays confirmed that C/EBPβ directly binds to and activates both the human *HRAS* and mouse *Hras* promoters, and this activation was abolished by mutations in the binding sites (Supplementary Fig. 7e–g). Moreover, C/EBPβ depletion abolished glucose-responsive HRAS activation in both HepG2 cells and HCT116 cells (Fig. 4h and Supplementary Fig. 7b). We confirmed elevated HRAS expression in HCC patients with diabetes versus non-diabetic controls (Supplementary Fig. 1a, b). HRAS was specifically upregulated and its upregulation was associated with worse survival in HCC patients (Supplementary Fig. 7h, i). Therefore, these results establish C/EBPβ-LAPs as a dual regulator of hyperglycemia-driven cancer progression: orchestrating metabolic reprogramming through key glycolytic regulators while directly activating the oncogene HRAS to ignite cell proliferation.

### C/EBPβ is required for hyperglycemia-driven hepatocellular carcinoma progression

**Fig. 5:**
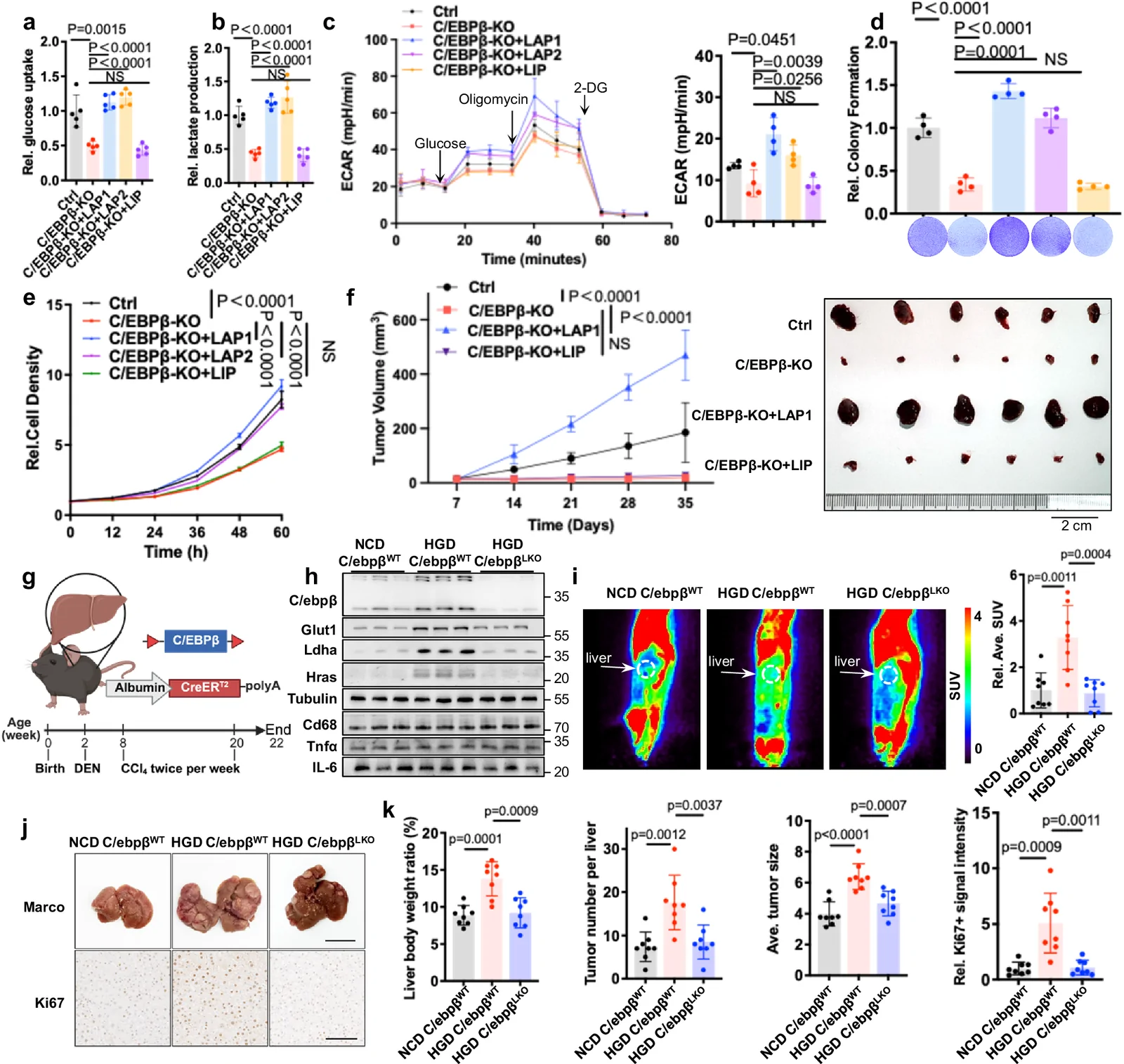
C/EBPβ is required for hyperglycemia-driven hepatocellular carcinoma development. **a**,** b** Relative glucose uptake (**a**) and lactate production (**b**) of Ctrl, C/EBPβ-KO, C/EBPβ-KO + LAP1/LAP2 and C/EBPβ-KO + LIP HepG2 cells. (N = 5 independent experiments) *p* values for C/EBPβ-KO vs. C/EBPβ-KO + LAP1 comparison were: glucose uptake, *p* = 4.70E-05; lactate production, *p* = 2.09E-07. *p* values for C/EBPβ-KO vs. C/EBPβ-KO + LAP2 comparison were: glucose uptake, *p* = 2.19E-06; lactate production, *p* = 6.45E-05. **c** (left) Extracellular acidification rates (ECARs) of Ctrl, C/EBPβ-KO, C/EBPβ-KO + LAP1/LAP2 and C/EBPβ-KO + LIP HepG2 cells in response to glucose, oligomycin, and 2-deoxy-D-glucose (2-DG). (right) ECARs in response to glucose, which indicate glycolysis, of the HepG2 cells. (N = 4 independent experiments). **d**,** e** Relative colony formation (**d**) and growth curve (**e**) of Ctrl, C/EBPβ-KO, C/EBPβ-KO + LAP1/LAP2 and C/EBPβ-KO + LIP HepG2 cells. (N = 4 independent experiments) *p* values for Ctrl vs. C/EBPβ-KO comparison were: colony formation, *p* = 7.3E-05; growth curve, *p* = 5.4E-12. *p* values for C/EBPβ-KO vs. C/EBPβ-KO + LAP1 comparison were: colony formation, *p* = 1.5E-06; growth curve, *p* = 4.3E-15. *p* values for C/EBPβ-KO vs. C/EBPβ-KO + LAP2 comparison were: colony formation, *p* = 2.9E-05; growth curve, *p* = 1.8E-14. **f**, Tumor growth curve (left) and tumor images at end point (right) of in vivo xenograft tumor of Ctrl, C/EBPβ-KO, C/EBPβ-KO + LAP1 and C/EBPβ-KO + LIP HepG2 cells subcutaneously inoculated in nude mice (N = 6 mice). *p* values for Ctrl vs. C/EBPβ-KO comparison: *p* = 6.4E-05; for C/EBPβ-KO vs. C/EBPβ-KO + LAP1: *p* = 4.4E-06. **g** Schematic illustration of hepatocyte-specific C/ebpβ-KO mouse model (top) and carcinogen (DEN/CCl_4_)-induced HCC model (bottom). **h** Immunoblotting in liver samples from normal chow diet (NCD) treated mice and high glucose diet (HGD) treated C/ebpβ^WT^ or C/ebpβ^LKO^ mice. **i** (left) Representative ^18^F-FDG PET images of NCD C/ebpβ^WT^, HGD C/ebpβ^WT^ and HGD C/ebpβ^LKO^ mice. (right) Relative average SUV (Standardized Uptake Value) representing the activity of glucose metabolism of the liver. (N = 8 mice). **j** Representative gross images of liver pathology and hepatic Ki67 IHC staining of NCD C/ebpβ^WT^, HGD C/ebpβ^WT^ and HGD C/ebpβ^LKO^ mice. Scale bar, (top) 1 cm or (bottom) 100 μm. **k** Quantifications of liver/body weight ratio, tumor number, tumor size, and Ki67 signal intensity in the tumor samples. (N = 8 mice) p-value for NCD C/ebpβ^WT^ vs HGD C/ebpβ^WT^ comparison were: ave. tumor size, *p* = 3.3E-05. Data are mean ± SD. P values were computed by unpaired t-test (two-sided) (**a**–**d**, **i**, **k**) or two-way ANOVA (two-sided) (**e**, **f**). NS, non-significant. Schematic in (**g**) was Created in BioRender. L, Y. (https://BioRender.com/pgvjae2). Source data are provided as a Source Data file.

Next, we determined whether C/EBPβ is the dominant regulator of hyperglycemia-induced cancer progression. RNA-seq analysis revealed that C/EBPβ-KO HepG2 cells exhibit transcriptional repression of glycolytic effectors (e.g., GLUT1 (*SLC2A1*), LDHA) and proliferative drivers (e.g., HRAS) (Supplementary Fig. 8a). Consistently, C/EBPβ deletion ablated glycolysis (reducing glucose uptake, lactate production and extracellular acidification rate (ECAR)), and suppressed cancer cell survival and proliferation in HepG2 cells; these effects were rescued by LAP1/2, but not LIP restoration (Fig. 5a–e). Moreover, C/EBPβ knockout attenuated HepG2 tumorigenicity in xenografts, reversible by LAP1 restoration (Fig. 5f). To investigate the in vivo mechanism of hyperglycemia-driven hepatocellular carcinoma (HCC) progression, we employed a N-nitrosodiethylamine/carbon tetrachloride (DEN/CCl4)-induced HCC mouse model that recapitulates key features of human HCC pathogenesis, including inflammation and fibrogenesis^43–45^. To examine the hepatocyte-specific C/EBPβ function in vivo, we restricted deletion of C/ebpβ in hepatocytes with a tamoxifen-inducible Albumin-CreER^T2^ allele in this HCC model (C/ebpβ^LKO^) (Fig. 5g). Hepatic C/ebpβ depletion in HGD-fed C/ebpβ^LKO^ mice was confirmed, coinciding with reduced Glut1, Ldha, and Hras expression, compared with HGD-fed controls (Fig. 5h and Supplementary Fig. 8c). Of Note, C/ebpβ^LKO^ mice showed no significant differences in macrophage infiltration, inflammatory effects (Il-6 and Tnfα) and fasting plasma glucose levels versus C/ebpβ^WT^ mice, suggesting that these increases were not caused by differences in the inflammatory microenvironment or fasting plasma glucose regulation (Fig. 5h and Supplementary Fig. 8b, c). ^18^FDG-PET scanning showed that while HGD upregulated hepatocellular glycometabolism, this effect was abolished by hepatocyte-specific C/ebpβ deletion (Fig. 5i). Critically, biallelic C/ebpβ deletion prevented HGD-induced acceleration of HCC progression and hepatocellular Ki67 positivity (Fig. 5j, k). Therefore, loss of hepatic C/ebpβ alleviates hyperglycemia-fueled HCC progression.

### C/EBPβ-LAP1 upregulates glycolytic genes, *Hras*, and fuels HCC development

**Fig. 6:**
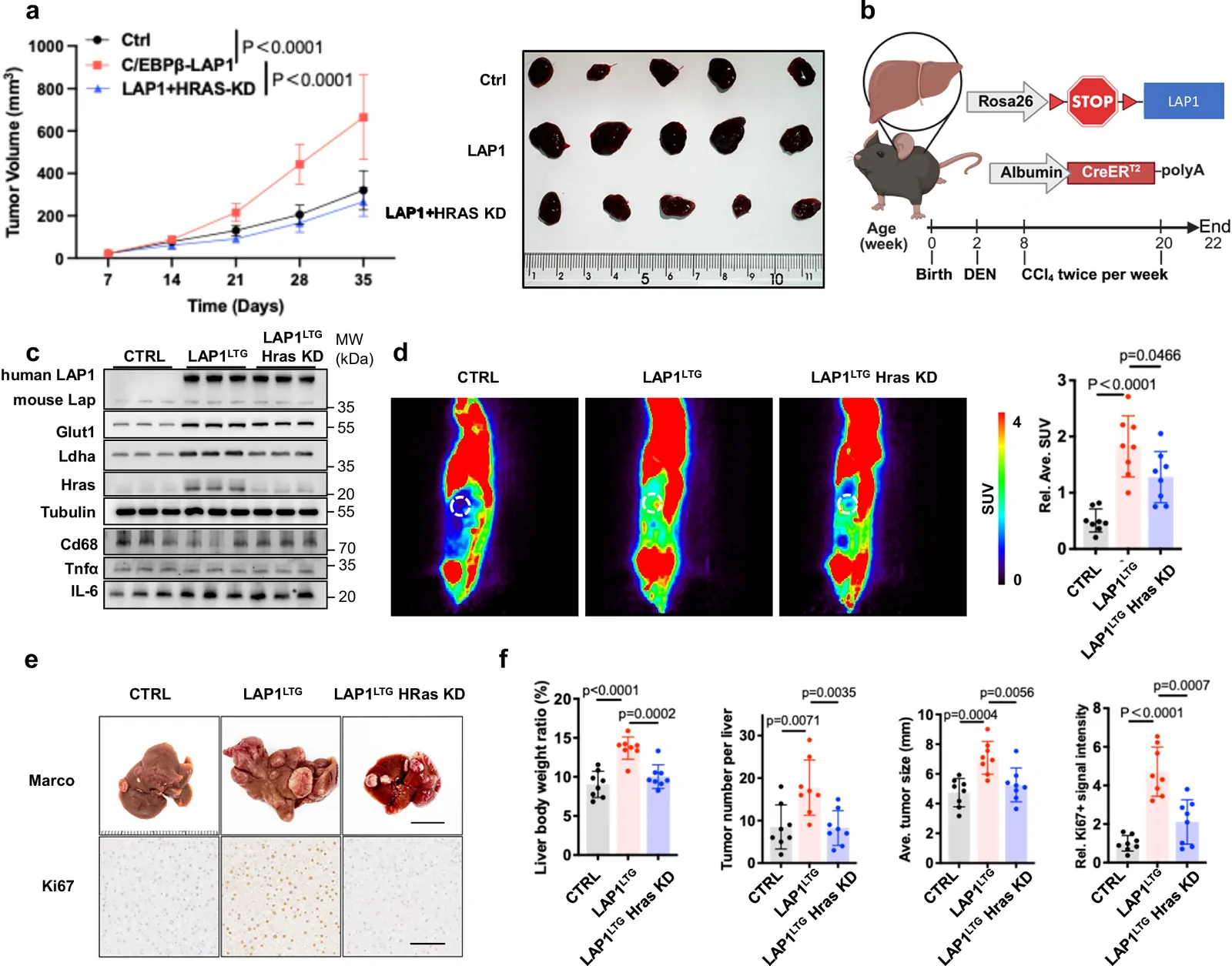
C/EBPβ-LAP1 upregulates glycolytic genes, Hras, and fuels HCC development. **a** Tumor growth curve (left) and tumor images at end point (right) of in vivo xenograft tumor of Ctrl, C/EBPβ-LAP1 and C/EBPβ-LAP1 + HRAS-KD HepG2 cells subcutaneously inoculated in nude mice (N = 5 mice). *p* value for Ctrl vs C/EBPβ-LAP1comparison: p = 8.5E-11; for C/EBPβ-LAP1 vs C/EBPβ-LAP1 + HRAS-KD: *p* = 1.2E-09. **b** Schematic illustration of hepatocyte-specific human LAP1-transgenic mouse model (top) and carcinogen (DEN/CCl_4_)-induced HCC model (bottom). **c** Immunoblotting in liver samples from Ctrl, LAP1^LTG^ and LAP1^LTG^ Hras-KD mice. **d** (left) Representative ^18^F-FDG PET images of Ctrl, LAP1^LTG^ and LAP1^LTG^ Hras-KD mice. (right) Relative average SUV (Standardized Uptake Value) representing the activity of glucose metabolism of the liver. (N = 8 mice) *p* value for Ctrl vs. LAP1^LTG^ comparison: *p* = 3.1E-05. **e**,** f** (**e**) Representative gross images of liver pathology and hepatic Ki67 IHC staining of Ctrl, LAP1^LTG^ and LAP1^LTG^ Hras-KD mice. Scale bar, (top) 1 cm or (bottom) 100 μm. **f** Quantifications of liver/body weight ratio, tumor number, tumor size, and Ki67 signal intensity in the tumor samples. (N = 8 mice) *p* value for Ctrl vs LAP1^LTG^ comparison were: liver body weight ratio, *p* = 0.00007; ki67 positive signal intensity, *p* = 1.7E-06. Data are mean ± SD. *P* values were computed by unpaired t-test (two-sided) (**d**, **f**) or two-way ANOVA (two-sided) (**a**). NS, non-significant. Schematic in (**b**) was created in BioRender. L, Y. (https://BioRender.com/pgvjae2). Source data are provided as a Source Data file.

We next assessed whether LAPs accelerate HCC development in vivo and defined the contribution of HRAS during the process. Given the high sequence homology (93.4%), functional similarity, and equivalent expression patterns between LAP1 and LAP2, we focused on LAP1 as the representative isoform of LAPs. Remarkably, HRAS knockdown abrogated the elevated tumor survival and growth owing to LAP1 overexpression in HepG2 cells, without significantly affecting LAP1-induced glycolysis (Supplementary Fig. 9a–e). Consistently, HRAS depletion compromised LAP1-driven tumorigenesis in HepG2 xenografts (Fig. 6a). We further generated inducible hepatocyte-specific LAP1 transgenic mice (LAP1^LTG^) with DEN/CCl_4_-induced HCC model. These mice conditionally expressed Myc-tagged human LAP1 from a loxP-stop-loxP (LSL) cassette targeted to the Rosa26 locus upon tamoxifen induction (Fig. 6b). The livers of LAP1^LTG^ mice exhibited robust human LAP1 expression, together with elevated Glut1, Ldha and Hras levels, but not Cd68, Tnfα or IL-6, versus control mice (C/ebpβ^WT^) (Fig. 6c and Supplementary Fig. 9f). To knock down *Hras* in LAP1^LTG^ mice (LAP1^LTG^ Hras KD), we used adeno-associated virus (AAV) delivering *Hras* siRNA under the liver-specific TBG promoter. This reduced hepatic Hras expression without altering endogenous C/ebpβ, exogenous human LAP1, or GLUT1/LDHA levels (Fig. 6c). Phenocopying HGD treatment, NCD-fed LAP1^LTG^ mice displayed escalated hepatocellular glycometabolism, accelerated HCC progression, and increased hepatocellular Ki67 signals versus controls (Fig. 6d–f). Notably, eliminating Hras was sufficient to prevent the enhanced HCC progressions and hepatocellular Ki67 signals owing to LAP1 overexpression in these mice, and slightly attenuated LAP1-induced hepatocellular ^18^FDG-PET signal (Fig. 6d–f). Together, these results solidly demonstrate that LAP recapitulates glucose-fueled HCC progression by activating glycolytic and proliferative programs, with HRAS serving as the essential downstream effector for proliferation (illustrated in Supplementary Fig. 11).

### Pharmacological inhibition of C/EBPβ by ST101 attenuates hyperglycemia-driven HCC progression

**Fig. 7:**
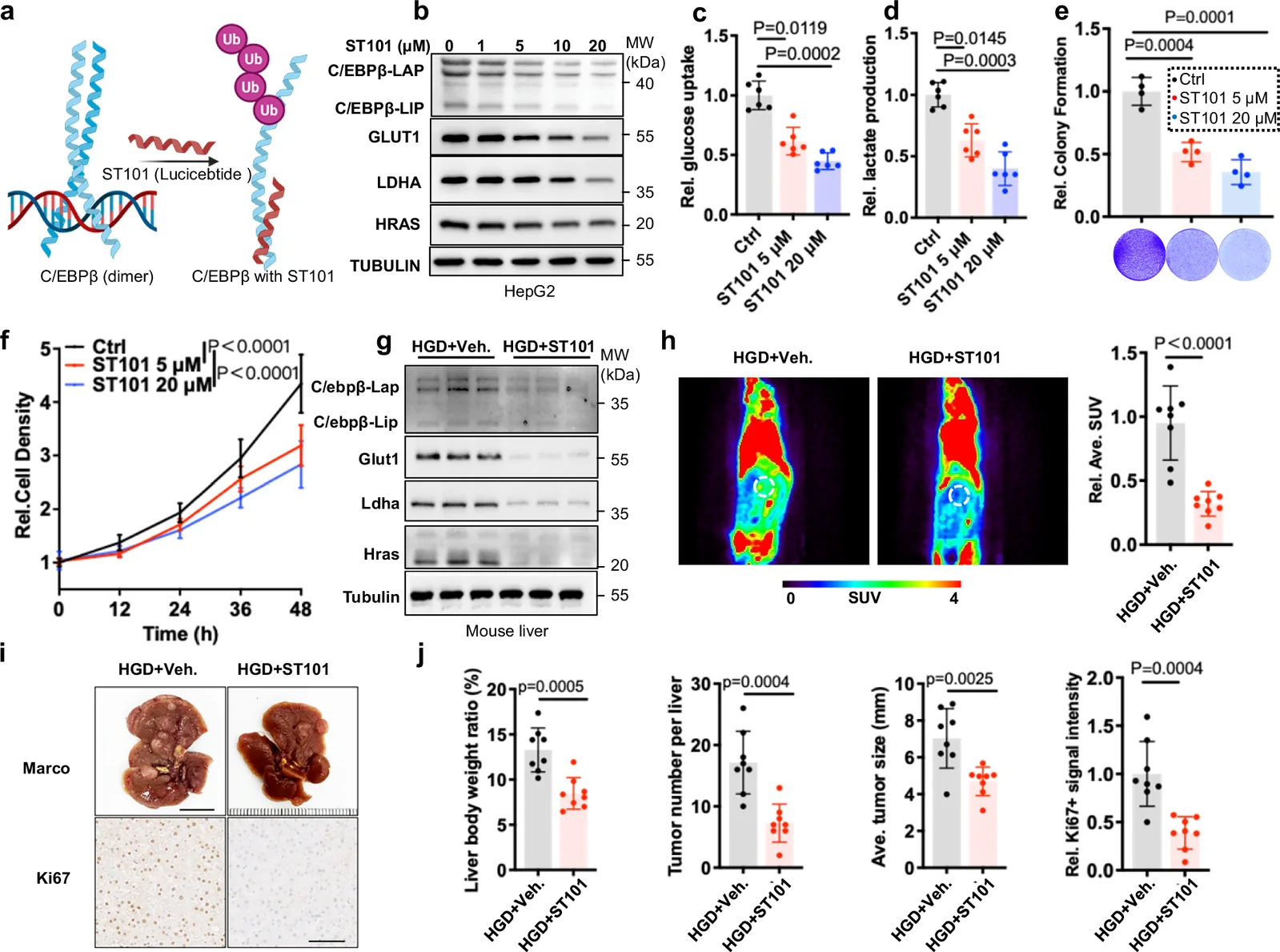
C/EBPβ inhibition by ST101 attenuates glucose-driven HCC development. **a** Schematic illustration of ST101 (Lucicebtide) working mechanism. Schematic was created in BioRender. L, Y. (https://BioRender.com/0mpgnhk). **b** Immunoblotting in HepG2 cells treated with ST101 for 24 h at the indicated doses. **c**, **d** Relative glucose uptake (**c**) and lactate production (**d**) of HepG2 cells with/without ST101 treatment. (N = 6 independent experiments). **e**, **f** Relative colony formation (**e**) and growth curve (**f**) of HepG2 cells with/without ST101 treatment. (N = 4 independent experiments). **g** Immunoblotting in liver samples from high glucose diet (HGD) mice treated with/without ST101 (25 mg/kg administered three days per week by IV injection). **h** (left) Representative ^18^F-FDG PET images of HGD mice treated with/without ST101.(right) Relative average SUV (Standardized Uptake Value) representing the activity of glucose metabolism of the liver. (N = 8 mice). **i**, **j** (**i**) Representative gross images of liver pathology and hepatic Ki67 IHC staining of HGD mice treated with/without ST101. Scale bar, (top) 1 cm or (bottom) 100 μm. **j** Quantifications of liver/body weight ratio, tumor number, tumor size, and Ki67 signal intensity in the tumor samples. (N = 8 mice). Data are mean ± SD. *P* values were computed by unpaired t-test (two-sided) (**c**–**e**, **h**, **j**) or two-way ANOVA (two-sided) (**f**). NS non-significant. Source data are provided as a Source Data file.

Given the dominant role of C/EBPβ in hyperglycemia-driven HCC progression, we then investigated its therapeutic potential. ST101 (Lucicebtide) is a 38-amino acid D-peptide that competitively disrupts DNA binding, prevents dimerization, and induces proteasomal degradation of C/EBPβ (illustrated in Fig. 7a)^46^. This peptide is currently in Phase II trials for advanced solid tumors, although its in vivo antitumor activity and mechanism remain undefined. We confirmed that ST101 treatment rapidly triggered C/EBPβ degradation in HepG2 cells, concomitant with dose-dependent reductions in GLUT1, LDHA and HRAS expression (Fig. 7b). ST101 treatment also dose-dependently inhibited glycolysis, cancer cell survival and proliferation in HepG2 cells (Fig. 7c–f and Supplementary Fig. 10a). Next, we assessed therapeutic efficacy in vivo using hyperglycemia-induced HCC progression. ST101 (25 mg/kg) was administered three times weekly starting at 8 weeks of age. As expected, hepatocellular C/EBPβ protein levels were significantly reduced in ST101-treated HGD mice, compared with those with vehicle only (Fig. 7g). Concordantly, ST101 treatment decreased Glut1, Ldha, and Hras expression in HGD mice (Fig. 7g and Supplementary Fig. 10b). Remarkably, ST101-treated HGD mice exhibited drastically attenuated hepatocellular glycometabolism, HCC progression, and proliferation signal compared with vehicle-treated littermates (Fig. 7h-j). Hence, these data indicate ST101-mediated C/EBPβ blockade as a potential therapeutic strategy against hyperglycemia-accelerated HCC.

## Discussion

In this study, our data resolve longstanding questions regarding how glycometabolic disorders like diabetes and hyperglycemia accelerate tumorigenesis. We identify transcription factor C/EBPβ, specifically its LAP1 isoform, as a pivotal mediator through which hyperglycemia fuels HCC progression. Hyperglycemia-driven C/EBPβ-LAP1 activation orchestrates a dual oncogenic program: 1) transcriptional upregulation of glycolytic effectors (e.g., GLUT1, LDHA) to enhance glycolysis, and 2) direct induction of the oncogene *HRAS* to drive proliferation (illustrated in Supplementary Fig. 11). Importantly, we delineate that hyperglycemia activates C/EBPβ isoforms via the ROS-PERK-eIF2α-ATF4 axis. This work demonstrates C/EBPβ as a potential therapeutic target for glucose-accelerated malignancies, validated by C/EBPβ inhibitor Lucicebtide (ST101) in suppressing hyperglycemia-fueled HCC progression.

C/EBPβ exhibits oncogenic functions, as reported in various cell types and conditions. For instance, Liu et al.^16^ reported that C/EBPβ-dependent transcriptional networks mediate obesity-driven tumor initiation. This study^16^ identified LAP2, but not LIP, showed oncogenic properties by promoting tumor sphere formation and upregulating *CLDN1* and *LCN2* in both MDA-MB-231 and HCC1806 cells. In two TNBC mouse models, glycolysis restriction was shown to repress LAP2 expression specifically^47^. By overexpressing or depleting different C/EBPβ isoforms, they found that LAPs, particularly LAP2, regulate *G-CSF* and *GM-CSF* expression to induce tumor immunosuppression through the development of myeloid-derived suppressor cells (MDSCs)^47,48^. This work also highlights the connection between LAP isoforms and glycometabolism. Additionally, another study in TNBC48 utilized integrative sequencing analyses to demonstrate that LAP enhances chromatin accessibility and activates the EGFR signaling pathway, which critically promotes TNBC growth, whereas LIP has minimal effects on these phenotypes. Importantly, different groups reported that complete depletion of C/EBPβ increases expression of the well-known tumor suppressor p53 in mice, and all three isoforms interact to suppress p53 transcriptional activity^18,19^. LAP1 has also been shown to enhance the transcription of *NQO1* and *GSTP1*, facilitating glioblastoma proliferation^17^. Moreover, C/EBPβ has been implicated in driving defective autophagy and NK cell dysfunction in solid tumors, where its inhibition restores NK cell functions against tumors^49^. Phosphorylation of C/EBPβ on Thr217 has been found to enhance its association with procaspases 1 and 8, thereby inhibiting their activation and promoting the survival of hepatic stellate cells^50^.

There are also studies showing the oncogenic function of LIP specifically. In mouse embryonic fibroblasts (MEF), LIP overexpression has been reported to promote aerobic glycolysis and mitochondrial respiration by activating the RNA-binding protein LIN28B^51,52^. Genetic depletion of LIP in mice has been associated with a metabolic shift towards enhanced fat metabolism and improved insulin sensitivity, while reducing carbohydrate metabolism^36,53^. These mice also experience delayed hepatocyte regeneration and impaired osteoclast differentiation^54^. Furthermore, LIP overexpression in mice has been linked to increased cancer susceptibility, particularly for T-lymphoma, and carcinomas of the skin, liver, and mammary gland^55^. However, how glucose consumption varies across cell types and cancer stages requires careful interpretation of the physiological functions of LIP, particularly since many of these experiments were not conducted in well-established liver cancer models, failing to reflect tumorigenesis in hepatocytes under hyperglycemic conditions. In our study, we identified a correlation between LAP1 expression and glycometabolism (Fig. 1 and Supplementary Fig. 3). We then found that LAP1 enhances glycolysis and promotes cancer cell proliferation in HCC models. However, overexpression of LIP alone did not yield comparable effects in HepG2 cells or the xenograft model.

Although C/EBPβ has been implicated in cancer development and its antagonists are in clinical trials, their mechanisms remained poorly defined^15,46,56^. Lucicebtide (ST101) is a Phase II C/EBPβ-specific inhibitory peptide for solid tumors. However, its in vivo efficacy and mechanism are not fully examined. We demonstrated Lucicebtide suppressed glycolysis, Hras expression, and HCC progression in HGD-fed mice, disrupting both metabolic and oncogenic arms (Fig. 7). Our findings also extend beyond Lucicebtide to provide molecular rationale for other clinical-stage C/EBPβ inhibitors. Withaferin A (WFA) is currently ongoing a phase-I/II clinical trial to treat recurrent ovarian cancer with the combination of DOXIL^57,58^. One of its major downstream targets is C/EBPβ^59^. Helenalin acetate and its derivatives are C/EBPβ inhibitors, blocking cell proliferation and oncogenic activity of acute myeloid leukemia cells^60,61^. Conceivably, this study provides molecular insight into the anti-tumor activities of these C/EBPβ antagonists, repurposing them for hyperglycemia-fueled cancers. Complementary targeting of the inhibiting the upstream ROS-PERK-ATF4 axis (e.g., via mQ, TUDCA, ISRIB) also offers a strategic approach to overcome therapeutic resistance.

We demonstrated that Lucicebtide (ST101) could successfully inhibit all three C/EBPβ isoforms. Because it targets a conserved motif shared by all isoforms, it shows no isoform preference, consistent with our results (Fig. 7b, g and Supplementary Fig. 10b). Given that all three C/EBPβ isoforms have been reported to exert oncogenic functions through different mechanisms, this broad activity may offer an advantage in cancer treatment. However, for precision medicine—such as hyperglycemia-elicited HCC—an LAP isoform-specific inhibitor is urgently needed.

While glycolysis provides rapid energy and biosynthetic precursors, oncogenes amplify proliferative signaling during tumorigenesis. This study unravels C/EBPβ-LAP1 as a unique master regulator to govern both processes. Cancer cells predominantly rely on glycolysis over oxidative phosphorylation to fuel their progression (Warburg effect) by upregulating the glucose transporters (e.g., GLUT1), glycolytic enzymes, and oncoproteins^10^. However, the precise regulatory mechanisms are not fully understood^11,12^. Our findings provide mechanistic insight into why cancer cells favor glycolysis for energy production through C/EBPβ-mediated metabolic reprogramming. These findings also align with clinical observations of HRAS upregulation in diabetic patients and its correlation with poor HCC survival. Beyond LAP1-mediated HRAS regulation reported here (Fig. 4 and Supplementary Fig. 4), RAS and C/EBPβ are mechanistically intertwined: Ras-induced skin tumors require C/EBPβ for survival, while Ras activates C/EBPβ transcription and phosphorylation^62–64^. Collectively, these studies indicate a positive feedback loop between C/EBPβ and RAS to mutually activate each other, thereby rapidly accelerating and deteriorating cancer progression.

Prior studies linked C/EBPβ to glycometabolic dysfunction and ER stress activation; however, its upstream regulation remained uncharacterized^13,14^. In this study, we disclose the regulatory mechanism by establishing a ROS-PERK-eIF2α-ATF4 axis, which is required for C/EBPβ transcription upon high glucose stimulation (Fig. 2). Notably, other well-established nutrient-sensing regulators (e.g., mTOR, NF-κB) are dispensable for this process. This finding aligns with ER stress’s pathological roles in diabetes and cancer^65,66^. The reversibility of this axis by antioxidants (mQ), ER stress alleviators (TUDCA) or PERK-ATF4 signaling inhibitor (ISRIB) opens the avenues for co-targeting metabolic and proteostatic vulnerabilities in diabetic cancers.

In addition, glucose restriction can promote Integrated Stress Response (ISR) and eIF2α phosphorylation through mechanisms such as N-linked glycosylation inhibition in neuronal cells or MEF^67,68^. Diabetes or hyperglycemia can also induce eIF2α phosphorylation by eliciting ER stress^69^. Moreover, several studies, together with this study, have demonstrated that high glucose levels are correlated with UPR (unfolded protein response) activation and eIF2α phosphorylation. Increased phosphorylation of eIF2α is often associated with cellular stress, including ER stress, which can be triggered by high glucose levels and contribute to the development of ISR^70,71^. Thus, it appears that both high glucose and glucose deprivation can potentially promote ISR and eIF2α phosphorylation, albeit through different mechanisms. Interestingly, this possibly partially explain how the progression of hepatocellular carcinoma (HCC) can be promoted by either hyperglycemia or hypoglycemia^2^, while normal blood glucose levels are associated with the lowest incidence of cancer.

Phosphorylation of eIF2α decreases the translation of LIP, shifting the balance toward the longer LAP1 and LAP2 isoforms, while unphosphorylated eIF2α facilitates LIP expression^72,73^. Additionally, calorie restriction (CR) has been shown to decrease LIP expression via mTOR inhibition^36^. Furthermore, the work by David Ron’s lab^69^ also demonstrated that ISR enhances the translation of C/EBPβ-LAP isoforms. In our study on hyperglycemia-induced HCC progression, we observed that high glucose indeed elicits ER stress and upregulates C/EBPβ at both protein and mRNA levels. By knocking down various key UPR-responsive elements, we determined that the PERK-eIF2α-ATF4 axis is required for glucose-induced upregulation of C/EBPβ mRNA, resulting in increased levels of all three isoforms. Intriguingly, while C/EBPβ mRNA levels were comparable between tumor and non-tumor tissues in HCC patients (Supplementary Fig. 1c), C/EBPβ protein isoforms were significantly elevated in tumors. Presumably, these mRNA and protein expression differences indicate that there are other upstream regulations of C/EBPβ in tumorigenesis, potentially at the post-translational level, awaiting further investigation.

We demonstrate that C/EBPβ-LAP isoforms, but not LIP, correlated with fasting glucose levels in HCC patients. The functional difference between LAPs (activators) and LIP (repressor) isoforms is worth noting, as only LAPs promote glycolysis and cell proliferation via GLUT1, LDHA, and HRAS. The N-terminally truncated isoform LIP exerts an inhibitory function due to its lack of transactivation domains while retaining DNA-binding capability, enabling it to compete with the transcriptional activators LAP1 and LAP2^20^. Previous studies have demonstrated that LIP expression is upregulated by mTORC1 signaling through a *cis*-regulatory short upstream open reading frame (uORF) within the *Cebpb* mRNA^36,73^. Chronic obesity is associated with severe metabolic disorders and an elevated risk of cancer development. Intriguingly, genetic ablation of the inhibitory isoform C/EBPβ-LIP enhances the activity of the transactivating isoform C/EBPβ-LAP, promoting lipid metabolism under normocaloric conditions and extending both healthspan and lifespan in mice. Under high-fat diet conditions, LIP deficiency induces adipocyte hyperplasia, concomitant with reduced inflammation and improved metabolic parameters. Moreover, male LIP-deficient mice exhibit a pronounced increase in subcutaneous fat storage^74^. Together, these findings establish C/EBPβ as a critical regulator of adipocyte plasticity in response to excessive dietary fat, with significant implications for metabolic health and aging. Conceivably, this specificity explains context-dependent outcomes of global C/EBPβ manipulation and underscores the necessity for isoform-specific targeted therapies. There is also an important difference between LAP1 and LAP2. The extreme N-terminus, which is unique to LAP1 and absent from the other C/EBPβ isoforms, is required for recruitment of the SWI/SNF chromatin remodeling complex^75^. Therefore, in our work, we performed a full spectrum experiment comparing the function of LAP1/LAP2/LIP in glycolysis, proliferation and gene regulation in HepG2 cells (Fig. 3), the results clearly show that both LAP1 and LAP2, but not LIP, increase glycolytic flux, cell proliferation, and relevant gene upregulation (GLUT1, LHDA, etc). These results suggest that both LAPs, independent of SWI/SNF chromatin remodeling complex, promote HCC progression by upregulating glycolysis and proliferation.

Aging is marked by a gradual decline in physiological integrity, resulting in diminished functional capacity and heightened susceptibility to mortality. This degenerative process serves as a principal risk factor for numerous major human pathologies, such as cancer, diabetes, cardiovascular disorders, and neurodegenerative diseases^76^. Mounting evidence supports that C/EBPβ acts as a major aging driver^77–81^. Recently, we and other groups in the field, have reported that C/EBPβ is implicated in age-related disorders including neurodegenerative diseases, diabetes and atherosclerosis^82–85^. Consequently, these lines of evidence indicate that C/EBPβ may play an essential role in eliciting age-related disorders from neurodegenerative diseases to diabetes, atherosclerosis and cancers^84,86^.

There are also limitations in this study. In the current work, our study focuses on HCC models, with validation in colon cancer cells (given their strong epidemiological connections to diabetes). Certainly, C/EBPβ’s roles in other popular cancers types (e.g., lung, breast, etc.) warrants further investigation. For the in vivo experiments, we used a Rosa26-LSL-LAP1 mice to conditionally express LAP1 in hepatocytes. The isoforms specific roles of C/EBPβ require other in vivo models. Thus, we reach a cautious conclusion that C/EBPβ isoform LAP1 is a critical regulator and potential therapeutic target for glucose-fueled glycolysis, cell proliferation and cancer progression.

Collectively, our study represents a fundamental advance in understanding of hyperglycemia-cancer link by establishing C/EBPβ-LAP1 as a master transcriptional regulator of metabolic and proliferative reprogramming. C/EBPβ-LAPs is activated by hyperglycemia though a ROS-PERK-ATF4 signaling cascade, driving tumor progression through coordinated upregulation of key glycolytic effectors (e.g., GLUT1, LDHA) and *HRAS* oncogene (illustrated in Supplementary Fig. 11). This pathway provides a mechanistic basis for the accelerated cancer incidence observed in diabetic patients and identifies C/EBPβ as a potential therapeutic target in precision oncology. Given the global rise in metabolic disorders and cancer incidences, our findings offer timely and rational strategies to break pathogenic link between hyperglycemia and cancer.

## Methods

This research complies with all relevant ethical regulations. Human tissue studies were approved by the Institutional Review Boards of Xinhua Hospital Affiliated to Shanghai Jiao Tong University and the First Affiliated Hospital of Guangxi Medical University. Animal and other studies were approved by the Shenzhen Institutes of Advanced Technology.

### Cell culture

Human HepG2, HCT116, and HEK293 cells were cultured at 37 °C under 5% CO₂ in Dulbecco’s Modified Eagle Medium (DMEM; Gibco), supplemented with 10% fetal bovine serum (FBS; Gibco) and 1% penicillin/streptomycin (Gibco). Glucose concentrations were adjusted as specified. CEBPB-knockout HepG2 cells were generated by Cyagen. Lentiviruses to generate stable cell lines were produced by Obio Technology (Shanghai) and BrainVTA (Wuhan), following the manufacturers’ protocols. All stably transfected cells were selected by puromycin for 7 days.

### Animal studies

All mice were housed under specific pathogen-free conditions with a 12-hour light/dark cycle (lights on from 7:00 AM to 7:00 PM). Ambient temperature was maintained at 22 ± 2 °C, and relative humidity was kept at 40–60%, ensuring optimal health and welfare throughout the study. Protocols were approved by the Shenzhen Institutes of Advanced Technology (SIAT-IACUC-240130-SLG-YKQ-A2465). Nude mice (BALB/c-nu/nu) for xenografts were purchased from Cyagen. Albumin-CreERT2, C/ebpβ-flox, and Rosa26-LSL-LAP1 mice (conditionally expressing full-length human C/EBPβ, C57BL/6 J background) were purchansed from Cyagen and genotyped using provider-specified primers (listed below). The Albumin-CreERT2 and Rosa26-LSL-LAP1 alleles were kept in heterozygous. CreERT2 nuclear translocation in vivo was achieved by intraperitoneal (IP) injection of 80 mg/kg tamoxifen (Sigma-Aldrich) on 5 consecutive days. Only male mice were studied, reflecting hepatocellular carcinoma gender prevalence. Mice were fed ad libitum with normal chow diet (NCD, 3.44 kcal/g, composition: 24% Protein, 13% Fat, and 63% Carbohydrate). For High–glucose diet studies, mice were fed ad libitum with normal chow diet or high–glucose diet (Topbiotech) starting at 2-month of age, which has the same caloric content as normal control diet (3.79 kcal/g), but with 20% glucose replacing corresponding amount of cornmeal for carbohydrate.

The following genotyping primers were used: C/ebpβ-flox 5’-GCCCTCTCGCGCTCCCGTGGCCG-3’, 5’-GTCGGGCTCGTAGTAGAAGTTG-3’ (284 bp for WT, 352 bp for floxed); Rosa26-LSL-LAP1 5’-CACAGCGACGAGTACAAGATCC-3’, 5’-AAAGGCATTAAAGCAGCGTATCC-3’ (341 bp for mutant); Albumin-CreERT2 5’-TGCAAACATCACATGCACAC-3’, 5’-TTGGCCCCTTACCATAACTG-3’, 5’-GAAGCAGAAGCTTAGGAAGATGG-3’ (390 bp for mutant and 351 bp for WT).

### Human tissues

hepatocellular carcinoma tissues and clinical data were obtained from Xinhua Hospital Affiliated to Shanghai Jiao Tong University and the First Affiliated Hospital of Guangxi Medical University. Tissues were immediately snap frozen in liquid nitrogen. Ethics approval was granted by the Institutional Review Board of Xinhua Hospital Affiliated to Shanghai Jiao Tong University (XHECC-2022-104-1) and the First Affiliated Hospital of Guangxi Medical University (2025-S0745). Patient information is listed in Supplementary Tables 1–3. Informed consent was obtained from the patients both for the use of their samples and also to publish their information.

### Glucose uptake and lactate production

Cells seeded in 96-well plates were washed once with PBS and incubated with 50 µL 1 mM 2-deoxyglucose (2-DG) for 10 min. Glucose uptake was quantified using the Glucose Uptake-Glo Assay (Promega, J1341). For lactate measurement, cells were washed with PBS and incubated in phenol red-free complete medium for 1 h. Supernatants were analyzed with the Lactate Colorimetric/Fluorometric Assay Kit (BioVision, K607-100). Both assays followed manufacturers’ protocols.

### Extracellular acidification rate (ECAR)

ECAR was measured using a Seahorse XF96 Analyzer (Seahorse Bioscience). Cells (2 × 10⁴/well) were seeded in XF96 plates 24 h before the assay. After washing with XF assay medium (XF base medium with 4 mM L-glutamine, pH 7.4), cells were equilibrated for 1 h at 37 °C in a non-CO₂ incubator. Sensor cartridges were loaded with glucose (100 mM), oligomycin (10 µM), and 2-DG (500 mM) into ports A, B, and C, respectively. Data were analyzed using Seahorse WAVE and GraphPad Prism.

### Colony formation assay

Cells (2 × 10³) were plated in 12-well plates for 12 days. Colonies were PBS-rinsed, fixed with methanol, stained with 0.5% crystal violet, and dissolved in 2% SDS. Absorbance at 600 nm determined relative colony formation, which indicates tumor survival.

### Cell proliferation assay

Cells were counted and seeded in 12-well plates. Viable cells were counted every 12 h.

### Plasmid DNA and siRNA transfection

Plasmids (SynBio Technologies) and siRNAs (RIBOBIO) were transfected using Lipofectamine 3000 (Thermo Fisher Scientific) following manufacturer’s guidelines.

### Xenograft tumorigenesis assay

HepG2 cells (1 × 10⁶) were subcutaneously injected into athymic nude mice. Tumor dimensions were measured using digital calipers, with volumes calculated as V = (Length × Width²)/2. Tumor growth was monitored regularly throughout the experiment. The maximal tumor burden (1.5 cm in diameter or 1500 mm³ or 10% of body weight) permitted by the institutional animal ethics committee was not exceeded, and animals were euthanized before tumors reached any approved humane endpoint. Tumor progression was assessed based on the measurement of tumor volume. Mice were monitored daily for signs of distress, and tumor dimensions were recorded weekly. The experimental endpoint was determined by the growth of tumors reaching the maximal limit permitted by our ethics committee, or by the appearance of any clinically relevant signs of discomfort, including significant weight loss, lethargy, or difficulty in mobility.

### ST101 administration

ST101 (H₂N-vaeareelerlearlgqargelkkwkmrrnqfwlklqr-OH; synthesized by QYAOBIO) was administered at 5–20 mM for 24 h in vitro. For in vivo studies, mice received intravenous ST101 (25 mg/kg) three times weekly from age of 8 weeks until endpoint.

### AAV8 delivery

Adeno-associated virus serotype 8 (AAV8) expressing TBG promoter-driven shHRAS or control (Obio Technology) was injected via tail vein (2 × 10¹¹ vector genomes in 100 μL).

### DEN/CCl₄ hepatocellular carcinoma model

Male mice received intraperitoneal N-nitrosodiethylamine (DEN; 1 mg/kg) at postnatal day 14, followed by twice-weekly carbon tetrachloride (CCl₄; 0.2 mL/kg i.p.). 100% of male mice developed hepatocellular carcinoma by 22 weeks. The assessment of disease progression in this model was conducted by monitoring the development of clinical signs of disease, including weight loss, changes in grooming behavior, and overall activity levels. Humane endpoints were established, and experiments were concluded if any mouse exhibited significant weight loss ( > 20% of baseline weight) or other distressing symptoms. All mice were sacrificed at the end of the 22-week study period.

### STZ-induced Type1 DM mice

To establish a type 1 diabetes mellitus (T1DM) mouse model, mice were fasted for 4–6 h before intraperitoneal injection of streptozotocin (STZ; freshly prepared in cold citrate buffer, pH 4.5) at a dose of 50 mg/kg body weight once daily for 5 consecutive days. Mice with persistent hyperglycemia (typically blood glucose > 16.7 mmol/L for at least two consecutive measurements) were considered diabetic and included in subsequent analyses.

### ^18^F-FDG microPET scan in mice

After 3 h fasting, mice received tail vein injections of ¹⁸F-FDG (9.25 MBq/10 g; radiochemical purity >95%) in ~100 μL of saline. Following 30-min distribution, mice were weighed and subjected to a high-resolution small animal PET scanning device (microPET) with a spatial resolution of 1.0 mm. Inhalation anesthesia with isoflurane was performed throughout the experiments. Emission scans were acquired in volumetric mode for the 20 minutes and the recorded PET images were reconstructed by using ordered-subset expectation maximization (OSEM) with 16 subsets and 5 iterations. The statistical results of standard uptake value (SUV) in the region of interest (ROI) were calculated and analyzed by Amide 1.0.4-1 (San Diego, CA 92101).

### RNA sequencing

RNA was extracted from biological triplicates/quadruplicates by TRIzol reagent (ThermoFisher, 15596026). Libraries were sequenced, and reads aligned to the human genome (HISAT2). Gene expression (TPM) and differential analysis (DESeq2) were performed, with pathway enrichment (positive regulators of glycolysis) assessed by GSEA (clusterProfiler) and heatmaps created by pheatmap.

### Quantitative PCR

Total RNA (TRIzol-extracted) was DNase-treated and reverse-transcribed (HiScript IV RT SuperMix, Vazyme). qPCR used performed using SYBR Premix Ex Taq (TaKaRa) on an Applied Biosystems StepOnePlus TM Real-Time PCR instrument, with relative expression (ΔΔCt) normalized to ACTIN. The primers used in this study are listed in Supplemental Table 4.

### Immunoblotting

Tissues were lysed in RIPA (Beyotime, P0013B, with protease inhibitors) at 4 °C Cells were lysed in lysis buffer (50 mM Tris pH 7.4, 40 mM NaCl, 1 mM EDTA, 0.5% Triton X-100, protease inhibitors) and centrifuged (24,000 × *g*, 15 min). Proteins were separated by SDS-PAGE, transferred to PVDF membrane (Millipore), blocked in 5% milk in TBST, and applied with primary antibodies (4 °C overnight), followed by secondary antibodies (1 h RT). Signals were visualized by enhanced chemiluminescence according to the manufacturer’s instructions (Bio-Rad). Antibodies used in this study are listed in Supplemental Table 5. Vinculin and Tubulin were used as housekeeping proteins in our Western blots. Both of these proteins are commonly utilized as loading controls in the field because they are expected to maintain stable expression under our experimental conditions.

For Fig. 1a and Supplementary Fig. 1a, C/EBPβ and VINCULIN were obtained from the same blot; IL-6 and BIP were obtained from the same blot; ATF4 and HRAS were obtained from the same blot; TNFα and GLUT1 were obtained from the same blot. For Fig. 1f, C/EBPβ and VINCULIN were obtained from the same blot; Bip and Atf4 were obtained from the same blot. For Supplementary Fig. 1e, C/EBPβ and VINCULIN were obtained from the same blot; Bip and Atf4 were obtained from the same blot. For Supplementary Fig. 2a, C/EBPβ and VINCULIN were obtained from the same blot. For Supplementary Fig. 5, C/EBPβ and TUBULIN were obtained from the same blot. For Fig. 2 and Supplementary Fig. 6, C/EBPβ and VINCULIN/TUBULIN were obtained from the same blot. For Fig. 4e, f, C/EBPβ and VINCULIN were obtained from the same blot; GLUT1 and HRAS were obtained from the same blot. For Fig. 4h and Supplementary Fig. 7b, C/EBPβ and TUBULIN were obtained from the same blot; GLUT1 and HRAS were obtained from the same blot. For Fig. 5h and Fig. 6c, C/EBPβ and TUBULIN were obtained from the same blot; GLUT1 and HRAS were obtained from the same blot; TNFα and Cd68 were obtained from the same blot. For Fig. 7b, g, C/EBPβ and TUBULIN were obtained from the same blot; GLUT1 and HRAS were obtained from the same blot. Otherwise, the immunoblotting images were obtained from different blots. The samples derive from the same experiment, but different gels were processed in parallel. uncropped and unprocessed scans of all blots were provided in the Source Data file.

### Immunofluorescence and confocal microscopy

Cells grown on coverslips were fixed with 4% paraformaldehyde (20 min), permeabilized with 0.4% Triton X-100 (10 min), and blocked with 5% BSA (1 h). Primary antibodies were incubated overnight at 4 °C. After PBS washes, samples were incubated with secondary antibodies (1:2000, 1 h RT) and/or Proteostat (1:20,000, 30 min), followed by extensive PBS washing. Coverslips were mounted using DAPI-containing medium (Beyotime).

For ROS detection, cells were incubated with 25 µM H₂DCFDA (MCE, HY-D0940) in PBS (30 min, 37 °C, 5% CO₂). Fluorescence was quantified at 488 nm excitation (BioTek Synergy H1) and normalized to protein concentration (BCA assay, Beyotime P0009). Images were acquired using a Zeiss LSM 980 confocal microscope with z-stack acquisition and processed with ZEN/Fiji software.

### Immunohistochemistry

Free-floating 12-μm aortic sections were incubated in 0.3% H₂O₂ (10 min) to quench endogenous peroxidases, followed by three PBS washes. Sections were blocked in 1% BSA/0.3% Triton X-100 (30 min), then incubated overnight at 4 °C with anti-Ki67 (Abcam, ab15580, 1:500). Signals were developed using an HRP/DAB detection kit (Abcam).

### Luciferase reporter assay

Human HRAS promoter (region −2000 to +100 bp, related to TSS), mouse Hras promoter (region −2000 to +100 bp, related to TSS), human GLUT1, LDHA, or PFKL promoter (region −2000 to +100 bp, related to TSS) were cloned into pGL3-basic plasmid (SynBio Technologies). Luciferase activity was measured using the Luciferase Assay System (Promega, E1500) and normalized to protein concentration (BCA). Mutant promoters were indicated in the related figures.

### Chromatin immunoprecipitation (ChIP)

Cells were cross-linked with 1% formaldehyde (10 min, 37 °C). ChIP was performed using the ChIP Kit (Abcam, ab500) with C/EBPβ antibody (Santa Cruz, sc-7962 H-7). Binding sites were predicted by JASPAR. Immunoprecipitated DNA was recovered by phenol-chloroform extraction and analyzed by PCR. Regions approximately at +50 to −50 bp around binding sites were used as templates for PCR primer design, and the PCR primers are listed in Supplementary Table 4.

### Statistics & reproducibility

Quantitative analyses are detailed in Methods and figure legends, with sample sizes (n) explicitly indicated in figures. Statistical analyses were performed using GraphPad Prism 10.0, with specific tests (e.g., two-sided unpaired t-test, ANOVA) defined per experiment in figure legends. All data are presented as mean ± standard deviation (SD). No statistical method was used to predetermine sample size. No data points were excluded, and no formal outlier analysis was conducted. Subjects (e.g., mice) were randomly assigned to either the treatment group or the control group, when appropriate. Experimenters were blinded to experimental conditions during data acquisition and quantification where feasible.

### Reporting summary

Further information on research design is available in the Nature Portfolio Reporting Summary linked to this article.

## Supplementary information

Supplementary Information

Reporting Summary

Transparent Peer Review file

## Source data

Source Data

## Data Availability

All data in this study are available within the paper. The Sequencing data (RNA-seq) generated in this study have been deposited in the ArrayExpress database under accession codes E-MTAB-16207 and E-MTAB-16228. Source data are provided with this paper.
