# Supplementary material for: The transcription factor C/EBPβ promotes hyperglycemia-elicited glycolysis and liver cancer progression
Source: Nat Commun. 2026 Aug 1;17:9707. doi: 10.1038/s41467-026-76209-9 (PMC13562552; doi:10.1038/s41467-026-76209-9)
Supplement: Supplementary file 1 [file 41467_2026_76209_MOESM1_ESM.pdf]

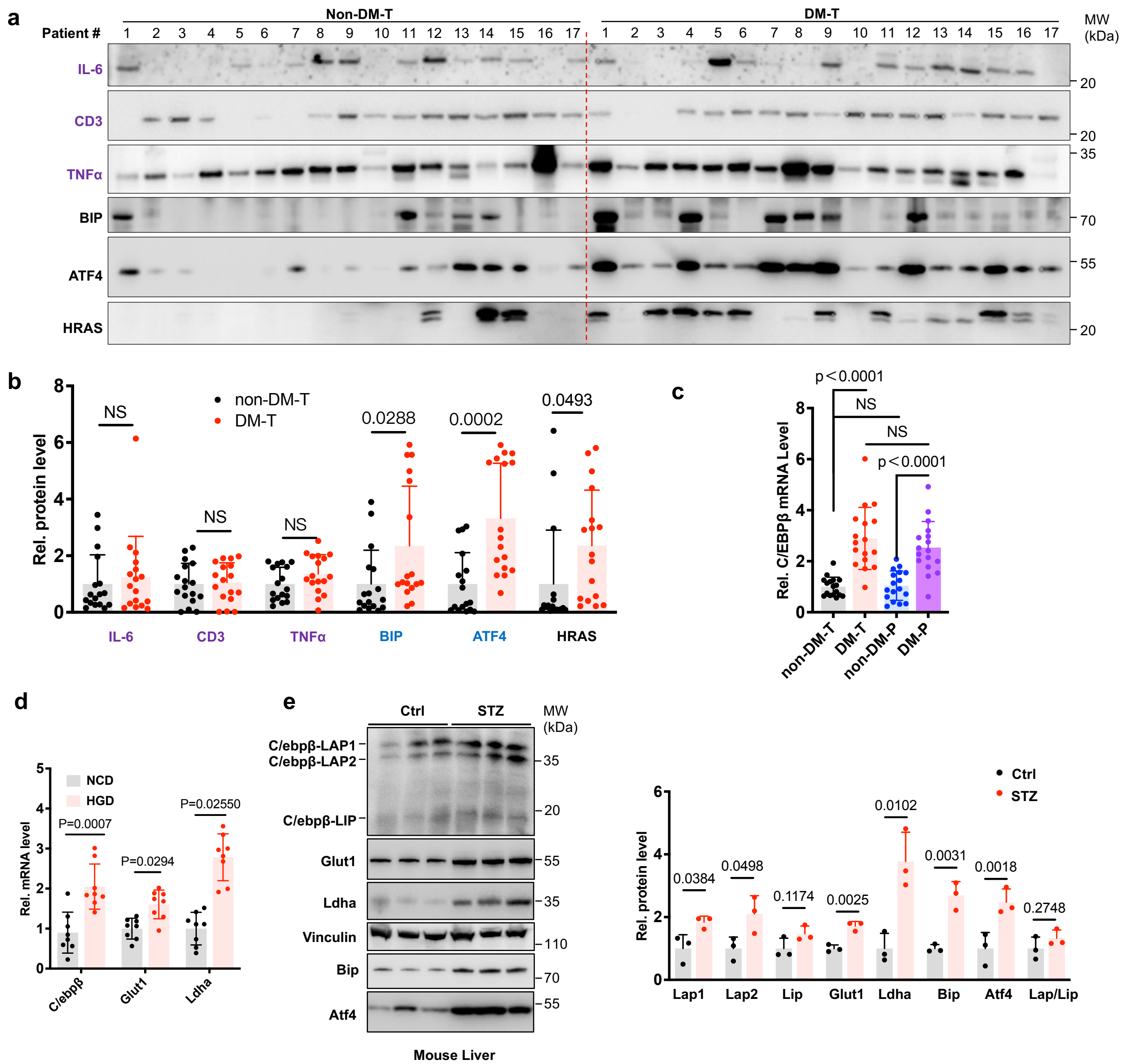

Supplementary Figure 1 | Increased hepatic C/EBPβ expression in HCC patients with DM.

a, **b,** (related to Fig. 1a) Immunoblotting **(a)** and Quantification **(b)** in tumor (T) from Hepatocellular Carcinoma (HCC) patients with or without Diabetes mellitus (DM). (N = 17 patients)

c, Relative C/EBPβ mRNA expression in tumor and paracancerous tissue from HCC patients with or without DM. (N = 17 patients)

d, Relative C/EBPβ, Glut1 and LDHA mRNA expression in mouse liver treated by NCD (normal chow diet) or HGD (High glucose diet). (N = 8 mice)

e, Immunoblotting (left) and Quantification (right) in liver samples from STZ-induced type 1 DM mice and control mice. (N = 3 mice)

Data are mean ± SD. P values were computed by unpaired *t* test (two-sided). NS, non-significant.

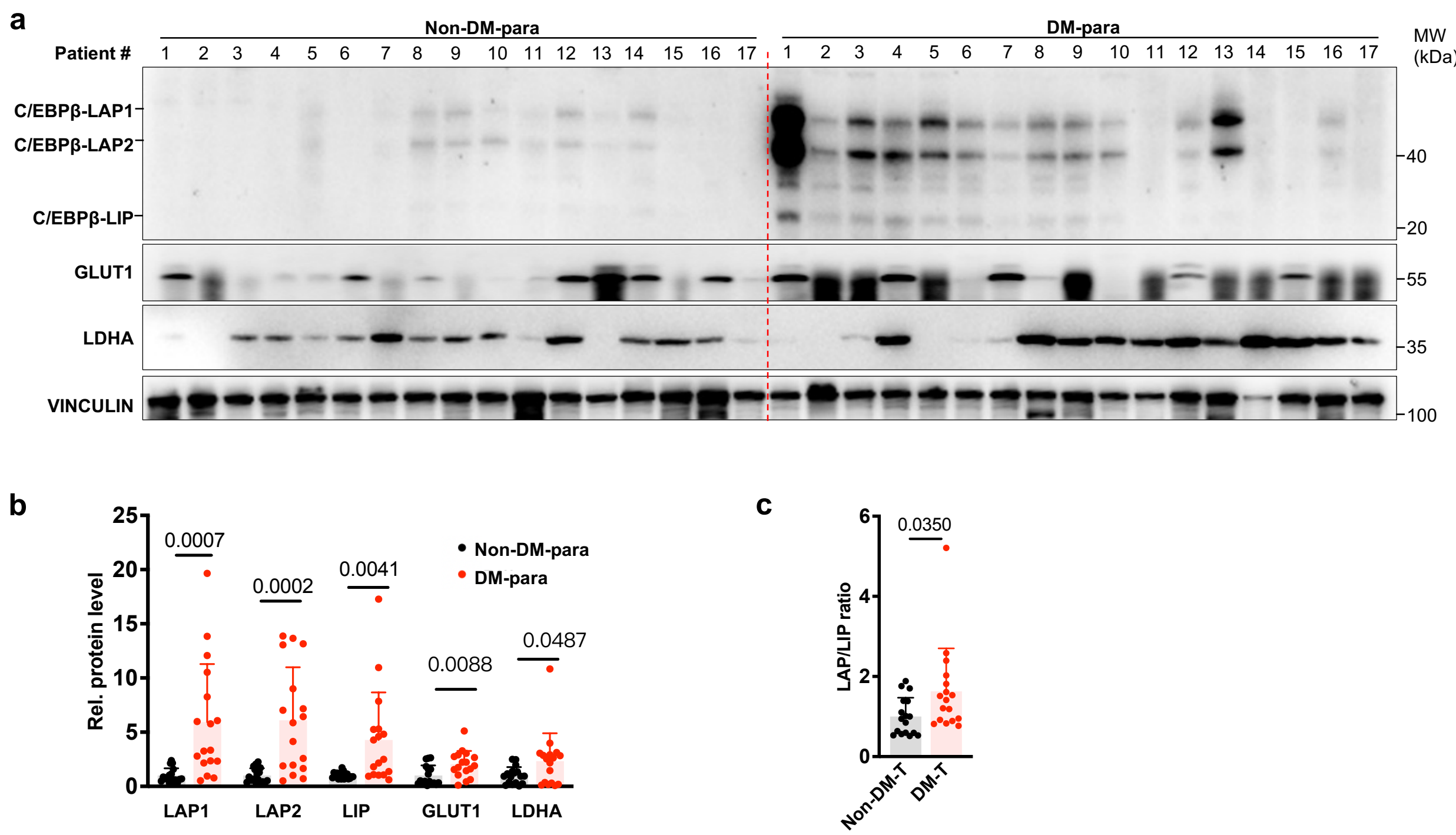

Supplementary Figure 2 | Increased hepatic C/EBPβ expression in HCC patients with DM.

a, **b,** Immunoblotting **(a)** and Quantification **(b)** in paracancerous tissue (Para) from Hepatocellular Carcinoma (HCC) patients with or without Diabetes mellitus (DM). (N = 17 patients)

c, The LAP/LIP ratio in paracancerous tissue from HCC patients with or without DM. LAP/LIP ratio was calculated by adding LAP1 and LAP2 and dividing this value by LIP. (N = 17 patients)

Data are mean ± SD. P values were computed by unpaired *t* test (two-sided).

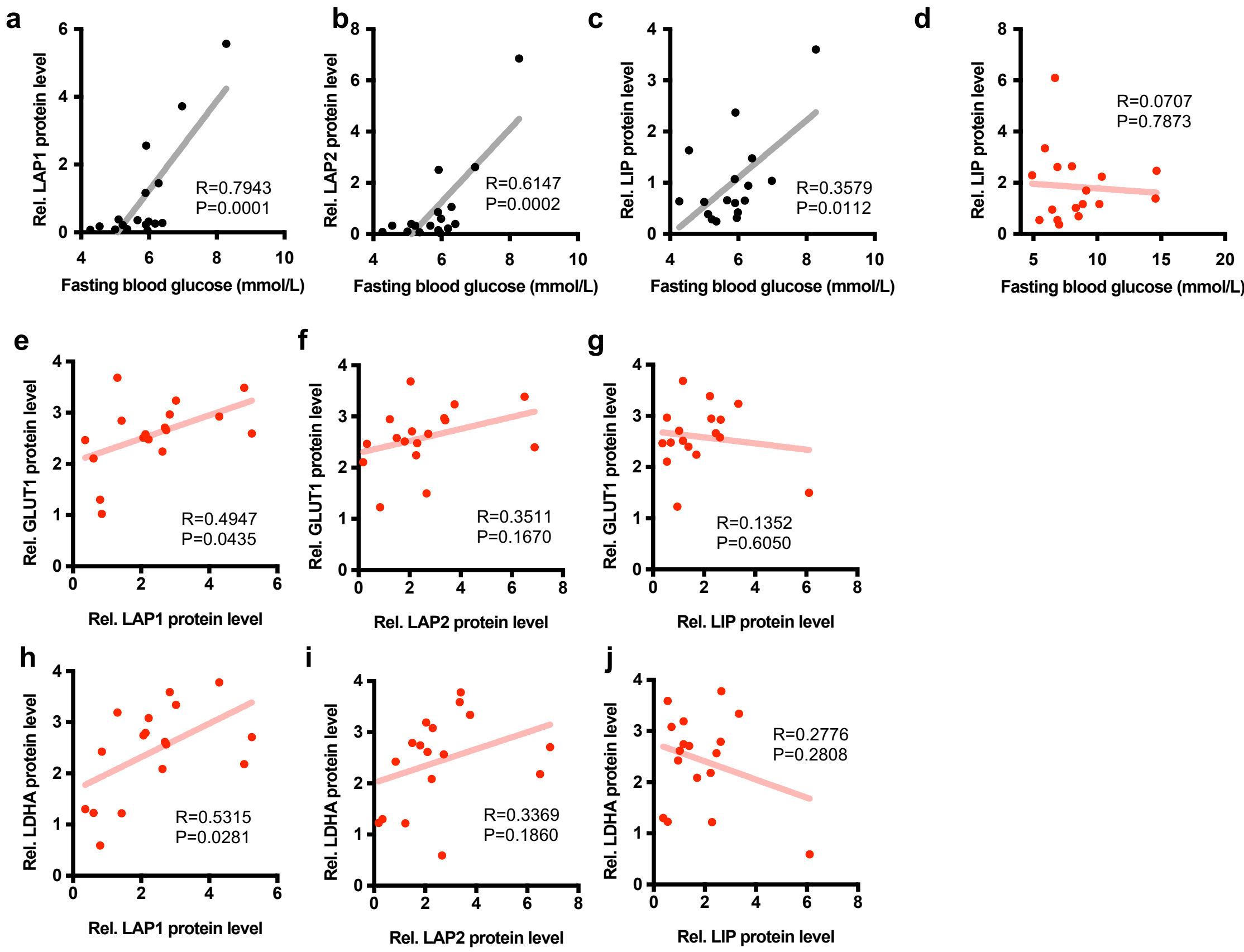

Supplementary Figure 3 | Correlation analysis of C/EBPβ isoforms in HCC patients.

a-c, Correlation analysis of FPG levels and C/EBPβ-LAP1 **(a)**, LAP2 **(b)**, and LIP **(c)** levels of paracancerous tissue in HCC patients. (N = 17 patients)

d, Correlation analysis of FPG levels and C/EBPβ-LIP levels of tumor tissue in HCC patients. (N = 17 patients)

e-j, Correlation analysis of C/EBPβ-isoforms levels and GLUT1 levels **(e-g)**/ LDHA levels **(h-j)** of tumor tissues in HCC patients. (N = 17 patients)

The regression slope (center line), the correlation coefficient (R) and P value are shown in the graph. P values were computed by unpaired *t* test (two-sided).

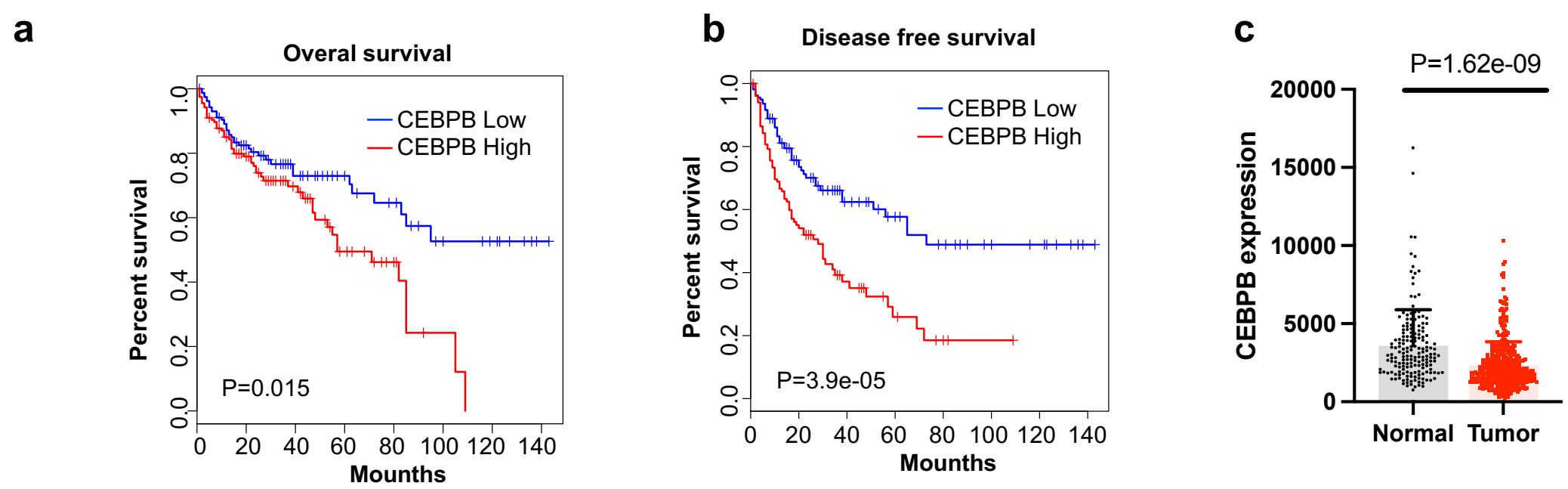

Supplementary Figure 4 | C/EBPβ is associated with worse survival in HCC and COAD.

a, Kaplan-Meier curve showing overall survival percentages of high and low CEBPB (cutoff: quartile) expression among HCC and COAD patients in the TCGA and the GTEx projects. The graph was created by GEPIA. HCC, Hepatocellular Carcinoma. COAD, Colon adenocarcinoma. (N=159 patients)

b, Kaplan-Meier curve showing disease free survival percentages of high and low CEBPB (cutoff: quartile) expression among HCC and COAD patients in the TCGA and the GTEx projects. The graph was created by GEPIA. (N=159 patients)

c, CEBPB mRNA expression in the liver samples from non-cancerous patients (N=225) and HCC patients (N=371). Data from TNMplot, which includes samples from GEO, GTEx, TCGA, and TARGET repositories.

P values were computed by two-sided log-rank test (a, b) or two-sided unpaired *t* test (c).

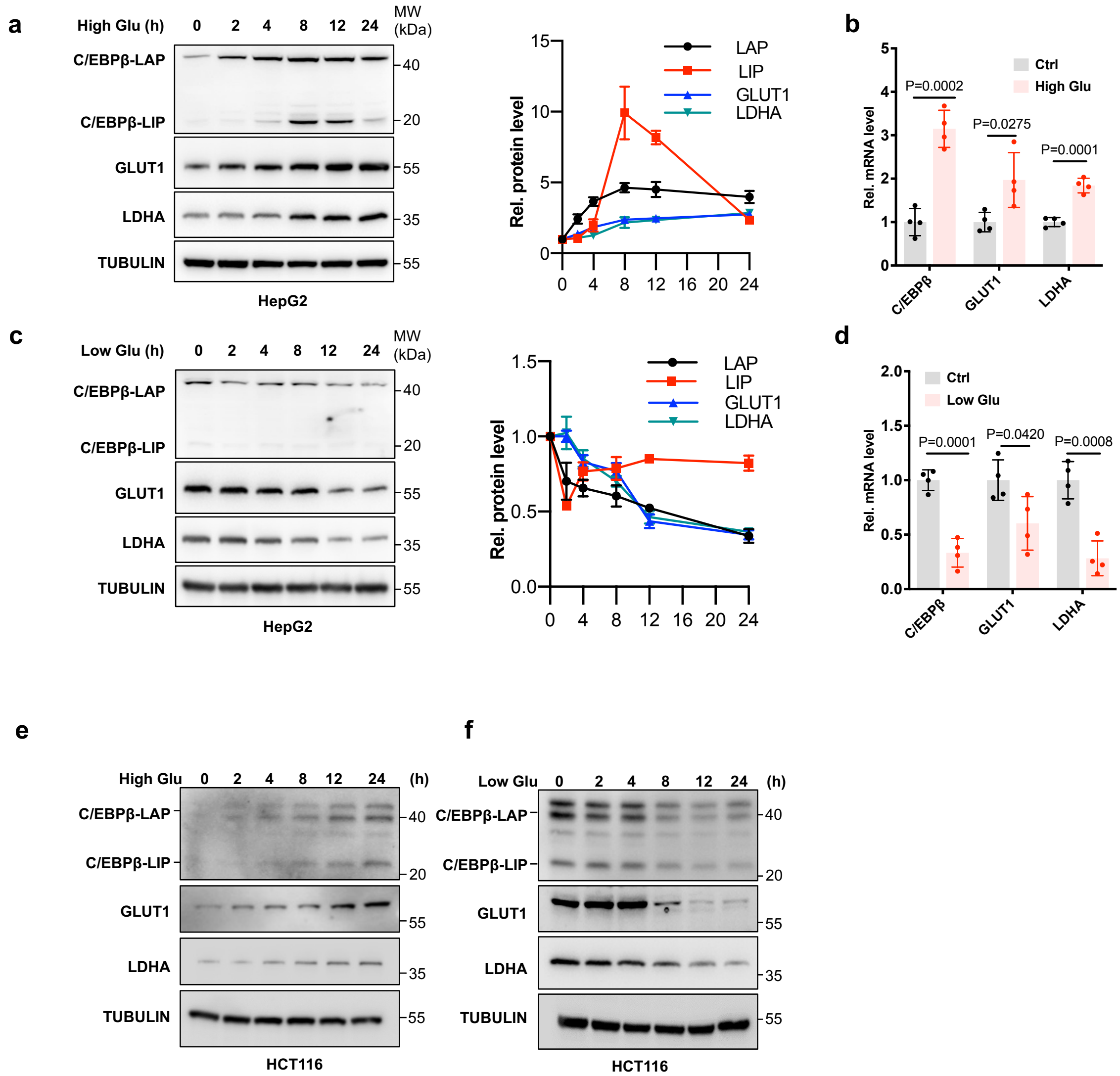

Supplementary Figure 5 | High glucose activates the expression of C/EBPβ LAPs in cancer cell lines.

a,b, Immunoblotting **(a)** and mRNA expression **(b)** in HepG2 cells treated with high glucose (12.8 mM) at the indicated time. (N = 4 independent experiments)

c,d, Immunoblotting **(c)** and mRNA expression **(d)** in HepG2 cells starved with low glucose (2.8 mM) at the indicated time. (N = 6 independent experiments)

e, Immunoblotting in HCT116 cells treated with high glucose (12.8 mM) at the indicated time.

f, Immunoblotting in HCT116 cells starved with low glucose (2.8 mM) at the indicated time.

Data are mean ± SD. P values were computed by two-sided unpaired *t* test.

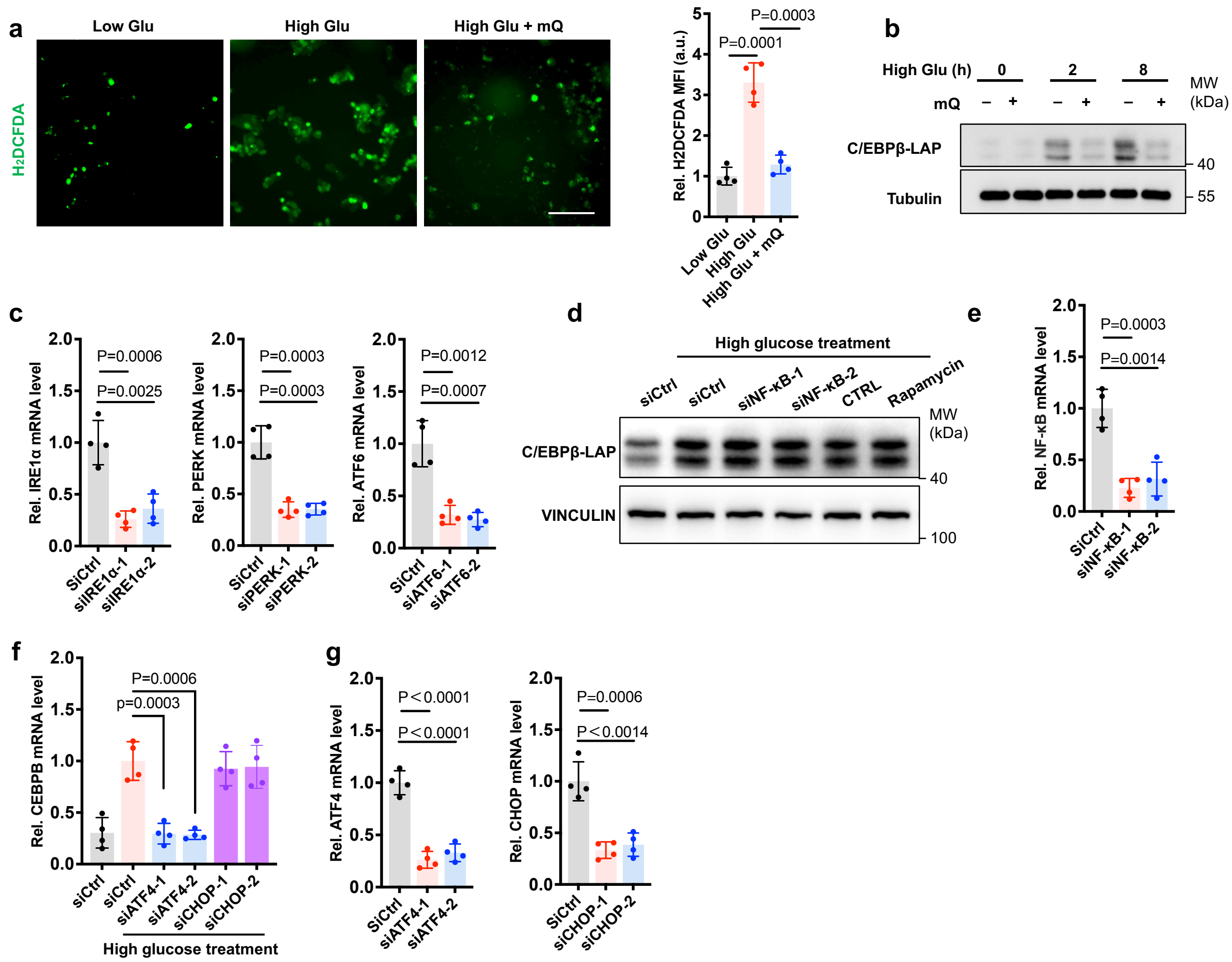

Supplementary Figure 6 | Glucose stimulates C/EBPβ transcription via PERK-eIF2α-ATF4 axis.

a, Representative H_2DCFDA staining images (left) and quantification (right) of Low Glucose, High glucose and High glucose + mQ treated HepG2 cells (N = 4 independent experiments). Scale bar, 1 mm.

b, Immunoblotting in high glucose treated (0, 2 and 8 h) HepG2 cells, with/without mQ.

c, Relative IRE1α, PERK and ATF6 mRNA levels in siCtrl, siIRE1α, siPERK and siATF6 HepG2 cells, with/without high glucose treatment. (N = 4 independent experiments)

d, Immunoblotting in siCtrl, siNF-κB, ctrl and rampamycin treated HepG2 cells, with/without high glucose treatment (12.8 mM, 8 h).

e, Relative NF-κB mRNA levels in siCtrl, siNF-κB-1 and siNF-κB-2 treated HepG2 cells (N = 4 independent experiments).

f, Relative CEBPB mRNA levels in siCtrl, siATF4 and siCHOP HepG2 cells, with/without high glucose treatment (12.8 mM, 8 h) (N = 4 independent experiments).

g, Relative ATF4 and CHOP mRNA levels in siCtrl, siATF4 and siCHOP treated HepG2 cells, with/without high glucose treatment (N = 4 independent experiments).

Data are mean ± SD. P values were computed by two-sided unpaired *t* test. NS, non-significant.

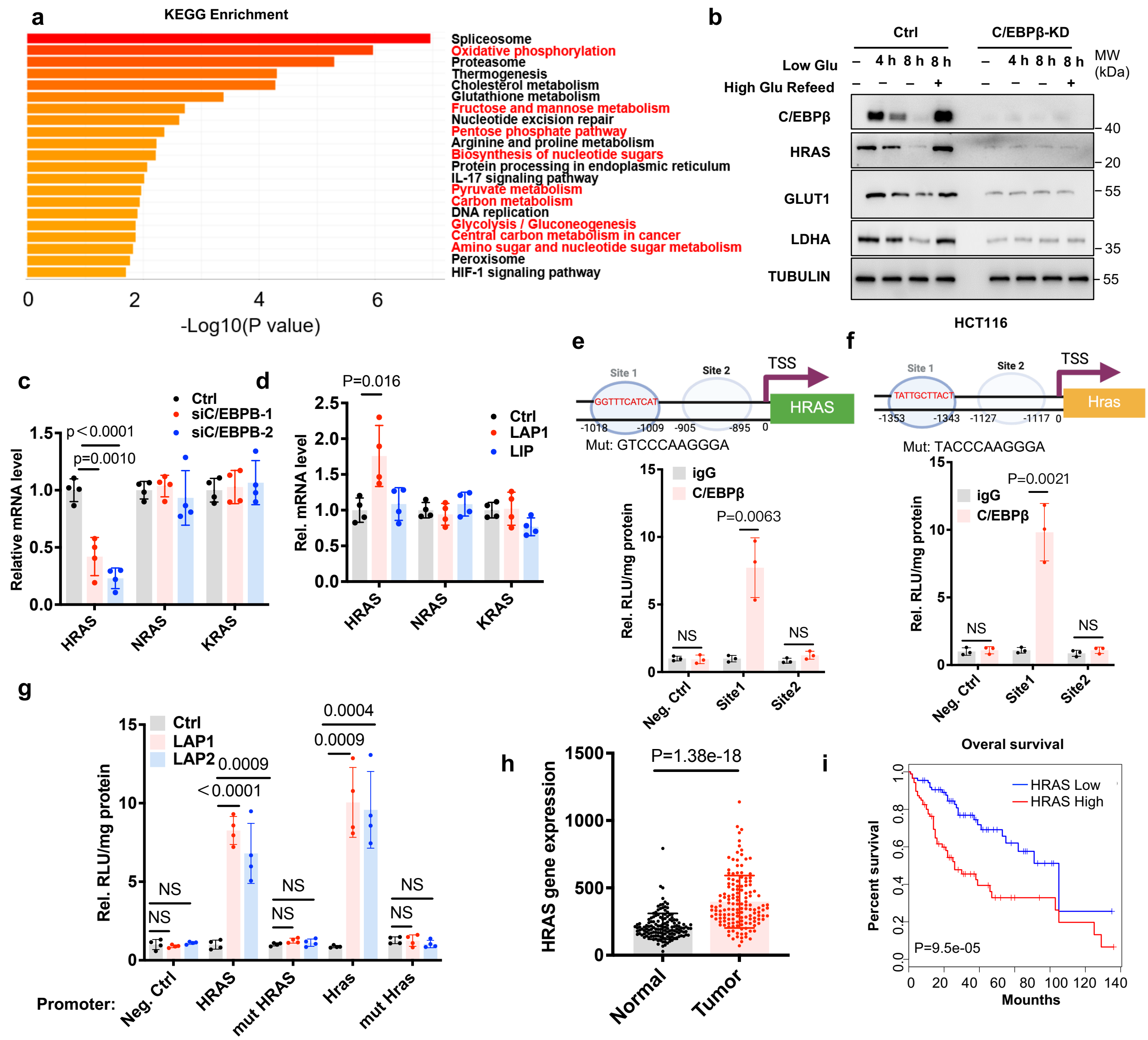

Supplementary Figure 7 | C/EBPβ-LAPs upregulate the expression of key glycolytic genes and HRAS in liver cancer cells.

a, KEGG enrichment of upregulated DEGs from HepG2 cells overexpressing LAP. Pathways related to glucose metabolism are highlighted in red.

b, Immunoblotting in Ctrl and C/EBPβ knocking down HCT116 cells starved by low glucose (2.8 mM) at indicated time, with/without glucose restoration (12.8 mM, 4 h) afterwards.

c, Relative mRNA levels of HRAS, NRAS and KRAS in siCtrl, siC/EBPβ-1 or siC/EBPβ-2 HepG2 cells. (N = 4 independent experiments)

d, Relative mRNA levels of HRAS, NRAS and KRAS in Ctrl, LAP1 or LIP-overexpressing HepG2 cells. (N = 4 independent experiments)

e, (top) JASPAR-predicted C/EBPβ binding sites on human HRAS promoter (region -2000 to +100 bp, related to TSS). (bottom) ChIP assay in HepG2 cells to detect the binding sites of C/EBPβ on the promoter (N = 3 independent experiments).

f, (top) JASPAR-predicted C/EBPβ binding sites on mouse Hras promoter (region -2000 to +100 bp, related to TSS). (bottom) ChIP assay in HepG2 cells to detect the binding sites of C/EBPβ on the promoter (N = 3 independent experiments).

g, Relative RLUs (Relative Light Units)/mg of protein of Ctrl, human HRAS, mutant human HRAS, mouse Hras and mutant mouse Hras promoters within luciferase reporter plasmids in HEK293 cells transfected with Ctrl, LAP1 or LAP2 (N = 4 independent experiments). The sequences of binding sites/mutant binding sites were illustrated in e, f.

h, HRAS gene expression in paired tumor and adjacent normal tissues of HCC patients from GEO, GTex, TCGA, and TARGET databases (N = 143 patients).

i, Kaplan-Meier curve showing overall survival percentages of high and low HRAS (cutoff: quartile) expression among HCC patients in the TCGA and the GTEx projects. The graph was created by GEPIA (N=159 patients).

Data are mean ± SD. P values were computed by two-sided unpaired *t* test (c-h) or two-sided log-rank test (i).

Schematics in **e** and **f** were created in BioRender. L, Y. (2026) https://BioRender.com/9egbl09. NS, non-significant.

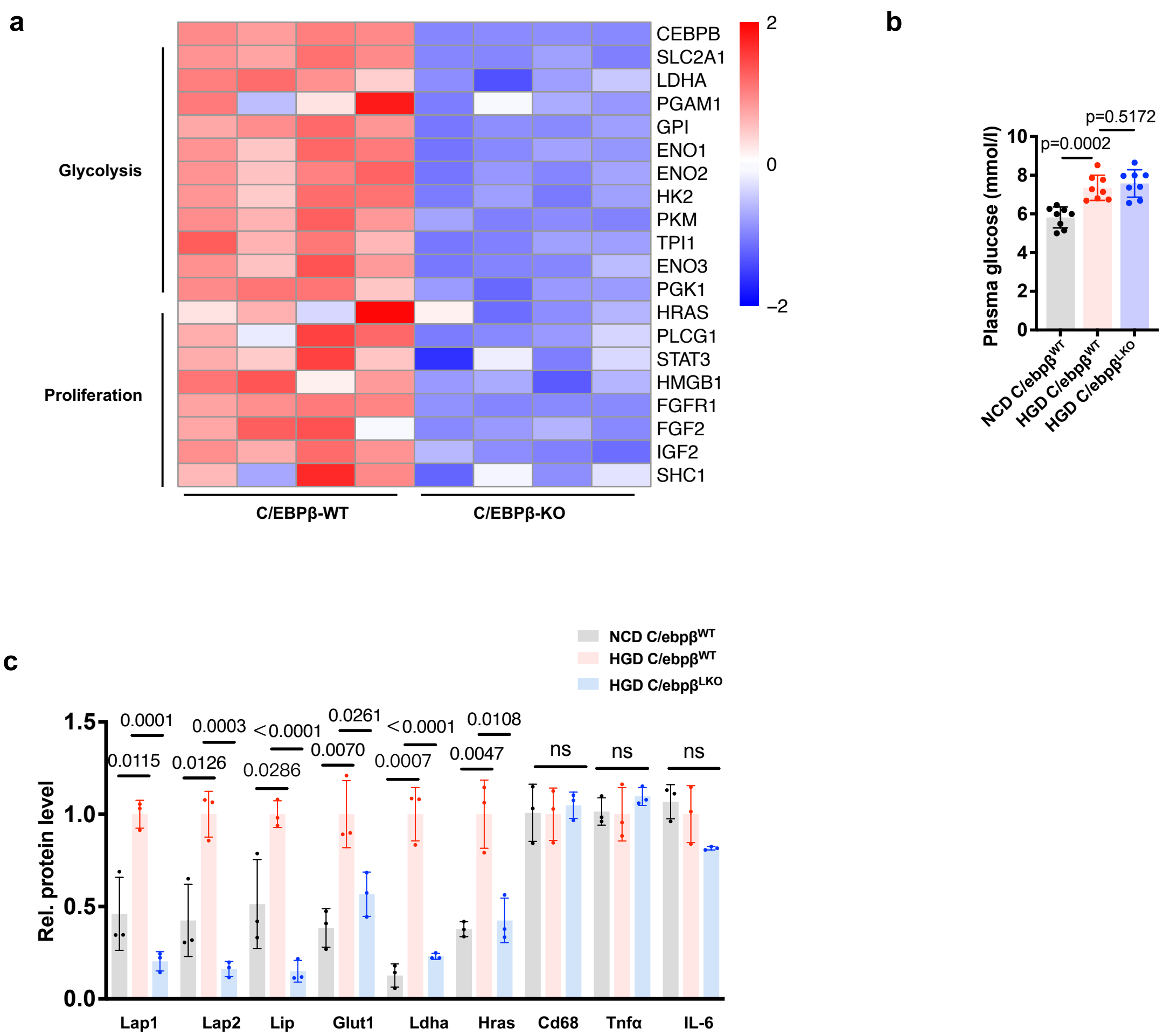

Supplementary Figure 8 | C/EBPβ is required for hyperglycemia-driven hepatocellular carcinoma development

a, Heatmap showing the normalized expression of the key glycolytic genes and proliferative genes in the RNA-seq results of WT or C/EBPβ-KO HepG2 cells.

b, Fasting plasma glucose levels in NCD C/ebpβ^{WT}, HGD C/ebpβ^{WT} and HGD C/ebpβ^{LKO} mice. Data are mean ± SD. N = 8 mice.

c, Quantification of immunoblotting in liver samples from normal chow diet (NCD) treated mice and high glucose diet (HGD) treated C/ebpβ^{WT} or C/ebpβ^{LKO} mice (related to Fig. 5h). N = 3 mice.

P values were computed by two-sided unpaired *t* test. NS, non-significant.

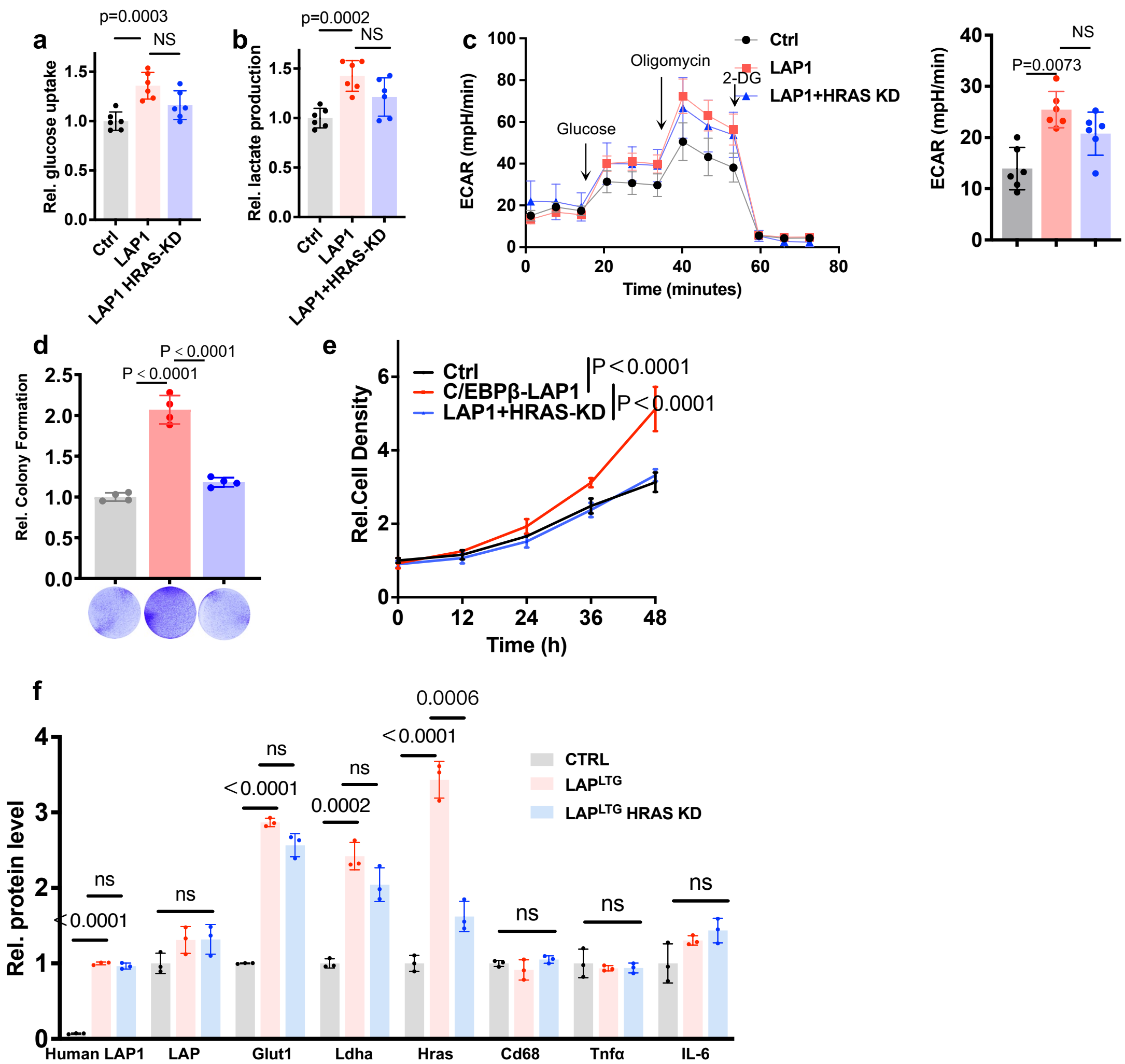

Supplementary Figure 9 | C/EBPβ-LAP1 upregulates glycolytic genes, Hras, and fuels HCC development.

a, b, Relative glucose uptake (a) and lactate production (b) of Ctrl, C/EBPβ-LAP1 and C/EBPβ-LAP1+HRAS-KD HepG2 cells. (N = 6 independent experiments)

c, (left) Extracellular acidification rates (ECARs) of Ctrl, C/EBPβ-LAP1 and C/EBPβ-LAP1+HRAS-KD HepG2 cells in response to glucose, oligomycin, and 2-deoxy-D-glucose (2-DG). (right) ECARs in response to glucose, which indicate glycolysis, of the HepG2 cells. (N = 6 independent experiments)

d-e, Relative colony formation (D) and growth curve (E) of Ctrl, C/EBPβ-LAP1 and C/EBPβ-LAP1+HRAS-KD HepG2 cells. (N = 4 independent experiments)

f, Quantification of immunoblotting in liver samples from Ctrl, $LAP1^{LTG}$ and $LAP1^{LTG}$ Hras-KD mice (related to Fig. 6c). N = 3 independent experiments

Data are mean ± SD. P values were computed by two-sided unpaired *t* test (a, b, c, d, f) or two-sided two-way ANOVA (e). NS, non-significant.

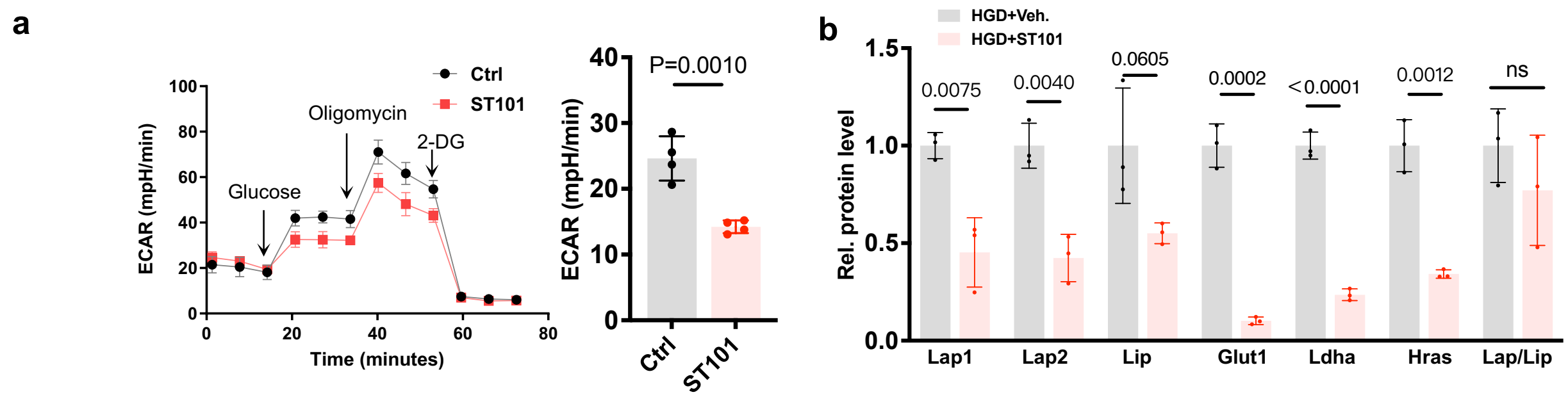

Supplementary Fig. 10 | C/EBPβ inhibition by ST101 attenuates glucose-driven HCC development.

a, (left) Extracellular acidification rates (ECARs) of HepG2 cells with/without ST101 (20 μM) treatment in response to glucose, oligomycin, and 2-deoxy-D-glucose (2-DG). (right) ECARs in response to glucose, which indicate glycolysis, of the HepG2 cells. Data are mean ± SD. P values were computed by unpaired *t* test. (N = 4 independent experiments)

b, Quantification of Immunoblotting in liver samples from high glucose diet (HGD) mice treated with/without ST101 (25 mg/kg administered three days per week by IV injection) (related to Fig. 7g). (N = 3 mice)

Data are mean ± SD. P values were computed by two-sided unpaired *t* test. NS, non-significant.

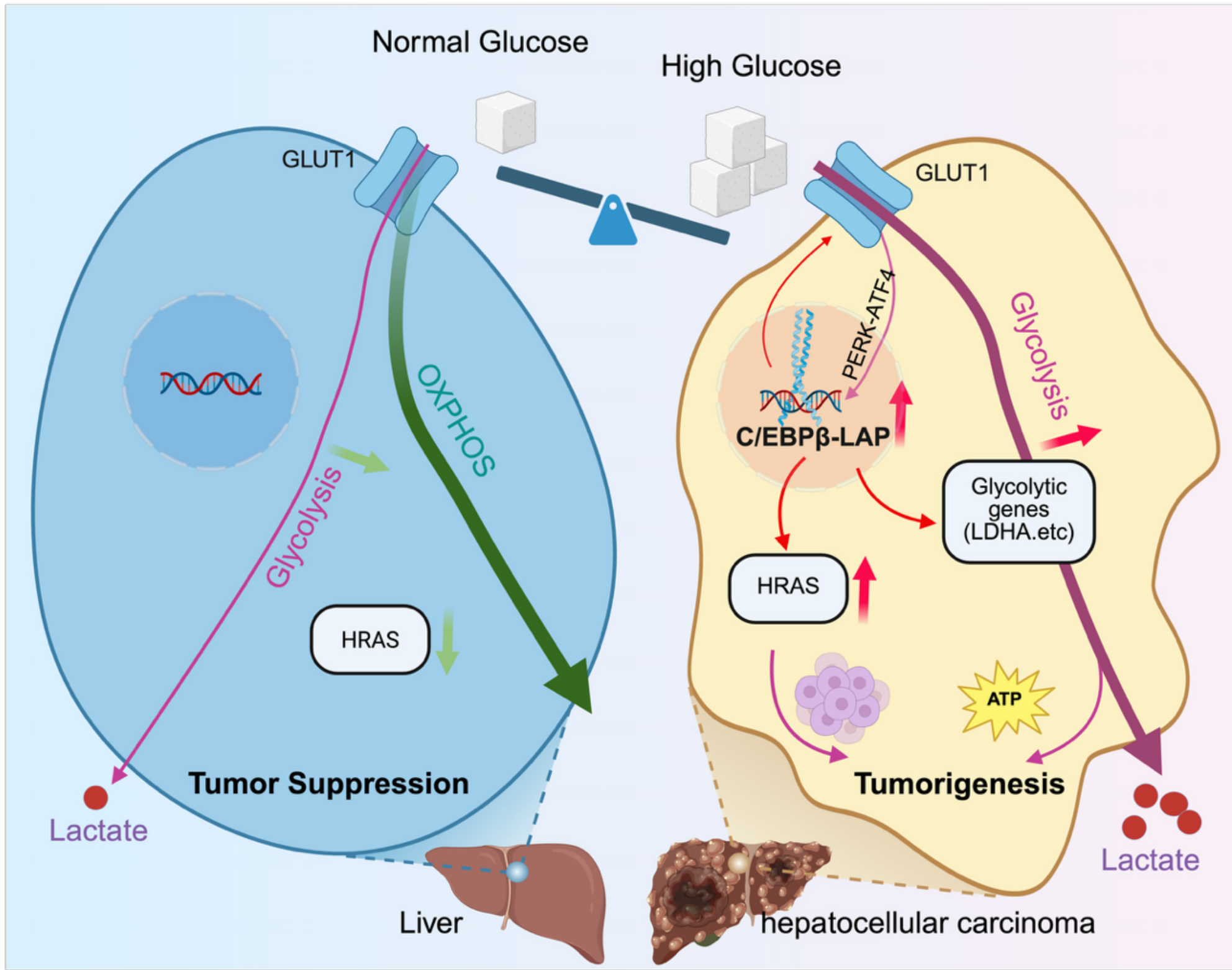

Supplementary Figure 11 | Scheme depicting the molecular mechanisms underlying hyperglycemia-elicited Tumor Progression. Schematic was created in BioRender. L, Y. (2026) https://BioRender.com/gyfa9av.

Supplementary Table 1.

Characteristics of LIHC patients, whose samples were collected for this study. Data are expressed as means ± SD. FPG, fasting plasma glucose. P values were computed by unpaired *t* test.

characteristic	Non-diabetic patients	Diabetic patients	P value
**Gender (Male/Female)**	15/2	15/2	n.a.
**Age (years)**	61.5±10.71	62.9±10.1	0.669
**FPG (μmol/l)**	5.82±0.93	7.42±2.80	0.021
**BMI (kg/m^2)**	22.3±2.7	23.7±2.6	0.127
**HCC stage (Phase 1/2/3/4)**	3/13/1/0	3/13/0/1	n.a.

Supplementary Table 2.

Non-diabetic patient sample information

Patient #	DM/non-DM	Gender	Age (years)	FPG (μmol/l)	BMI (kg/m^2)	HCC stage	Fatty liver disease
1	non-DM	Male	50	6.98	22.48	1	No
2	non-DM	Male	44	5.00	22.49	1	No
3	non-DM	Male	67	5.23	19.23	1	No
4	non-DM	Male	78	5.96	23.44	2	No
5	non-DM	Male	51	5.92	24.17	2	No
6	non-DM	Male	66	6.29	20.08	2	No
7	non-DM	Male	56	6.19	27.55	2	No
8	non-DM	Male	58	5.9	20.8	2	No
9	non-DM	Male	50	4.55	19.2	2	No
10	non-DM	Male	69	6.4	19.0	2	No
11	non-DM	Male	47	5.67	21.8	2	No
12	non-DM	Male	77	8.28	26.1	2	No
13	non-DM	Female	65	5.99	23.0	2	Fatty liver disease
14	non-DM	Male	59	4.27	19.1	3	No
15	non-DM	Male	76	5.36	26.4	2	No
16	non-DM	Male	68	5.91	23.1	2	No
17	non-DM	Female	65	5.11	20.9	2	No

Supplementary Table 3.

Diabetic patient sample information

Patient #	DM/non-DM	Gender	Age (years)	FPG (μmol/l)	BMI (kg/m²)	HCC stage	Fatty liver disease
1	Type 2 DM	Male	55	8.54	29.7	2	No
2	Type 2 DM	Male	71	14.65	27.1	2	No
3	Type 2 DM	Male	47	6.9	21.8	2	No
4	Type 2 DM	Female	86	8.33	21.4	2	No
5	Type 2 DM	Male	69	5.9	20.8	2	No
6	Type 2 DM	Male	67	4.9	27.7	2	No
7	Type 2 DM	Male	51	6.47	24.3	2	No
8	Type 2 DM	Male	61	5.49	25.1	2	No
9	Type 2 DM	Male	61	6.9	23.9	4	Fatty liver disease
10	Type 2 DM	Male	76	7.03	22.9	2	No
11	Type 2 DM	Female	68	8.87	27.11	1	Fatty liver disease
12	Type 2 DM	Male	69	14.57	22.96	2	No
13	Type 2 DM	Male	51	10.37	26.57	1	No
14	Type 2 DM	Male	62	10.17	21.08	1	No
15	Type 2 DM	Male	56	8.02	26.57	2	No
16	Type 2 DM	Male	60	9.15	25.30	2	No
17	Type 2 DM	Male	54	6.71	24.91	2	No

Supplementary Table 4.

PCR primers used in this study

Primer Name	Primer Sequence
hGLUT1-F	TGAGCATCGTGGCCATCTTT

hGLUT1-R	CCGGAAGCGATCTCATCGAA
hGPI-F	CTGGTAGACGGCAAGGATGTGA
hGPI-R	TCCGTGATGGTCTTGCCTGTGT
hPFKL-F	AAGAAGTAGGCTGGCACGACGT
hPFKL-R	GCGGATGTTCTCCACAATGGAC
hALDOA-F	GACACTCTACCAGAAGGCGGAT
hALDOA-R	GGTGGTAGTCTCGCCATTTGTC
hTPI1-R	CGAGCAGACAAAGGTCATCGCA
hTPI1-F	TCGGAGCTTCTCGTGTACTTCC
hGAPDH-F	GTCTCCTCTGACTTCAACAGCG
hGAPDH-R	ACCACCCTGTTGCTGTAGCCAA
hPGK1-F	CCGCTTTCATGTGGAGGAAGAAG
hPGK1-R	CTCTGTGAGCAGTGCCAAAAGC
hPGM1-F	TGATGGACGCGAGCAAACTGTC
hPGM1-R	ATGTCCTCCACACTCTGCTTGC
hENO1-F	AGTCAACCAGATTGGCTCCGTG
hENO1-R	CACAACCAGGTCAGCGATGAAG
hLDHA-F	GGATCTCCAACATGGCAGCCTT
hLDHA-R	AGACGGCTTTCTCCCTCTTGCT
hCEBPB-F	AGAAGACCGTGGACAAGCACAG
hCEBPB-R	CTCCAGGACCTTGTGCTGCGT
hIRE1a-F	CCGAACGTGATCCGCTACTTCT
hIRE1a-R	CGCAAAGTCCTTCTGCTCCACA
hATF6-F	CAGACAGTACCAACGCTTATGCC
hATF6-R	GCAGAACTCCAGGTGCTTGAAG
mCebpb-F	CAACCTGGAGACGCAGCACAAG
mCebpb-R	GCTTGAACAAGTTCCGCAGGGT
mGlut1-F	GCTTCTCCAACTGGACCTCAAAC
mGlut1-R	ACGAGGAGCACCGTGAAGATGA
mLDHA-F	ACGCAGACAAGGAGCAGTGGAA
mLDHA-R	ATGCTCTCAGCCAAGTCTGCCA
hHRAS-F	ACGCACTGTGGAATCTCGGCAG
hHRAS-R	TCACGCACCAACGTGTAGAAGG
hNRAS-F	GAAACCTCAGCCAAGACCAGAC
hNRAS-R	GGCAATCCCATACAACCCTGAG
hKRAS-F	CAGTAGACACAAAACAGGCTCAG
hKRAS-R	TGTCGGATCTCCCTCACCAATG
hNFKB1-F	GCAGCACTACTTCTTGACCACC
hNFKB1-R	TCTGCTCCTGAGCATTGACGTC
hATF4-F	TTCTCCAGCGACAAGGCTAAGG
hATF4-R	CTCCAACATCCAATCTGTCCCG
hACTIN-F	CACCATTGGCAATGAGCGGTTC
hACTIN-R	AGGTCTTTGCGGATGTCCACGT
mActin-F	CATTGCTGACAGGATGCAGAAGG
mActin-R	TGCTGGAAGGTGGACAGTGAGG
HRAS-R1-F	CACTGCAGCCGACTAAT
HRAS-R1-R	AGCGGGTGGATTACTTG
HRAS-R2-F	GTGAGCCACCGCGCC
HRAS-R2-R	CGCACATCCTAGCTCTTTCA
Hras-R1-F	TCCTGCCGTTCCCAGATA
Hras-R1-R	CGTCTGTGGGACAAGACAT
Hras-R2-F	GCCCCGCCCCCTAGC
Hras-R2-R	GTGCCTTCACTGCGTTCAG

Supplementary Table 5.

Antibodies and key reagents used in this study.

Antibodies & dilution	Source	Catalog number
C/EBP beta Antibody (H-7) (1 :200 for WB)	Santa Cruz	sc-7962
Purified anti-C/EBP β (3 isoforms) (1 :1000 for WB)	BioLegend	606202
Anti-GLUT1 antibody (1 :1000 for WB)	Abcam	ab115730
LDHA (C4B5) Rabbit mAb (1 :1000 for WB)	Cell Signaling	3582S
LDHA-Specific Polyclonal antibody (1 :1000 for WB)	Proteintech	19987-1-AP
Monoclonal Anti-Vinculin antibody (1 :5000 for WB)	MilliporeSigma	V9131
Alpha Tubulin Polyclonal antibody (1 :5000 for WB)	Proteintech	11224-1-AP
Anti-Ki67 antibody (1 :500 for IHC)	Abcam	ab15580
HRAS-Specific Polyclonal antibody (1 :1000 for WB)	Proteintech	18295-1-AP
NRAS Polyclonal antibody (1 :1000 for WB)	Proteintech	10724-1-AP
KRAS Polyclonal antibody (1 :1000 for WB)	Proteintech	12063-1-AP
p44/42 MAPK (Erk1/2) Antibody (1 :1000 for WB)	Cell Signaling	9102
Phospho-p44/42 MAPK (Erk1/2) (Thr202/Tyr204) Antibody (1 :1000 for WB)	Cell Signaling	9101
BiP (C50B12) Rabbit mAb (1 :1000 for WB)	Cell Signaling	3177
eIF2α Antibody (1 :1000 for WB)	Cell Signaling	9122
Phospho-eIF2α (Ser51) Antibody (1 :1000 for WB)	Cell Signaling	9121
ATF-4 (D4B8) Rabbit mAb (1 :1000 for WB)	Cell Signaling	11815S
C/EBPβ (LAP) Antibody (1 :1000 for WB)	Cell Signaling	3087S
Anti-CEBP Beta antibody [E299] (1 :1000 for IF)	Abcam	ab32358
IL-6 Polyclonal antibody (1 :1000 for WB)	Proteintech	21865-1-AP
Anti-CD3 epsilon antibody [SP7] (1 :1000 for WB)	Abcam	ab16669
Anti-TNF alpha antibody (1 :1000 for WB)	Abcam	ab6671
Anti-CD68 antibody (1 :1000 for WB)	Abcam	ab125212
Anti-c-Myc antibody [Y69] (1 :1000 for WB)	Abcam	ab32072
Cyclin D1 (92G2) Rabbit Monoclonal Antibody (1 :1000 for WB)	Cell Signaling	2978
**Reagents**	**Source**	**Catalog number**
The PROTEOSTAT Aggresome detection kit	Enzo Life	ENZ-51035-
Tamoxifen	Sigma-Aldrich	K100
Tauroursodeoxycholic Acid (TUDCA)	Selleck	T5648-1G
ISRIB	Selleck	N/A
Glucose Uptake-Glo Assay Kit	Progema	N/A
Lactate Colorimetric/Fluorometric Assay Kit	Biovision	J1341
BCA protein assay Kit	Beyotime	K607-100
ChIP Kit	Abcam	P0012
Mitoquinol	MedChemExpress	ab500
H2DCFDA (DCFH-DA)	MedChemExpress	HY-124410
Rapamycin	MedChemExpress	HY-D0940
ST101	QYAOBIO	HY-10219
High glucose diet	Topbiotech	N/A
STZ (Streptozotocin)	MedChemExpress	HY-13753
