# Supplementary material for: The transcription factor C/EBPβ promotes hyperglycemia-elicited glycolysis and liver cancer progression
Source: Nat Commun. 2026 Aug 1;17:9707. doi: 10.1038/s41467-026-76209-9 (PMC13562552; doi:10.1038/s41467-026-76209-9)
Supplement: Supplementary file 2 [file 41467_2026_76209_MOESM2_ESM.pdf]

nature portfolio

Corresponding author(s): Keqiang Ye

Last updated by author(s): Jun 16, 2026

Reporting Summary

Nature Portfolio wishes to improve the reproducibility of the work that we publish. This form provides structure for consistency and transparency in reporting. For further information on Nature Portfolio policies, see our Editorial Policies and the Editorial Policy Checklist.

Statistics

For all statistical analyses, confirm that the following items are present in the figure legend, table legend, main text, or Methods section.

n/a	Confirmed	
☐	☒	The exact sample size (*n*) for each experimental group/condition, given as a discrete number and unit of measurement
☐	☒	A statement on whether measurements were taken from distinct samples or whether the same sample was measured repeatedly
☐	☒	The statistical test(s) used AND whether they are one- or two-sided<br>*Only common tests should be described solely by name; describe more complex techniques in the Methods section.*
☒	☐	A description of all covariates tested
☐	☒	A description of any assumptions or corrections, such as tests of normality and adjustment for multiple comparisons
☐	☒	A full description of the statistical parameters including central tendency (e.g. means) or other basic estimates (e.g. regression coefficient) AND variation (e.g. standard deviation) or associated estimates of uncertainty (e.g. confidence intervals)
☐	☒	For null hypothesis testing, the test statistic (e.g. *F*, *t*, *r*) with confidence intervals, effect sizes, degrees of freedom and *P* value noted<br>*Give P values as exact values whenever suitable.*
☒	☐	For Bayesian analysis, information on the choice of priors and Markov chain Monte Carlo settings
☒	☐	For hierarchical and complex designs, identification of the appropriate level for tests and full reporting of outcomes
☒	☐	Estimates of effect sizes (e.g. Cohen's *d*, Pearson's *r*), indicating how they were calculated

Our web collection on statistics for biologists contains articles on many of the points above.

Software and code

Policy information about availability of computer code

Data collection	Images were acquired using a Zeiss LSM 980 confocal microscope.<br>RNA-seq samples were sequenced by lllumina NovaSeqTM X Plus.<br>^{18}F-FDG microPET scan in mice were performed by a high-resolution small animal PET scanning device (microPET)<br>RT-qPCR were performed by Applied Biosystems StepOnePlus TM Real-Time PCR instrument.<br>ECAR was measured using a Seahorse XF96 Analyzer (Seahorse Bioscience).<br>WB were were visualized by Bio-Rad ChemiDoc.
Data analysis	Immunofluorescence images were analyzed by ZEN 2.5 software.<br>WB images were analyzed by imageJ 1.54 software.<br>Graphpad Prism 10 was used for data analysis.<br>For RNA-seq data, gene expression (TPM) and differential analysis (DESeq2) were performed, with pathway enrichment (positive regulators of glycolysis) assessed by GSEA (clusterProfiler) and heatmaps created by pheatmap.<br>For FDG-PET analysis, the statistical results of standard uptake value (SUV) in the region of interesting (ROI) were calculated and analyzed by Amide 1.0.4-1 (San Diego, CA 92101)<br>ECAR data were analyzed using Seahorse WAVE.

For manuscripts utilizing custom algorithms or software that are central to the research but not yet described in published literature, software must be made available to editors and reviewers. We strongly encourage code deposition in a community repository (e.g. GitHub). See the Nature Portfolio guidelines for submitting code & software for further information.

Data

Policy information about availability of data

All manuscripts must include a data availability statement. This statement should provide the following information, where applicable:

- Accession codes, unique identifiers, or web links for publicly available datasets
- A description of any restrictions on data availability
- For clinical datasets or third party data, please ensure that the statement adheres to our policy

All data in this study are available within the paper. The Sequencing data (RNA-seq) generated in this study have been deposited in the ArrayExpress database under accession code E-MTAB-16207 (https://www.ebi.ac.uk/biostudies/arrayexpress/studies/E-MTAB-16207) and E-MTAB-16228 (https://www.ebi.ac.uk/biostudies/arrayexpress/studies/E-MTAB-16228). Source data are provided with this paper.

Research involving human participants, their data, or biological material

Policy information about studies with human participants or human data. See also policy information about sex, gender (identity/presentation), and sexual orientation and race, ethnicity and racism.

Reporting on sex and gender	Hepatocellular carcinoma tissues from 34 HCC patients (15 male and 2 female for each group) were used in this study.
Reporting on race, ethnicity, or other socially relevant groupings	These patients are Han Chinese Population, and there are NO variables on race, ethnicity or socially relevant groupings to report.
Population characteristics	Patients were stratified into two cohorts according to the presence or absence of pre-existing diabetes mellitus (DM). The two groups demonstrated comparable baseline characteristics with respect to age (61.5±10.71 years in non-diabetic patients vs. 62.9±10.1 years in diabetic patients), body mass index (22.3±2.7 vs. 23.7±2.6), and tumor stage distribution (comprising 3 phase I, 13 phase II, 1 phase III cases for non-DM group, 3 phase I, 13 phase II, 1 phase IV cases for DM group). However, as anticipated, fasting blood glucose levels were significantly elevated in the diabetic group compared to the non-diabetic group (7.42±2.80 mmol/L vs. 5.82±0.93 mmol/L).
Recruitment	hepatocellular carcinoma tissues and clinical data were obtained from Xinhua Hospital Affiliated to Shanghai Jiao Tong University and the First Affiliated Hospital of Guangxi Medical University.
Ethics oversight	Ethics approval was granted by the Institutional Review Board of Xinhua Hospital Affiliated to Shanghai Jiao Tong University (XHECC-2022-104-1) and the First Affiliated Hospital of Guangxi Medical University (2025-S0745). Patient information is listed in Supplementary Tables 1–3.

Note that full information on the approval of the study protocol must also be provided in the manuscript.

Field-specific reporting

Please select the one below that is the best fit for your research. If you are not sure, read the appropriate sections before making your selection.

- [x] Life sciences
- [ ] Behavioural & social sciences
- [ ] Ecological, evolutionary & environmental sciences

For a reference copy of the document with all sections, see nature.com/documents/nr-reporting-summary-flat.pdf

Life sciences study design

All studies must disclose on these points even when the disclosure is negative.

Sample size	Sample sizes for all experiments in this study were determined based on established standards in the field of biological and cancer research, as well as previous studies published in peer-reviewed journals. The aim was to ensure adequate statistical power to detect meaningful differences between treatment groups while also considering logistical and ethical constraints.<br>For the in vivo experiments focusing on tumor development in mice, A total of 8 mice per group were used. This sample size was selected based on guideline recommendations for experimental animal studies, which typically suggest a minimum of 5–10 animals per treatment group to achieve reliable data while minimizing variability.
Data exclusions	No data were excluded from the analysis.
Replication	All western blot data are representative of three or more experimental replicates. Other data (RT-qPCR, fluorescence staining) are also replicated with the number of samples shown in the plot as the number of individual datapoints.
Randomization	In genetic analysis, mice were grouped according to genetic identity. In other studies, mice were randomly grouped. Experimental conditions were clearly described in figure labels.
Blinding	Investigators were blinded to group allocation during data collection and/or analysis.

Reporting for specific materials, systems and methods

We require information from authors about some types of materials, experimental systems and methods used in many studies. Here, indicate whether each material, system or method listed is relevant to your study. If you are not sure if a list item applies to your research, read the appropriate section before selecting a response.

Materials & experimental systems

n/a	Involved in the study
☐	☒ Antibodies
☐	☒ Eukaryotic cell lines
☒	☐ Palaeontology and archaeology
☐	☒ Animals and other organisms
☒	☐ Clinical data
☒	☐ Dual use research of concern
☒	☐ Plants

Methods

n/a	Involved in the study
☒	☐ ChIP-seq
☒	☐ Flow cytometry
☒	☐ MRI-based neuroimaging

Antibodies

Antibodies used

C/EBP beta Antibody (H-7) Santa Cruz sc-7962 (1 :200 for WB)
Purified anti-C/EBP β (3 isoforms) BioLegend 606202 (1 :1000 for WB)
Anti-GLUT1 antibody Abcam ab115730 (1 :1000 for WB)
LDHA (C4B5) Rabbit mAb Cell Signaling 3582S (1 :1000 for WB)
LDHA-Specific Polyclonal antibody Proteintech 19987-1-AP (1 :1000 for WB)
Monoclonal Anti-Vinculin antibody MilliporeSigma V9131 (1 :5000 for WB)
Alpha Tubulin Polyclonal antibody Proteintech 11224-1-AP (1 :5000 for WB)
Anti-Ki67 antibody Abcam ab15580 (1 :500 for IHC)
HRAS-Specific Polyclonal antibody Proteintech 18295-1-AP (1 :1000 for WB)
NRAS Polyclonal antibody Proteintech 10724-1-AP (1 :1000 for WB)
KRAS Polyclonal antibody Proteintech 12063-1-AP (1 :1000 for WB)
p44/42 MAPK (Erk1/2) Antibody Cell Signaling 9102 (1 :1000 for WB)
Phospho-p44/42 MAPK (Erk1/2) (Thr202/Tyr204) Antibody Cell Signaling 9101 (1 :1000 for WB)
BiP (C50B12) Rabbit mAb Cell Signaling 3177 (1 :1000 for WB)
eIF2α Antibody Cell Signaling 9122 (1 :1000 for WB)
Phospho-eIF2α (Ser51) Antibody Cell Signaling 9121 (1 :1000 for WB)
ATF-4 (D4B8) Rabbit mAb Cell Signaling 11815S (1 :1000 for WB)
C/EBPβ (LAP) Antibody Cell Signaling 3087S (1 :1000 for WB)
Anti-CEBP Beta antibody [E299] Abcam ab32358 (1 :1000 for IF)
IL-6 Polyclonal antibody Proteintech 21865-1-AP (1 :1000 for WB)
Anti-CD3 epsilon antibody [SP7] Abcam ab16669 (1 :1000 for WB)
Anti-TNF alpha antibody Abcam ab6671 (1 :1000 for WB)
Anti-CD68 antibody Abcam ab125212 (1 :1000 for WB)
Anti-c-Myc antibody [Y69] Abcam ab32072 (1 :1000 for WB)
Cyclin D1 (92G2) Rabbit Monoclonal Antibody Cell Signaling 2978 (1 :1000 for WB)

Validation

All antibodies were validated by the manufacturer (typically cell type specific staining patterns) and are widely used in the field. C/EBPB antibody was validated using knockout and over expression experiments. HRAS-Specific Polyclonal antibody was validated using knockdown experiments. eIF2α Antibody and ATF-4 Antibody were validated using kover expression experiments.

Eukaryotic cell lines

Policy information about cell lines and Sex and Gender in Research

Cell line source(s): Human HepG2 (HB-8065), HCT116 (CCL-247EMT), and HEK293 (CRL-1573) cells were purchased from ATCC.

Authentication: None of the cells used were authenticated.

Mycoplasma contamination: All cell lines were tested negative for mycoplasma contamination

Commonly misidentified lines (See ICLAC register): No commonly misidentified lines were used in this study

Animals and other research organisms

Policy information about studies involving animals; ARRIVE guidelines recommended for reporting animal research, and Sex and Gender in Research

Laboratory animals	C57BL/6J mice were obtained from Jackson Laboratories. Albumin-CreERT2, C/ebpβ-flox, and Rosa26-LSL-LAP mice (C57BL/6J background) were purchansed from Cyagen. Only male mice were studied, reflecting hepatocellular carcinoma gender prevalence.Male mice received intraperitoneal N-nitrosodiethylamine (DEN; 1 mg/kg) at postnatal day 14, followed by twice-weekly carbon tetrachloride (CCl_4; 0.2 mL/kg i.p.). 100% of male mice developed hepatocellular carcinoma by 22 weeks.
Wild animals	No wild animals were used.
Reporting on sex	Only male mice were studied, reflecting hepatocellular carcinoma gender prevalence.
Field-collected samples	This study did not involve field-collected samples
Ethics oversight	Experiments were approved by the Shenzhen Institutes of Advanced Technology (SIAT-IACUC-240130-SLG-YKQ-A2465)

Note that full information on the approval of the study protocol must also be provided in the manuscript.

Plants

Seed stocks	*Report on the source of all seed stocks or other plant material used. If applicable, state the seed stock centre and catalogue number. If plant specimens were collected from the field, describe the collection location, date and sampling procedures.*
Novel plant genotypes	*Describe the methods by which all novel plant genotypes were produced. This includes those generated by transgenic approaches, gene editing, chemical/radiation-based mutagenesis and hybridization. For transgenic lines, describe the transformation method, the number of independent lines analyzed and the generation upon which experiments were performed. For gene-edited lines, describe the editor used, the endogenous sequence targeted for editing, the targeting guide RNA sequence (if applicable) and how the editor was applied.*
Authentication	*Describe any authentication procedures for each seed stock used or novel genotype generated. Describe any experiments used to assess the effect of a mutation and, where applicable, how potential secondary effects (e.g. second site T-DNA insertions, mosiacism, off-target gene editing) were examined.*
