# Supplementary material for: The transcription factor C/EBPβ promotes hyperglycemia-elicited glycolysis and liver cancer progression
Source: Nat Commun. 2026 Aug 1;17:9707. doi: 10.1038/s41467-026-76209-9 (PMC13562552; doi:10.1038/s41467-026-76209-9)
Supplement: Supplementary file 3 [file 41467_2026_76209_MOESM3_ESM.pdf]

nature portfolio

Peer Review File

The transcription factor C/EBPβ promotes hyperglycemia-elicited glycolysis and liver cancer progression

Corresponding Author: Professor Keqiang Ye

Parts of this Peer Review File have been redacted as indicated to remove third-party material.

This file contains all reviewer reports in order by version, followed by all author rebuttals in order by version.

Version 0:

Reviewer comments:

Reviewer #1

(Remarks to the Author)
The authors report that C/EBPβ-LAP1 and LAP2 are induced under hyperglycemic conditions. They employ LAP1 for further experiments, arguing that it substitutes for LAP1 and LAP2. They conclude that LAP1 promotes glycolysis and liver cancer progression through upregulating glycolytic effectors GLUT1, LDHA, and the oncoprotein HRAS. These findings are largely at odds with existing literature, describing oncogenic functions for LIP and tumor suppression functions for LAP2. My assessment is that the authors discovered functions of the LAP1 isoform that are specific for this protein but may not be translatable to functions of LAP2. If I am right, this may explain why the data are at odds with most of the published literature. The authors may disagree, however, in this case they should provide proof by adding a considerable number of experiments investigating the functions of LAP2. In addition, I find the data presented in Figure 1 not convincing; I will go into detail about it below.

Therefore, although the data are surprising and incomplete, they provide a potentially important extension of current knowledge about the function of LAP1.

Major concerns

Figure 1
A) The assertion that C/EBPβ expression in the DM group within the HCC-para samples cannot be made based on a single #10 showing upregulation of C/EBPβ. That C/EBPβ expression is barely detectable in most HCC-Para samples is also unexpected, given that the liver is a good tissue for examining C/EBPβ expression.

B) Comparing non-DM-T and DM-T gives a correlation due to the separated non-DM-T- and DM-T-specific grouping of C/EBPβ expression and glucose level correlations. However, the correlation between glucose levels and C/EBPβ expression levels should be assessed within the single groups. Within these separate groups there seems to be no correlation.

C) The same as under B; within a single group, there seems to be no correlation between LAP expression/function and GLUT1.
This is also obvious from the blots, shown in A. It is even worse; sample 14 of HCC-Tumor has high LIP levels and compared with sample 13 with low LIP levels the same levels of GLUT1, although LIP inhibits LAP function. Going through the LAP and GLUT1 signals one by one in the HDD-Tumor blot, there is no correlation between LAP and GLUT1 signal. Also, for the HCC-Pare group there is no correlation; GLUT1 expression is not higher in sample 10, as the only sample with high LAP expression, compared to samples 1, 4, 7, 9, 11, 12, 13 with low/no C/EBPβ expression.
In the same manner, there is also no correlation between LAP and LDHA expression.

Conceptually, it would have been better to compare LAP/LIP expression ratios with the other parameters because LAP/LIP determines the transcriptional function.

Based on the data presented in panels A-C it's difficult to conclude that in HCC or para-HCC patient samples there is a causative correlation between plasma glucose levels, C/EBPβ levels, and levels of GLUT1 or LDHA.

The number of samples is low, 7 per group. Possibly the data are compromised by differences in quality of the patient samples, or the data reflect that HCC is a highly heterogenous disease, with divers levels of infiltration of immune cells. In addition, as most genes ldha and glut1 are regulated through interactions between multiple transcription factors.

A possible solution could be provided by analyzing patient samples by immunostaining and pathological assessment of quality (e.g., immune cell infiltration, necrosis).

D) The expression of C/EBPβ is too low for proper assessment. In normal chow liver samples C/EBPβ is easily detectable. LAP1, LAP2, LIP, GLUT1 and LDHA expression levels should be quantified and normalized against the loading control vinculin, and this signal should be of lower exposure (all signals within linear range).

E) Also, here quantification of LAP1, LAP2, LIP, GLUT1 and LDHA expression is important for proper interpretation of the data.

For the blots comparing the expression it should be described whether the same blot was used or different blots were used.

Figure 2
What puzzles me most is that there are several studies showing that low glucose is correlated with ISR and eIF2α-phosphorylation in cell culture models, and high glucose is associated with ISR absence.

However, in diabetic models, high glucose was shown to induce ISR through secondary ER stress. Has been shown by the David Ron lab (doi: 10.1016/j.cmet.2008.04.011).

In all experiments displayed only the LAPs are shown. LIP should be shown as well. This is an easy fix since the signal must be on the same blots. In the context of PERK-eIF2α regulation it is particular interesting to see what happens with LIP expression. Previous studies by others have shown that high PKR (another eIF2α-kinase)-eIF2α-phosphorylated results in low LIP levels, while unphosphorylated eIF2α facilitate LIP expression (DOI: 10.1101/gad.14.15.1920; DOI: 10.1038/srep42603).

G) this experiment offers an excellent setting for proving the involvement of C/EBPβ in GLUT1/LDHA regulation. C/EBPβ KD should prevent GLUT1 and LDHA upregulation in response to high glucose (in addition to Figure 4F).

Page 11, line 199
It is surprising and problematic that LAP1 was chosen for further experiments, because it has extra functions that have been described in the literature. LAP1 functionally differs from LAP2 in recruiting the SWI/SNF chromatin remodeling complex, making genomic sites accessible for further regulation (DOI: 10.1016/s1097-2765(00)80384-6). The recruitment of the SWI/SNF complex is important for LAP1 interaction with MYB in hematopoietic cells (DOI: 10.1016/s1097-2765(00)80384-6). MYB transcription factors are major oncogenes in HCC and they have been shown to induce glycolysis (Warburg effect), See DOI: 10.31487/j.BMC.2025.01.09; doi.org/10.3389/fonc.2021.592045; doi: 10.1080/21655979.2021.2017630, and other papers.
Therefore, it is a valid hypothesize that LAP1 facilitates the oncogenic function of MYB, which explains the phenotypes observed in this study.

To safely conclude that LAP1 substitutes for the function of both LAPs in enhancing glucose uptake in HepG2 cells and in displaying the pro-oncogenic activities, all critical experiments should be repeated with LAP2.

It is also important to note that LIP overexpression in several other cell systems, including a mouse hepatoma cell line, shows the opposite; that LIP induces a glycolytic flux and increases cell proliferation and cell survival. DOI: 10.1038/s42003-019-0461-z; DOI: 10.1016/j.molmet.2023.101726

Moreover, genetic depletion of LIP in mice results in a shift towards whole-body enhanced fat metabolism and reduced carbohydrate metabolism (DOI: 10.15252/embr.201439837; DOI: 10.3389/fragi.2021.738512).
Another study has shown that LIP is required to overcome repression of E2F targets by long C/EBPβ (LAP2) and C/EBPα (p42) proteins, and that LIP deficiency delays the entry into the cell cycle of regenerating hepatocytes (DOI: 10.1101/gad.557910).
Furthermore, LIP deficient mice display a reduced and delayed cancer incidence, and LIP overexpressing mice have an increased cancer incidence (including liver) (DOI: 10.7554/eLife.34985; DOI 10.1007/s00109-014-1215-5).

Altogether the conclusion from most literature is that LIP is the oncogene and LAP2 has tumor suppression functions.

Page 16, Extended Data Figure 4H-K
The authors rush over the regulation of the HRAS promoter by C/EBPβ-LAP1.
Concerning the reporter assays they do not provide information about the part of the promoters used. This should be included. In addition, since they show the regulation, it must be possible to identify the positions of the binding sites using the consensus binding sequence (A/G T T G C G C/T A A A/G). By mutation of those binding sites the authors can provide

proof that the regulation is through direct binding of LAP1.
The MotifMap (https://motifmap.ics.uci.edu) does not list C/EBP binding sites for HRAS

Are there data available in ChIP-Seq databases that support the observation that HRAS contains C/EBPβ binding sites? I did not find any.

Page 16, Figure 5
For panel H, in addition to LAP1/2 LIP should be shown.
Also here is the question whether LAP2 would give the same results. Without the experiments with LAP2 the data are difficult to interpret.

Page 19, Figure 6
If the used Rosa26-LSL-LAP allele expresses LAP1(?), the experiments show that LAP1 stimulates HCC in vivo. But again, what about LAP2?

In addition, ideally one would also like to know the effect of LIP expression, since LIP overexpression in another mouse model increases cancer incidence and blunted LIP expression in another mouse model reduces cancer incidence.

Page 22, Figure 7
Please also show LIP in blots of panels B and G, and the exposure should of the C/EBPβ blot B could be stronger.
The authors mention the mouse model is hyperglycemia-induced HCC progression. However, how was HCC induced? Are these LAP1-LTP mice? HCC induced with DEN/CC14?

Methods
Animals: What is exactly in the Rosa26-LSL-LAP allele? LAP1 or LAP2 sequences?

Reviewer #2

(Remarks to the Author)
This study identifies the transcription factor C/EBPB as a missing mechanistic link between hyperglycemia and accelerated hepatocellular carcinoma (HCC) progression. By delineating the ROS-pERK-eIF2alpha-ATF4 signaling axis as the primary driver of C/EBPB upregulation, the authors provide a molecular explanation for the poor prognosis observed in diabetic cancer patients. They have a good amount of mouse data and patient data to help drive the importance of studying this pathway. Furthermore, the successful suppression of tumor growth using the clinical-phase peptide ST101 validates it as a high-value therapeutic target, offering a precision medicine approach to treat glucose-fueled malignancies.

• The in vitro data indicates an increase in Glut1, but the patient data in figure 1a indicates no difference in diabetic vs non. How do the authors reconcile these results?
• In many of their western blots, the authors switched between vinculin and tubulin as their housekeeping proteins, but it is not clear why.
• In Figure 1a, while I see their trend in the HCC tumor, in the HCC paracancerous tissue, it seems like it is just one patient that is driving the increase in C/EBPbeta expression more than in the tumor samples. As such, those blots do not appear to validate their claim that C/EBPbeta LAP increases in HCC paracancerous tissue. Also, it is not clear why they included the paracancerous tissue.
• The authors only use the high-glucose diet mouse model for their model of hyperglycemia (normally regarded as a type 2 diabetes model). In their intro they indicate cancer mortality is not affected by the type of diabetes. Can they validate their results in a type 1 model to make sure that difference is still there between C/EBPbeta protein expression, the glycolytic enzyme expression, and those downstream ROS-PERK-eIF2α-ATF4 axis proteins they investigate with their high glucose diet model.
• The authors only use the patient data to investigate the increased expression of C/EBPbeta LAP and the glycolytic-linked proteins, but they should also test the patient data to determine if the downstream proteins they measure in Figure 2 are also elevated in the patient samples to strengthen their in vitro/in vivo comparison.
• Convention issue: the authors use n instead of N when they are referring to biological replicates
• It is not a metastasis paper (as it mostly focuses on the primary tumor), but if the authors are seeing a difference in metastasis between their HGD and NCD conditions, or at least differences in invasion potential or migratory phenotype instead of just growth and proliferation, and the opposite trend when targeting their protein of interest, would also make their story more compelling.

Reviewer #3

(Remarks to the Author)
This manuscript describes the mechanism by which hyperglycemia promotes the development of hepatocellular carcinoma (HCC). The authors present evidence linking hyperglycemia to the activation of the transcription factor C/EBPβ. They demonstrate that increased glucose levels lead to ROS-dependent activation of the PERK–eIF2α–ATF4 signaling pathway, resulting in enhanced transcription of C/EBPβ. Elevated levels of C/EBPβ subsequently promote glycolysis and cell proliferation in liver cancer through the upregulation of key glycolytic effectors and the oncoprotein HRAS. Furthermore, the authors illustrate that C/EBPβ overexpression drives HCC development, while genetic deletion or pharmacological

inhibition of C/EBPβ reduces hyperglycemia-driven HCC progression.

Overall, the manuscript is well-structured and thoughtfully designed, with a clear and thorough experimental approach. The conclusions are strong for regulation, however the data do NOT support mediation. This needs to be corrected throughout the manuscript text, figure legends, the title, and the discussion section.

Major Concerns

1. The phrasing "by driving glycolysis and proliferation" implies a mechanistic causal pathway. The authors can only use this construction if the data directly demonstrate that glycolysis and proliferation mediate the phenotype downstream of C/EBPβ in a causality reversal experiment. The following statements need to be edited, or experiments need to be performed to prove causality. A few examples are noted below, but the authors are required to edit throughout.

Title: C/EBPβ Modulates Hyperglycemia-elicited Tumor Progression by Promoting Glycolysis
3 and Cell Proliferation - replace BY with AND

Extended Data Fig 6. C/EBPb-LAP fuels HCC development AND (not by) upregulating glycolytic genes and RAS.

2. C/EBPβ is known to regulate pro-inflammatory molecules and influence immune responses, which are relevant in oncogenesis. It would be informative to evaluate downstream inflammatory effects, such as cytokine expression (IL-6, TNFα) or markers of immune cell infiltration (e.g., F4/80 or CD68 in mice, CD3/CD8 in human samples). This would clarify whether hyperglycemia-driven C/EBPβ activity promotes an inflammatory microenvironment that supports HCC development.

3. The authors convincingly demonstrate that C/EBPβ upregulates HRAS across multiple models and that its knockdown attenuates HCC. It would strengthen the manuscript to examine whether other oncogenes, such as c-MYC or CCND1, are also regulated by C/EBPβ. Since p53 is known to be inhibited by C/EBPβ, it would be interesting to look at p53 levels in the patient samples.

4. For the patient cohort in extended figure 1, are the cancer stages for the patients known? Does the "phase 1/phase 2" designation address this? If so, please clarify. Furthermore, is there information on whether the patients have diabetes alone or if they fall within the spectrum of non-alcoholic fatty liver disease (NAFLD)? This information could be particularly valuable given the interplay between metabolism, inflammation, and tumor development.

5. In the TCGA and GTEx cohorts analyzed in extended data 2, it would be helpful to report whether C/EBPβ mRNA differs between tumor and non-tumor samples or across stages. Stratification by diabetes status (if possible) could further clarify the relevance of hyperglycemia. If the mRNA expression data do not closely correlate with the protein levels (like fig 1a and extended fig 1c), the potential for regulation at the post-translational level should be discussed.

Minor Concerns

6. Some references require verification. For instance, the citations related to elevated fasting glucose and liver cancer risk (line 59) and the strong epidemiological link between HCC, colon adenocarcinoma, and hyperglycemia or diabetes mellitus (line 114) need to be clarified.

7. The introduction/discussion section would benefit from a clearer overview of the current understanding of the roles of C/EBPβ and its isoforms, including LIP and the LAPs, in hepatocellular carcinoma. At present, it is difficult to place the findings in the context of existing literature. If the role of C/EBPβ in HCC remain poorly characterized, explicitly stating this would help emphasize the novelty and significance of the authors' observations.

8. In addition, it would be valuable to include a discussion of how ST101 is thought to act on C/EBPβ isoforms, particularly LIP and LAP1/2, and how this mechanism may be relevant in the context of hyperglycemia-driven HCC. A brief paragraph outlining the known or proposed effects of ST101 on C/EBPβ isoform balance, and how this could intersect with metabolic stress or hyperglycemia-induced signaling pathways, would strengthen the mechanistic interpretation of the therapeutic data presented in this study.

9. Edit to avoid overstatement of the results. "Thus, C/EBPβ is a critical regulator and therapeutic target for glucose-fueled cancer progression by driving glycolysis and proliferation". Please change to potential therapeutic target.

Reviewer #4

(Remarks to the Author)

I co-reviewed this manuscript with one of the reviewers who provided the listed reports. This is part of the Nature Communications initiative to facilitate training in peer review and to provide appropriate recognition for Early Career Researchers who co-review manuscripts.

Version 1:

Reviewer comments:

Reviewer #1

(Remarks to the Author)
The authors have adequately addressed all of my concerns and suggestions. In particular, the increased sample size has substantially strengthened the statistical power of the analyses, and the statistical evaluation has been appropriately updated. Although C/EBPβ detection in non DM para samples remains relatively low, the increase in signal in DM para samples is now clearly discernible (as shown in referee Fig. 1).
With respect to the distinct functions of LAP1 versus LAP2, the authors provide sufficient evidence to support the conclusion that, in this experimental context, LAP1 can functionally substitute for both isoforms. I recognize that generating additional mouse models to further dissect isoform specific roles would go beyond a reasonable effort for the scope of this study.
Overall, this study, together with previous work, supports the view that the physiological and pathological functions of C/EBPβ isoforms are highly context dependent. While the mechanistic basis for this context specificity remains incompletely understood, it provides a strong rationale and motivation for further investigation.

Reviewer #2

(Remarks to the Author)
Overall the authors have done a good job in responding to reviews and increasing the rigor of their results.

Reviewer #3

(Remarks to the Author)
The authors have performed additional experiments and clarified/edited where requested.

Open Access This Peer Review File is licensed under a Creative Commons Attribution 4.0 International License, which permits use, sharing, adaptation, distribution and reproduction in any medium or format, as long as you give appropriate credit to the original author(s) and the source, provide a link to the Creative Commons license, and indicate if changes were made.
In cases where reviewers are anonymous, credit should be given to 'Anonymous Referee' and the source.
The images or other third party material in this Peer Review File are included in the article's Creative Commons license, unless indicated otherwise in a credit line to the material. If material is not included in the article's Creative Commons license and your intended use is not permitted by statutory regulation or exceeds the permitted use, you will need to obtain permission directly from the copyright holder.
To view a copy of this license, visit https://creativecommons.org/licenses/by/4.0/

Point-to-point response to reviewers' comments

First, we would like to express our gratitude to the expert and detail-oriented reviewers, whose comments have significantly contributed to strengthening our manuscript. We believe that the revisions we have made will adequately address their comments and suggestions, substantially enhancing the quality of our work.

Responses to Reviewer #1 can be found on **Page 1**, responses to Reviewer #2 are on **Page 12**, and responses to Reviewers #3 are on **Page 15**. Our answers are in **blue** font.

Reviewer #1 (Remarks to the Author):

The authors report that C/EBPβ-LAP1 and LAP2 are induced under hyperglycemic conditions. They employ LAP1 for further experiments, arguing that it substitutes for LAP1 and LAP2. They conclude that LAP1 promotes glycolysis and liver cancer progression through upregulating glycolytic effectors GLUT1, LDHA, and the oncoprotein HRAS. These findings are largely at odds with existing literature, describing oncogenic functions for LIP and tumor suppression functions for LAP2. My assessment is that the authors discovered functions of the LAP1 isoform that are specific for this protein but may not be translatable to functions of LAP2. If I am right, this may explain why the data are at odds with most of the published literature. The authors may disagree, however, in this case they should provide proof by adding a considerable number of experiments investigating the functions of LAP2. In addition, I find the data presented in Figure 1 not convincing; I will go into detail about it below.

Therefore, although the data are surprising and incomplete, they provide a potentially important extension of current knowledge about the function of LAP1.

Thank you for your positive remarks regarding our findings! In response to your concerns, we have conducted comparative studies on the functions of LAP1, LAP2, and LIP. These studies clarify the isoform specificity and roles of these proteins in our research, addressing the potential discrepancies with existing literature. In Figure 1, We have substantially increased the number of samples to improve the statistical power of our analyses. We believe that these revisions will adequately address the raised concerns and significantly enhance the quality of our work. Thank you again for the valuable input, which has significantly improved our manuscript.

Please see the details below.

Major concerns

Figure 1

A) The assertion that C/EBPβ expression in the DM group within the HCC-para samples cannot be made based on a single #10 showing upregulation of C/EBPβ. That C/EBPβ expression is barely detectable in most HCC-Para samples is also unexpected, given that the liver is a good tissue for examining C/EBPβ expression.

In the previous version of the manuscript, we analyzed paracancerous and tumor tissues together. Because C/EBPβ expression was relatively enriched in the tumor group, its signal in the paracancerous group was generally weak under the same exposure conditions. The original purpose of including paracancerous tissue was to investigate whether C/EBPβ is also upregulated in non-tumor tissue. Therefore, indeed, direct comparisons with tumor tissue were not necessary.

Therefore, we reanalyzed the paracancerous tissues separately using a longer exposure time. In the revised manuscript, these results show that C/EBPβ expression is increased in the DM-paracancerous group compared with the non-DM paracancerous control group (**Referee Fig 1** or Extended Data Fig 2). We have revised the relevant text and figure presentation accordingly.

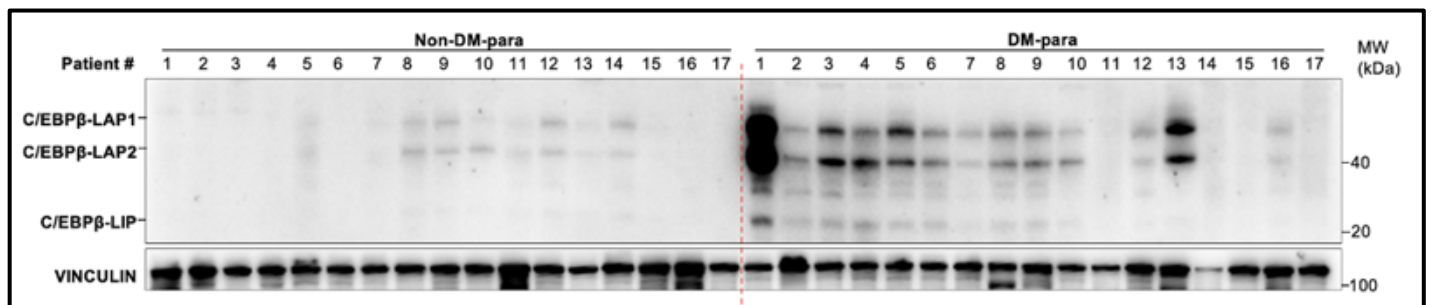

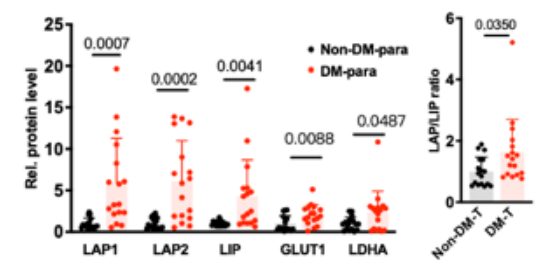

Referee Fig. 1 (Extended Data Fig. 2). **Increased hepatic C/EBPβ expression in HCC patients with DM. a, b,** Immunoblotting (a) and Quantification (b) in paracancerous tissue (Para) from Hepatocellular Carcinoma (HCC) patients with or without Diabetes mellitus (DM).
c, The LAP/LIP ratio in paracancerous tissue from HCC patients with or without DM. LAP/LIP ratio was calculated by adding LAP1 and LAP2 and dividing this value by LIP.

B) Comparing non-DM-T and DM-T gives a correlation due to the separated non-DM-T- and DM-T-specific grouping of C/EBPβ expression and glucose level correlations. However, the correlation between glucose levels and C/EBPβ expression levels should be assessed within the single groups. Within these separate groups there seems to be no correlation.

We agree that assessing the correlation between glucose levels and C/EBPβ expression within each individual group is more appropriate. In the previous version of the manuscript, the correlation between glucose levels and C/EBPβ expression within each single group was not statistically significant, likely because of the limited sample size, although a similar trend was observed.

To address this concern, we expanded our sample size through collaboration with Xinhua Hospital Affiliated to Shanghai Jiao Tong University and The First Affiliated Hospital of Guangxi Medical University. As a result, the sample size in each group increased from 7 to 17 patients (patient information is provided in the Supplementary Table 1-3, or find in the end of this response letter).

By analyzing these new and old samples together (**Referee Fig 2 a-c,** or Fig. 1 in manuscript), we performed correlation analyses within each group separately. The revised results show that fasting plasma glucose (FPG) levels are significantly correlated with C/EBPβ-LAP1/2 expression in both non-DM and DM groups **(Referee Fig 2 d,e,g,h or** Fig. 1&S3 in manuscript**).** In contrast, C/EBPβ-LIP showed a positive correlation with FPG levels only in the non-DM group, but not in the DM group **(Referee Fig 2 f,i,** or Extended Data Fig 3**).** We have revised the relevant text and figure presentation accordingly (Page 6, line 117, in red).

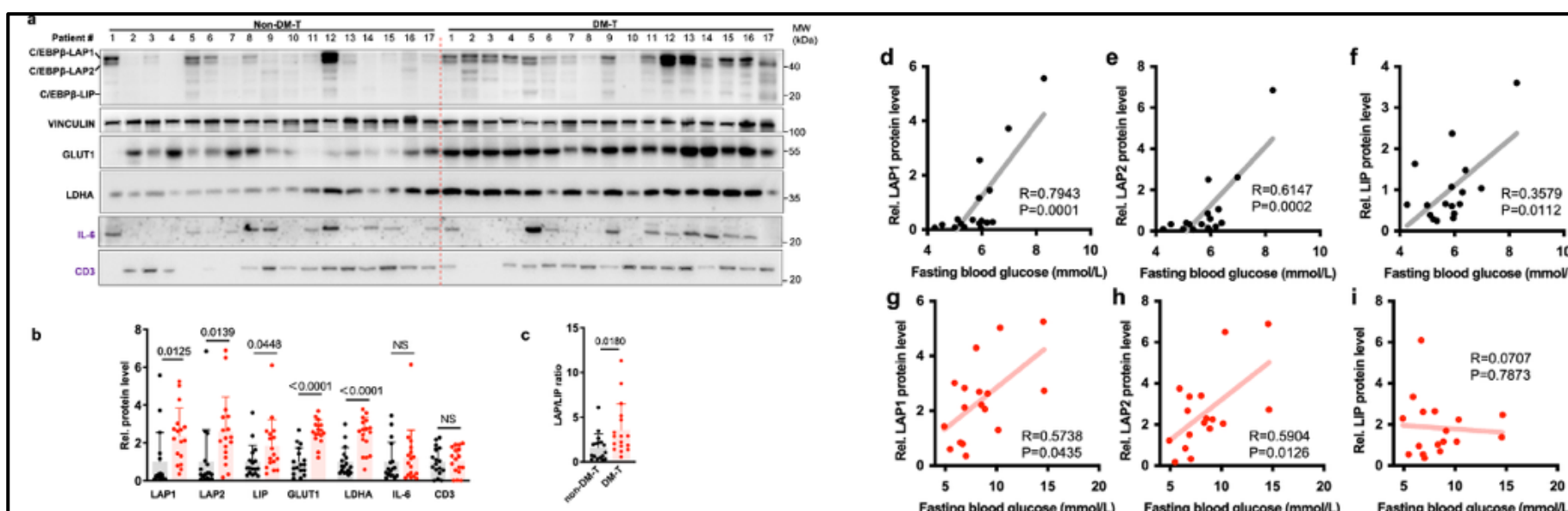

Referee Fig. 2 (Fig 1 & Extended Data Fig. 3). **Increased hepatic C/EBPβ expression in HCC patients with DM.**
a, **b,** Immunoblotting **(a)** and Quantification **(b)** in tumor (T) from Hepatocellular Carcinoma (HCC) patients with or without Diabetes mellitus (DM). **c,** The LAP/LIP ratio in tumor from HCC patients with or without DM. LAP/LIP ratio was calculated by adding LAP1 and LAP2 and dividing this value by LIP. **d-i,** Correlation analysis of FPG levels and C/EBPβ-LAP1 **(d)**, LAP2 **(e)**, and LIP **(i)** levels of **paracancerous** tissue in HCC patients. **g-i,** Correlation analysis of FPG (fasting plasma glucose) levels and C/EBPβ-LAP1 **(g)** /LAP2 **(h)/ LIP (i)**levels of **tumor** tissue in HCC patients.

C) The same as under B; within a single group, there seems to be no correlation between LAP expression/function and GLUT1.
This is also obvious from the blots, shown in A. It is even worse; sample 14 of HCC-Tumor has high LIP levels and compared with sample 13 with low LIP levels the same levels of GLUT1, although LIP inhibits LAP function. Going through the LAP and GLUT1 signals one by one in the HDD-Tumor blot, there is no correlation between LAP and GLUT1 signal.
Also, for the HCC-Pare group there is no correlation; GLUT1 expression is not higher in sample 10, as the only sample with high LAP expression, compared to samples 1, 4, 7, 9, 11, 12, 13 with low/no C/EBPβ expression.

In the same manner, there is also no correlation between LAP and LDHA expression.

We agree that conclusions regarding the relationship between C/EBPβ isoforms and glycolytic regulators should be based on correlation analyses performed within each individual group.

Similarly, To address this concern, we expanded the sample size and repeated the correlation analyses within the single DM-T group. In the revised manuscript, we found that C/EBPβ-LAP1 expression was significantly correlated with both GLUT1 and LDHA expression in DM-T samples (**Referee Fig 3 e,h**, Extended Data Fig. 3). Although LAP2 showed a tendency toward correlation with GLUT1 and LDHA, these associations did not reach statistical significance (**Referee Fig 3 f,i**). By contrast, LIP did not show a significant correlation with GLUT1 or LDHA (**Referee Fig 3 g,j**).

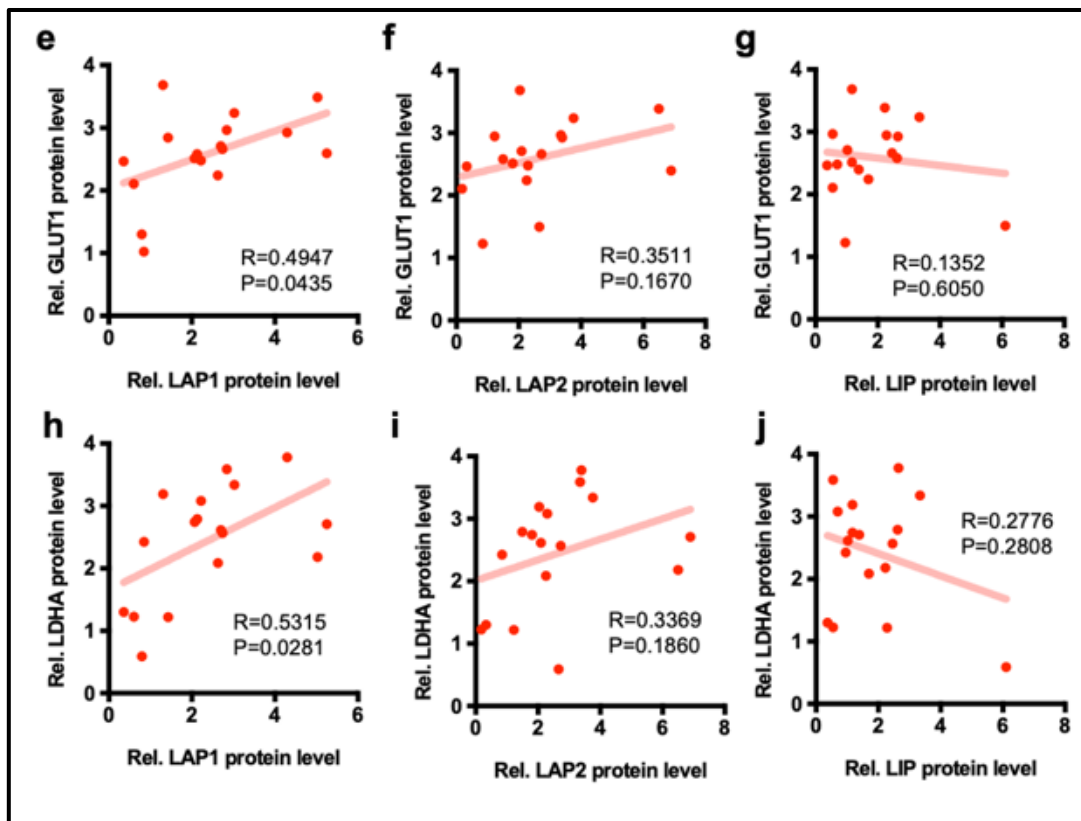

Referee Fig. 3 (Extended Data Fig. 3). Correlation analysis of C/EBPβ-isoforms levels and GLUT1 levels **(e-g)**/ LDHA levels **(h-j)** of tumor tissues in HCC patients.

Correlation analysis alone provides insights, but the regulatory relationship is established through experimental evidence. Therefore, we have rearranged the manuscript to mention this correlation (**Page 10, Line 207**) only after presenting the direct evidence of C/EBPβ driving the expression of GLUT1 and LDHA. This evidence is derived from our functional experiments, including RNA-seq, RT-qPCR, Western blotting, and luciferase assays, as presented in Fig. 3 & 4.

Conceptually, it would have been better to compare LAP/LIP expression ratios with the other parameters because LAP/LIP determines the transcriptional function.

Thank you for this constructive suggestion that LAP/LIP ratio is a more informative parameter, as it better reflects the overall transcriptional activity of C/EBPβ. Accordingly, in the revised manuscript, we have included the LAP/LIP ratio analysis for all relevant blots in Fig. 1 and revised the corresponding text. Based on this analysis, we found that the LAP/LIP ratio is significantly increased in the DM-T group compared with the DM control group (also see in referee Fig. 2c). This result is consistent with our overall findings that LAP isoforms, rather than LIP, are the major drivers of glycolysis in the diabetic condition (Fig. 3 and 4 in manuscript). We have revised the relevant text and figure presentation accordingly (Page 6, line 111, in red).

Based on the data presented in panels A-C it's difficult to conclude that in HCC or para-HCC patient samples there is a causative correlation between plasma glucose levels, C/EBPβ levels, and levels of GLUT1 or LDHA.

We agree that the data presented in panels A–C in the previous manuscript do not establish a causal relationship between plasma glucose levels, C/EBPβ expression, and GLUT1 or LDHA expression. In the revised manuscript, we have therefore strengthened the analysis by expanding the sample size and performing correlation analyses only within individual groups, which support the association between glucose levels, C/EBPβ, and glycolytic markers (Referee Fig 2-3). Importantly, a causal regulatory role of C/EBPβ does not rely on the correlation analyses. Rather, it is supported by our mechanistic studies, including siRNA knocking down, RNA-seq, RT-qPCR, Western blotting, and luciferase assays, which show that high glucose directly regulates C/EBPβ expresstion, and LAP1 overexpression directly increases GLUT1 and LDHA expression. We have revised the manuscript thoroughly to make this distinction clearer.

The number of samples is low, 7 per group. Possibly the data are compromised by differences in quality of the patient samples, or the data reflect that HCC is a highly heterogenous disease, with divers levels of infiltration of immune cells. In addition, as most genes ldha and glut1 are regulated through interactions between multiple transcription factors.

A possible solution could be provided by analyzing patient samples by immunostaining and pathological assessment of quality (e.g., immune cell infiltration, necrosis).

Thank you for this very thoughtful comment! We totally agree that the initial sample size was limited and that HCC is a highly heterogeneous disease, with potential variability arising from sample quality, immune cell infiltration, and other microenvironmental factors. We also agree that GLUT1 and LDHA are regulated by multiple transcription factors, which makes interpretation of clinical samples more complex.

To address this concern, we expanded sample size to strengthen the robustness of our conclusions. In addition, following the suggestions from you and the other reviewers, we further evaluated the immune-related heterogeneity of the HCC samples by analyzing markers such as IL6, TNFα and CD3 (**Referee Figure 4**, or Extended Data Fig 1). These results confirm the heterogeneous nature of HCC samples; however, T-cell infiltration (CD3) and inflammatory effects (IL-6 and TNFα) were comparable between the DM and non-DM groups, suggesting that these increases were not caused by differences in the inflammatory microenvironment. Therefore, although inter-sample heterogeneity is indeed present, our data suggest that the observed increases in C/EBPβ, GLUT1, LDHA, and the LAP/LIP ratio, as well as their correlations, are unlikely to be explained simply by differences in sample quality or immune infiltration between groups. We have clarified this point in the revised manuscript (Page 6, Line 115, in red).

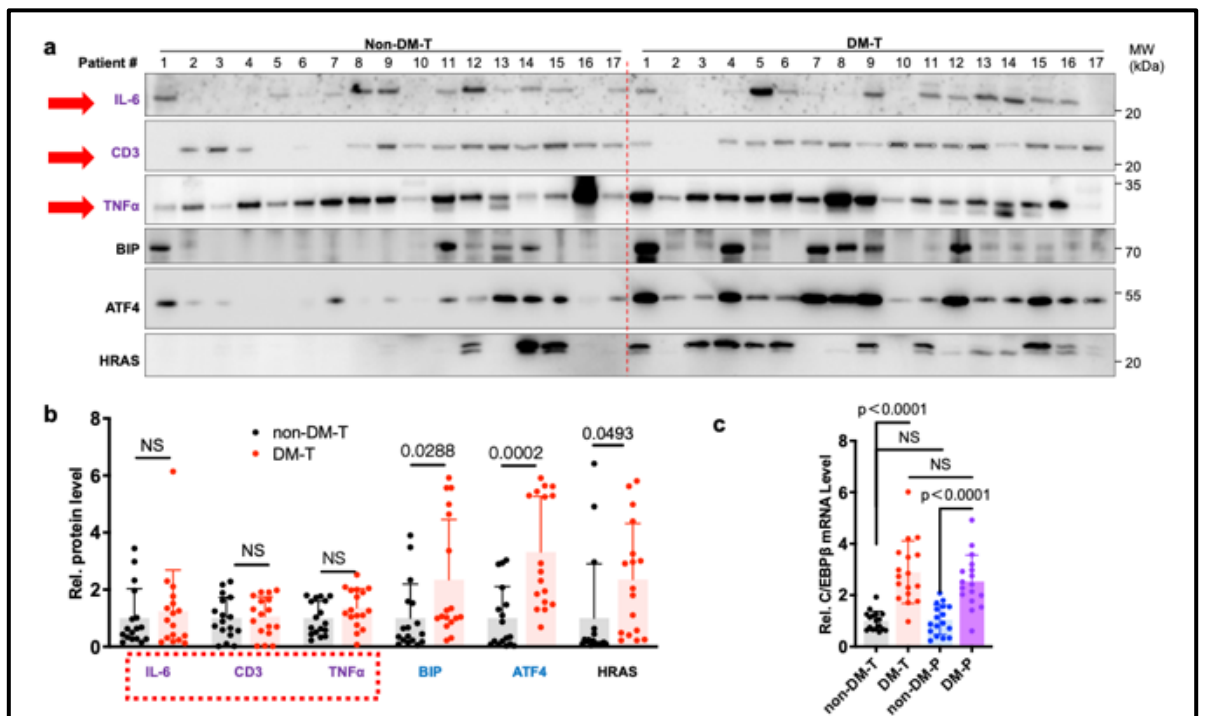

Referee Fig. 4 (Extended Data Fig 1) **a, b,** (related to Fig. 1a) Immunoblotting **(a)** and Quantification **(b)** in tumor (T) from Hepatocellular Carcinoma (HCC) patients with or without Diabetes mellitus (DM).

D) The expression of C/EBPβ is too low for proper assessment. In normal chow liver samples C/EBPβ is easily detectable. LAP1, LAP2, LIP, GLUT1 and LDHA expression levels should be quantified and normalized against the loading control vinculin, and this signal should be of lower exposure (all signals within linear range).

We agree that the original blot exposure was not optimal for proper assessment of C/EBPβ expression. In the revised manuscript, we have replaced the images with blots obtained at more appropriate exposure times to ensure that the signals are within the linear range and therfore more suitable for interpretation (**Referee Fig 5**, or Fig 1f in manuscript).

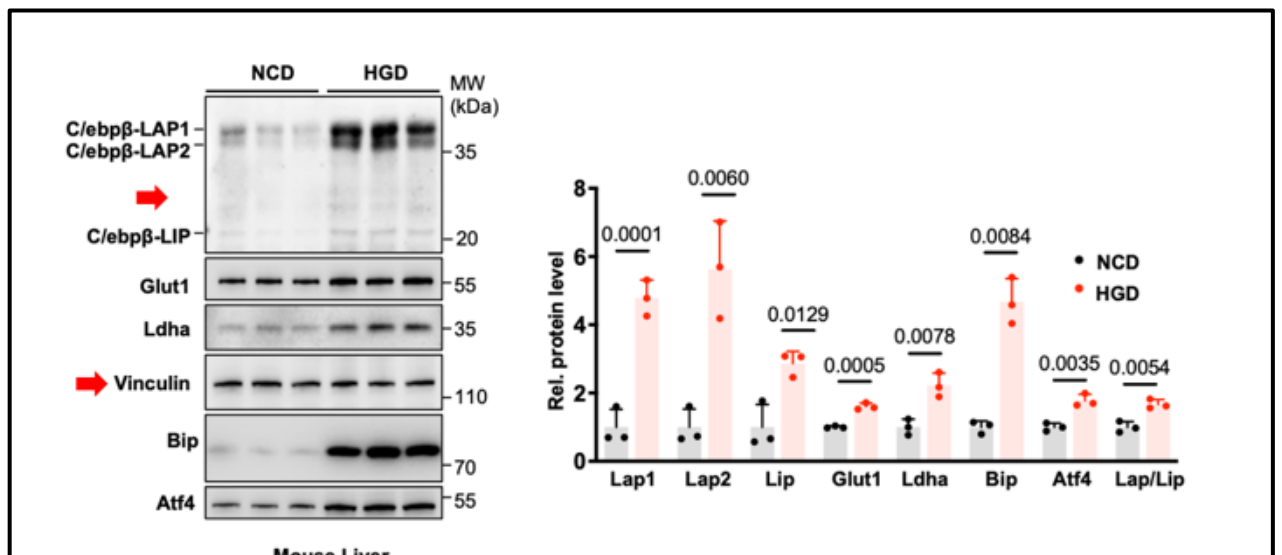

Referee Fig. 5 (Fig. 1f) Immunoblotting (left) and Quantification (right) in liver samples from normal chow diet (NCD) treated mice and high glucose diet treated mice (HGD).

In addition, we quantified LAP1, LAP2, LIP, GLUT1, and LDHA expression levels and normalized them to the loading control vinculin. The updated results show that LAP1, LAP2, LIP, GLUT1, and LDHA are upregulated in the mouse liver under the high-glucose diet condition.

E) Also, here quantification of LAP1, LAP2, LIP, GLUT1 and LDHA expression is important for proper interpretation of the data.

Thank you for this helpful comment! Indeed, quantitative analysis of LAP1, LAP2, LIP, GLUT1, and LDHA expression is important for proper interpretation of the Western blot data.

Accordingly, in the revised manuscript, we have quantified these Western blot results to improve data interpretation (**Referee Fig. 6** or Extended Data Fig 5). The analysis shows that LAP, GLUT1, and LDHA expression levels increase following high-glucose treatment in HepG2 cells. Interestingly, LAP and LIP expression peaks at 8 hours of treatment and does not increase further in the subsequent 16 hours; however, LIP expression decreases rapidly after 8 hours of treatment.

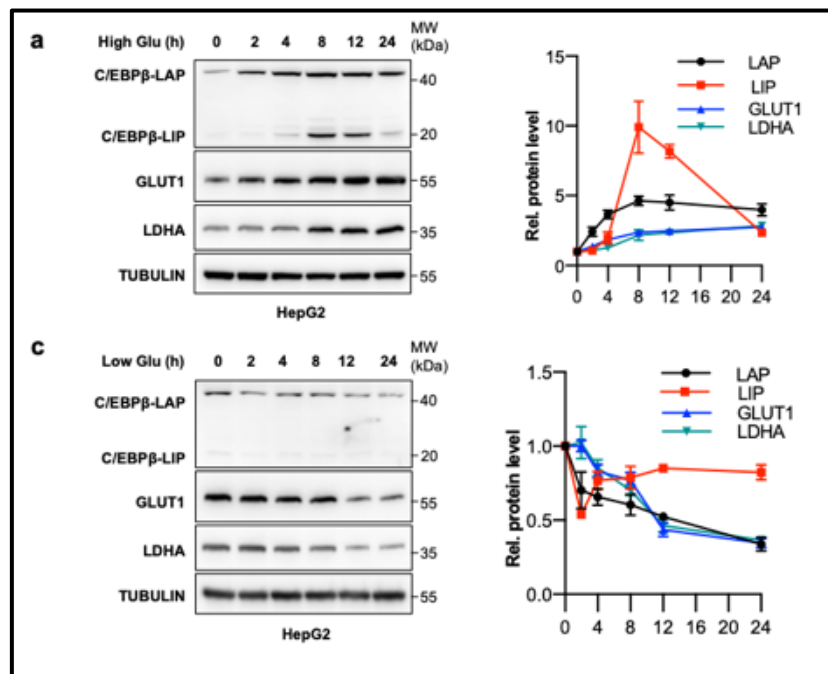

Referee Fig. 6 (Extended Data Fig 5) **High glucose activates the expression of C/EBPβ LAPs in cancer cell lines. a,** Immunoblotting in HepG2 cells treated with high glucose (12.8 mM) at the indicated time. **c,** Immunoblotting **(c)** and mRNA expression **(d)** in HepG2 cells starved with low glucose (2.8 mM) at the indicated time.

For the blots comparing the expression it should be described whether the same blot was used or different blots were used.

We have added a description in the Methods section under "Immunoblotting" (**Page 27, Line 596**) to clarify whether the same blot was used or if different blots were utilized for the expression comparisons.

Figure 2
What puzzles me most is that there are several studies showing that low glucose is correlated with ISR and eIF2α-phosphorylation in cell culture models, and high glucose is associated with ISR absence.

However, in diabetic models, high glucose was shown to induce ISR through secondary ER stress. Has been shown by the David Ron lab (doi: 10.1016/j.cmet.2008.04.011).
In all experiments displayed only the LAPs are shown. LIP should be shown as well. This is an easy fix since the signal must be on the same blots. In the context of PERK-eIF2α regulation it is particular interesting to see what happens with LIP expression. Previous studies by others have shown that high PKR (another eIF2α-kinase)-eIF2α-phosphorylated results in low LIP levels, while unphosphorylated eIF2α facilitate LIP expression (DOI: 10.1101/gad.14.15.1920; DOI: 10.1038/srep42603).

We appreciate the reviewer's insightful comments regarding the interplay between glucose levels, Integrated Stress Response (ISR)/eIF2α phosphorylation, and C/EBPβ regulation. We also noticed that glucose restriction can promote ISR and eIF2α phosphorylation through mechanisms such as N-linked glycosylation inhibition in neuronal cells or MEF[1,2]. We thank the reviewer for highlighting that diabetes or hyperglycemia can also induce eIF2α phosphorylation by eliciting ER stress[3] (as illustrated in Referee Fig. 7a). Moreover, several studies, together with this study, have demonstrated that high glucose levels are correlated with UPR (unfolded protein response) activation and eIF2α phosphorylation. Increased phosphorylation of eIF2α is often associated with cellular stress, including ER stress, which can be triggered by high glucose levels and contribute to the development of ISR[4,5]. Thus, it appears that both high glucose and glucose deprivation can potentially promote ISR and eIF2α phosphorylation, albeit through different mechanisms. Interestingly, this possibly partially explain how the progression of hepatocellular carcinoma (HCC) can be promoted by either hyperglycemia or hypoglycemia[6] (as noted in Referee Fig. 5b), while normal blood glucose levels are associated with the lowest incidence of cancer.

REDACTED

Phosphorylation of eIF2α decreases the translation of LIP, shifting the balance toward the longer LAP1 and LAP2 isoforms, while unphosphorylated eIF2α facilitates LIP expression[7,8]. Additionally, calorie restriction (CR) has been shown to decrease LIP expression via mTOR inhibition[9]. Furthermore, the work by David

Ron's lab[3] also demonstrated that ISR enhances the translation of C/EBPβ-LAP isoforms. In our study on hyperglycemia-induced HCC progression, we observed that high glucose indeed elicits ER stress and upregulates C/EBPβ at both protein and mRNA levels. By knocking down various key UPR-responsive elements, we determined that the PERK-eIF2α-ATF4 axis is required for glucose-induced upregulation of C/EBPβ mRNA, resulting in increased levels of all three isoforms (as shown in Referee Fig. 7c, or Fig. 2 of the manuscript). **We have now included data for all LAPs and LIPs in Figures 1, 2, 5, and 7.**

Therefore, we have expanded our discussion on the relationship between glucose, ISR/eIF2α and how C/EBPβ isoforms respond to ISR/eIF2α signaling (**Page18 Line402**, in red). We believe that these modifications will strengthen our manuscript by providing a clearer depiction of the complex relationships between glucose levels, ISR, and C/EBPβ isoform regulation, ultimately enhancing the understanding of their roles in cancer progression. We sincerely thank the reviewer for identifying this weakness in the manuscript and for providing key literature references.

G) this experiment offers an excellent setting for proving the involvement of C/EBPβ in GLUT1/LDHA regulation. C/EBPβ KD should prevent GLUT1 and LDHA upregulation in response to high glucose (in addition to Figure 4F).

We completely agree that this experiment is crucial for establishing the role of C/EBPβ in mediating GLUT1/LDHA responses to high glucose. In our previous manuscript (Fig. 4H and extended Fig. 4B in the previous manuscipt), we have aready conducted these experiments. Specifically, C/EBPβ knockout (KO) or knockdown (KD) effectively prevented the changes in GLUT1 and LDHA expression in response to glucose starvation and high glucose treatment in both HepG2 and HCT116 cells(as Shown in **Referee Fig. 8** or Fig 4h and Extended Data Fig. 7b). We also show here that the changes in C/EBPβ are NOT caused by loading differences, as Blotting VINCULIN and C/EBPβ together shows no difference in loading (**Referee Fig. 8b**).

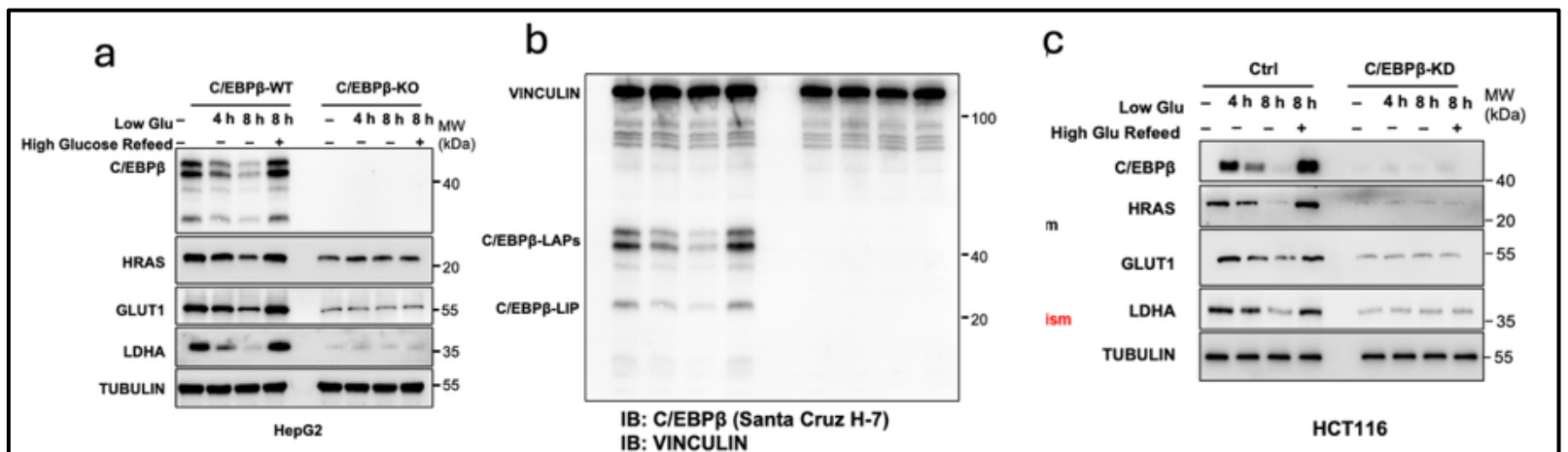

Referee Fig. 8 (Fig 4h, and Extended Fig 7b) **a,** Immunoblotting in C/EBPβ-WT and C/EBPβ-KO HepG2 cells starved by low glucose (2.8 mM) at the indicated time, with/without glucose restoration (12.8 mM, 4 h) afterwards. **b,** Blotting VINCULIN and C/EBPβ together for the blot in **a**. **c,** Immunoblotting in C/EBPβ-WT and C/EBPβ-KD HCT116 cells starved by low glucose (2.8 mM) at the indicated time, with/without glucose restoration (12.8 mM, 4 h) afterwards.

To enhance the clarity of our findings, we have now rearranged these figures to better highlight these key results. Thank you for emphasizing the significance of these experiments!

Page 11, line 199

It is surprising and problematic that LAP1 was chosen for further experiments, because it has extra functions that have been described in the literature. LAP1 functionally differs from LAP2 in recruiting the SWI/SNF chromatin remodeling complex, making genomic sites accessible for further regulation (DOI: 10.1016/s1097-2765(00)80384-6). The recruitment of the SWI/SNF complex is important for LAP1 interaction with MYB in hematopoietic cells (DOI: 10.1016/s1097-2765(00)80384-6). MYB transcription factors are major oncogenes in HCC and they have been shown to induce glycolysis (Warburg effect), See DOI: 10.31487/j.BMC.2025.01.09; doi.org/10.3389/fonc.2021.592045; doi: 10.1080/21655979.2021.2017630, and other papers.

Therefore, it is a valid hypothesize that LAP1 facilitates the oncogenic function of MYB, which explains the phenotypes observed in this study.

To safely conclude that LAP1 substitutes for the function of both LAPs in enhancing glucose uptake in HepG2 cells and in displaying the pro-oncogenic activities, all critical experiments should be repeated with LAP2.

We sincerely thank the reviewer for highlighting the important differences between LAP1 and LAP2, particularly regarding LAP1's ability to recruit the SWI/SNF chromatin remodeling complex[10] and the potential involvement of LAP1-MYB signaling in HCC progression[10-12].

In response to this concern, we conducted a comprehensive analysis comparing the functions of LAP1, LAP2, and LIP in terms of glycolysis, proliferation, and gene regulation in HepG2 cells (**Referee Fig. 9**, or Fig 3, Fig 4 and Extended Fig 7 in manuscript). The results clearly demonstrate that both LAP1 and LAP2, but not LIP, enhance glycolytic flux, promote cell proliferation, and increase the expression of relevant genes, such as GLUT1 and LDHA. These findings suggest that both LAP isoforms, independent of the SWI/SNF chromatin remodeling complex, contribute to HCC progression by upregulating glycolysis and supporting cellular proliferation.

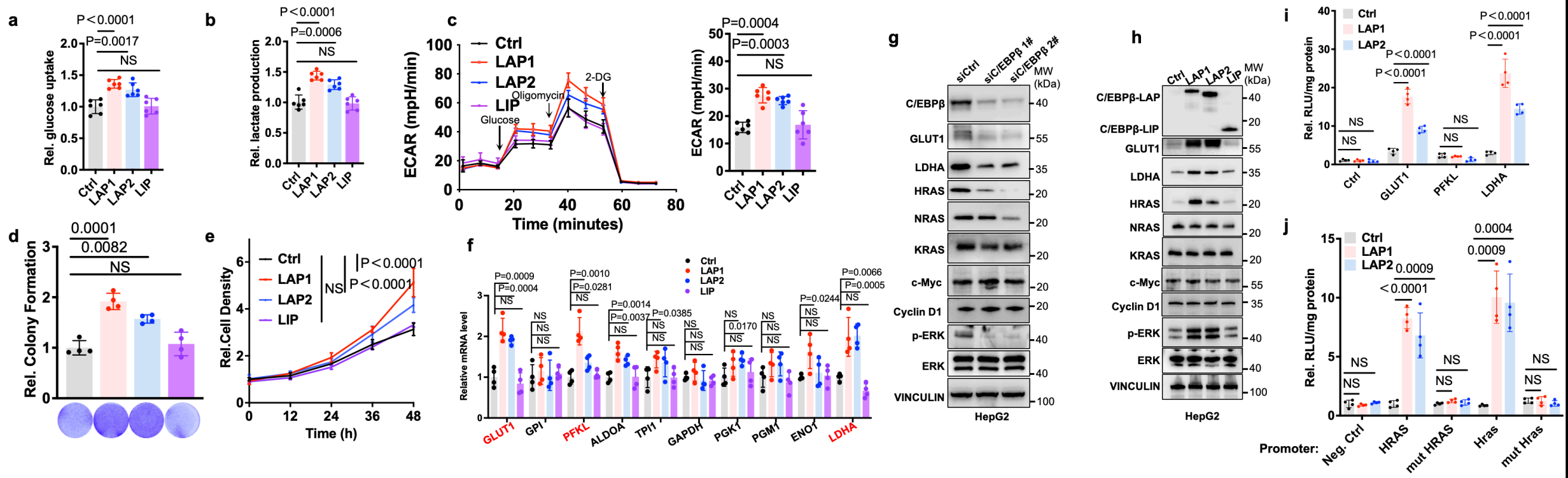

Referee Fig. 9 C/EBPβ-LAP1 and LAP2 upregulate the expression of key glycolytic genes and HRAS in liver cancer cells. **a-e,** related to Fig. 3 a-e; **f**, related to Fig. 4d; **g,h**, related to Fig 4, e-f; **i,** related to Fig. 4g; **j,** related to Extended Fig. 7 g.

For the *in vivo* studies, we utilized Rosa26-LSL-LAP1 mice to conditionally express LAP1 in hepatocytes. Establishing another mouse model for LAP2 would require an additional 1-2 years, which was not feasible within the scope of this project. Therefore, we plan to establish mouse models for conditional expression of LAP2 and LIP in future studies to comprehensively compare all three isoforms of C/EBPβ. We have to safely conclude that the C/EBPβ isoform LAP1 is a critical regulator and a potential therapeutic target for glucose-fueled cancer progression through driving glycolysis and cell proliferation.

We appreciate the reviewer's tremendous help about this and believe that our expanded studies provide robust evidence for our conclusions. We have now replaced the data, modified the text accordingly **(Page 9, line 279,** in red), discussed the differences between LAP1, LAP2 and LIP **(Page 20, line 448**, in red), and the limitations of this work **(Page 21, line 472**, in red).

It is also important to note that LIP overexpression in several other cell systems, including a mouse hepatoma cell line, shows the opposite; that LIP induces a glycolytic flux and increases cell proliferation and cell survival. DOI: 10.1038/s42003-019-0461-z; DOI: 10.1016/j.molmet.2023.101726
Moreover, genetic depletion of LIP in mice results in a shift towards whole-body enhanced fat metabolism and reduced carbohydrate metabolism (DOI: 10.15252/embr.201439837; DOI: 10.3389/fragi.2021.738512).
Another study has shown that LIP is required to overcome repression of E2F targets by long C/EBPβ (LAP2) and C/EBPα (p42) proteins, and that LIP deficiency delays the entry into the cell cycle of regenerating hepatocytes (DOI: 10.1101/gad.557910).
Furthermore, LIP deficient mice display a reduced and delayed cancer incidence, and LIP overexpressing mice have an increased cancer incidence (including liver) (DOI: 10.7554/eLife.34985; DOI 10.1007/s00109-014-1215-5).

Altogether the conclusion from most literature is that LIP is the oncogene and LAP2 has tumor suppression functions.

We appreciate the reviewer's valuable insights regarding the functions of LIP and its relationship to other C/EBPβ isoforms, particularly in the context of glycolysis and tumorigenesis. We acknowledge that previous studies have demonstrated oncogenic functions of LIP, which were not sufficiently discussed in the original manuscript. Therefore, in the revised version, we have expanded our discussion to explore the oncogenic roles of different C/EBPβ isoforms and their relevance to our findings.

Both LAPs and LIP exhibit oncogenic functions, as reported in various cell types and conditions. For instance, Liu et al[13] reported that C/EBPβ-dependent transcriptional networks mediate obesity-driven tumor initiation. This study[13] identified LAP2, but not LIP, showed oncogenic properties by promoting tumor sphere formation and upregulating *CLDN1* and *LCN2* in both MDA-MB-231 and HCC1806 cells (As shown in referee Fig. 9 left). In two TNBC mouse models, glycolysis restriction was shown to repress LAP2 expression specifically[14]. By overexpressing or deplete (mutant AUG) different C/EBPβ isoforms, they found that LAPs, particularly LAP2, regulate *G-CSF* and *GM-CSF* expression to induce tumor immunosuppression through the development of myeloid-derived suppressor cells (MDSCs) [14] (As shown in referee Fig. 9 right). This work also highlights the connection between LAP isoforms and glycometabolism. Additionally, another study in TNBC[15] utilized integrative sequencing analyses to demonstrate that LAP enhances chromatin accessibility and activates the EGFR signaling pathway, which critically promotes TNBC growth, whereas LIP has minimal effects on these phenotypes. Importantly, It has shown by different groups that complete depletion of C/EBPβ increases the expression of the well-known tumor suppressor p53 in mice, and all three isoforms interact to suppress p53 transcriptional activity[16,17]. LAP1 has also been shown to enhance the transcription of *NQO1* and *GSTP1*, facilitating glioblastoma proliferation[18]. Moreover, C/EBPβ has been implicated in driving defective autophagy and NK cell dysfunction in solid tumors, where its inhibition restores NK cell functions against tumors[19]. Phosphorylation of C/EBPβ on Thr217 has been found to enhance its association with procaspases 1 and 8, thereby inhibiting their activation and promoting the survival of hepatic stellate cells[20].

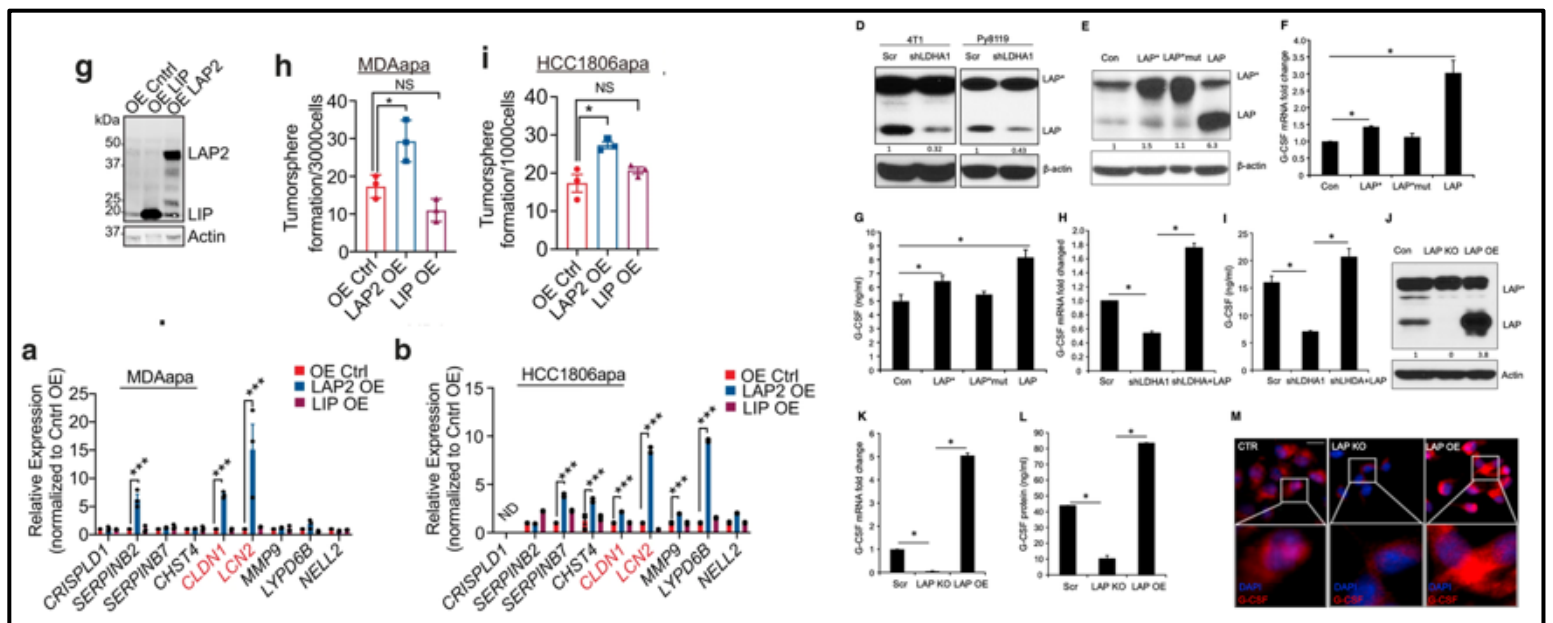

Referee Fig. 10 LAPs exhibit oncogenic functions. left, LAP2, but not LIP, showed oncogenic properties by promoting tumor sphere formation and upregulating CLDN1 and LCN2 in both MDA-MB-231 and HCC1806 cells[13] . **right,** LAPs, particularly LAP2, regulate G-CSF and GM-CSF expression to induce tumor immunosuppression through the development of myeloid-derived suppressor cells (MDSCs). Figures are from references 13 and 14.

There are also studies showing the oncogenic function of LIP specifically, just as you indicated. In mouse embryonic fibroblasts (MEF), LIP overexpression has been reported to promote aerobic glycolysis and mitochondrial respiration by activating the RNA-binding protein LIN28B[21,22]. Genetic depletion of LIP in mice has been associated with a metabolic shift towards enhanced fat metabolism and improved insulin sensitivity, while reducing carbohydrate metabolism[9,23]. These mice also experience delayed hepatocyte regeneration and impaired osteoclast differentiation[24]. Furthermore, LIP overexpression in mice has been linked to increased cancer susceptibility, particularly for T-lymphoma, and carcinomas of the skin, liver, and mammary gland[25]. However, how glucose consumption varies across cell types and cancer stages requires careful interpretation of the physiological functions of LIP, particularly since many of these experiments were not conducted in well-established liver cancer models, failing to reflect tumorigenesis in hepatocytes under hyperglycemic conditions.

In our study, we identified a correlation between LAP1 expression and glycometabolism (Fig. 1 in the manuscript). We then found that LAP1 enhances glycolysis and promotes cancer cell proliferation in HCC models. However, overexpression of LIP alone did not yield comparable effects in HepG2 cells or the xenograft model. Our primary focus was to elucidate which isoform establishes the link between hyperglycemia and HCC progression, and thus studying LIP's oncogenic functions was not the main emphasis of this work. We acknowledge that LIP, whether alone or in cooperation with LAP isoforms, may possess oncogenic functions under different contexts.

One crucial rationale for pursuing this project was our lab early observations that various tumor cell lines (including insulinoma and glioblastoma cells) expressing full-length C/EBPβ (LAP1) displayed accelerated growth and a "yellower" medium compared to control cells or those expressing LIP. This curiosity drove us to investigate the role of C/EBPβ in glycometabolism and cellular proliferation. Our sequencing, *in vitro*, and *in vivo* findings align well with these early observations.

We greatly appreciate the reviewer's exceptionally thorough understanding of the functions of LAP1, LAP2, and LIP. In the revised manuscript, we have addressed the potential differences between these isoforms

in the context of tumorigenesis (Page 15, line 318-353), which we believe enhances the relevance and significance of our work in the field of C/EBPβ oncology.

Page 16, Extended Data Figure 4H-K
The authors rush over the regulation of the HRAS promoter by C/EBPβ-LAP1.
Concerning the reporter assays they do not provide information about the part of the promoters used. This should be included. In addition, since they show the regulation, it must be possible to identify the positions of the binding sites using the consensus binding sequence (A/G T T G C G C/T A A A/G). By mutation of those binding sites the authors can provide proof that the regulation is through direct binding of LAP1.

We appreciate the reviewer's insightful feedback regarding the regulation of the HRAS promoter by C/EBPβ-LAP1. We have included detailed information about the promoter (region -2000 to +100 bp, related to TSS) used in the methods section of the revised manuscript (**Page 28, line 627**), and figure legends.

REDACTED

Furthermore, we conducted promoter prediction using JASPAR, which curates non-redundant transcription factor (TF) binding profiles stored as position frequency matrices (PFMs). Based on the predictions and the C/EBPβ consensus binding sequence, we identified potential C/EBPβ binding sites on the human HRAS promoter and mouse Hras promoter **(referee Fig 11**, or Extended data Fig 7e-g**).** We have validated one of these sites as the exact binding location of LAP1 through ChIP assays and luciferase assays that incorporated binding site mutations (**referee Fig 11** or Extended data Fig 7e-g). Our investigation of HRAS was motivated by our observation that LAPs enhance HepG2 cell proliferation (Fig 3 d,e in manuscript). Among the oncogenes that we screened, HRAS was the only one upregulated by LAPs (**Referee Fig 9 g,h** or Fig 4 e,f), and it is also known to be upregulated in diabetic patients[26-29].

The MotifMap (https://motifmap.ics.uci.edu) does not list C/EBP binding sites for HRAS

Thank you for bringing to our attention the MotifMap database! We have carefully checked the database, and confirmed that it does not list C/EBP binding sites for HRAS. However, we would like to point out that this database serves primarily as a resource for predicting potential binding sites. Many well-established downstream targets of C/EBPβ (e.g., *IL6, C3*[30]*, CLDN1, LCN2*[13]*, G-CSF, GM-CSF*[14]*, LGMN*[31]) are also not included in this database, and several genes listed have not been validated as targets of C/EBPβ. Despite these limitations, MotifMap does highlight a number of important genes that may be regulated by C/EBPβ binding, making it a worthwhile resource for our research and for future studies by others in this field.

Are there data available in ChIP-Seq databases that support the observation that HRAS contains C/EBPβ binding sites? I did not find any.

We did find one ChIP-Seq dataset performed in mouse dendritic cells[32], where C/EBPβ acts as a fate-determining TF for development, differentiation, proliferation, and activation. In their data, we observed a strong C/EBPβ binding peak in the approximate promoter region of *Hras*, supporting our findings (**Referee Fig 12 left**). In additon, our RNA-seq analysis revealed that C/EBPβ-KO HepG2 cells exhibit transcriptional repression of HRAS (**Referee Fig 12 left**, or Extended Data Fig. 8a). However, it is important to note that the same transcription factor (TF) may bind to different sites or exhibit different preferred DNA sequences across various cell types and under various conditions or stimuli[33,34]. To our knowledge, we did not find any datasets that directly align with our study on hyperglycemia-induced HCC. We appreciate your reminding regarding the importance of these approaches, and we plan to conduct our own ChIP-Seq experiments to examine C/EBPβ regulation in our future studies.

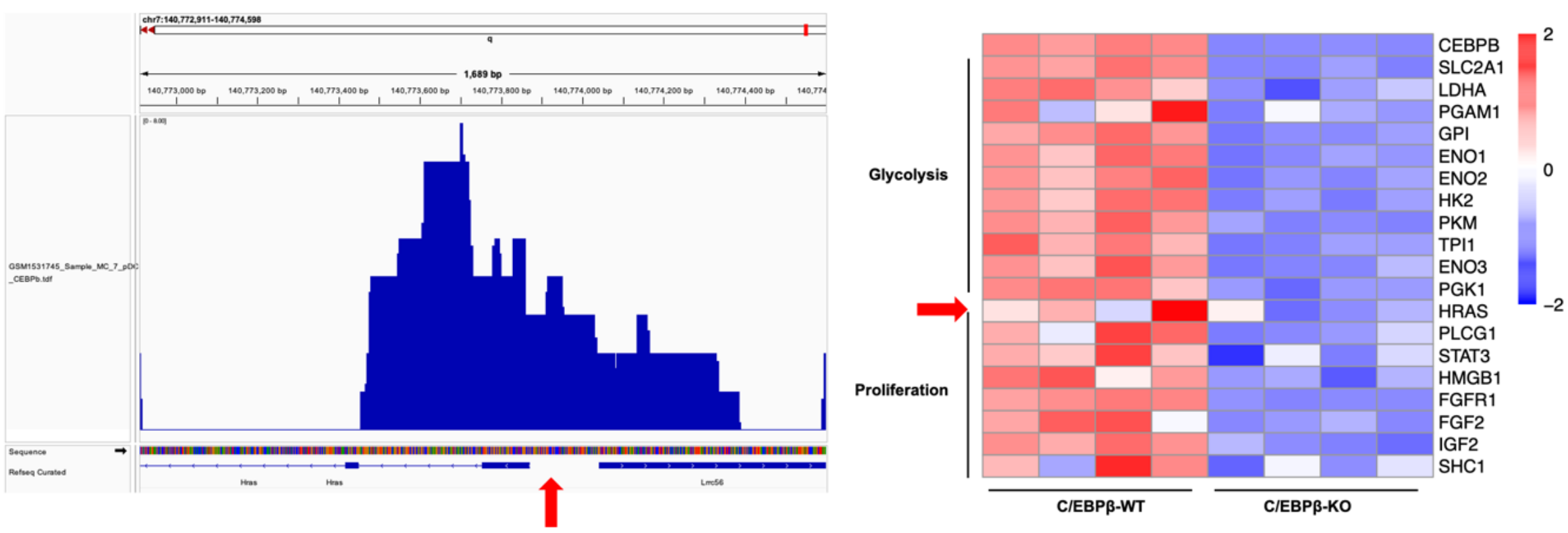

Referee Fig. 12. C/EBPβ regulates HRAS. Left, ChIP analysis showing binding of C/EBPβ to the mouse Hras promoter in monocyte-derived cells. Data were taken from a study by Bornstein et al.[34]. **Right,** (related to Extended Fig 8b) RNA-seq analysis revealed that C/EBPβ-KO HepG2 cells exhibit transcriptional repression of glycolytic effectors (e.g., GLUT1 (SLC2A1), LDHA) and proliferative drivers (e.g., HRAS).

Page 16, Figure 5
For panel H, in addition to LAP1/2 LIP should be shown.
Also here is the question whether LAP2 would give the same results. Without the experiments with LAP2 the data are difficult to interpret.

We have included LIP in Figure 5H as requested. Additionally, we conducted further experiments with LAP2 for Figure 5A-E. Our results indicate that either LAP1 or LAP2 restoration can rescue the inhibition of glycolysis and proliferation caused by C/EBPβ deletion (see **Referee Fig 13**, or Fig. 5 in manuscipt).

REDACTED

Page 19, Figure 6
If the used Rosa26-LSL-LAP allele expresses LAP1(?), the experiments show that LAP1 stimulates HCC in vivo. But again, what about LAP2?
In addition, ideally one would also like to know the effect of LIP expression, since LIP overexpression in another mouse model increases cancer incidence and blunted LIP expression in another mouse model reduces cancer incidence.

The Rosa26-LSL-LAP allele, which we now refer to as Rosa26-LSL-LAP1, expresses the full-length form of C/EBPβ (LAP1). We have added this clarification to the manuscript (**Page 13, line 272**) and methods section (**Page 23, line 501**).

Given that our primary focus is on glycometabolism in oncogenesis and the regulation observed through LAPs, we did not explore the other functions of LIP in relation to oncogenesis. Constructing a new conditional-overexpressing model for LAP2/LIP would require an additional 1-2 years, and therefore, we must restrict our conclusions to LAP1 at this time. We have acknowledged this limitation in the manuscript (**Page 21 Line 472**). We agree that studying LAP2 and LIP is important, and we plan to establish new models to address these questions in future studies. Thank you for your valuable suggestions!

Page 22, Figure 7
Please also show LIP in blots of panels B and G, and the exposure should of the C/EBPβ blot B could be stronger.

We have now included LIP in the blots for panels B and G of Figure 7, and we have made adjustments to the exposure of the C/EBPβ blot in panel B to provide clearer visibility (as shown in **Referee Fig. 14**).

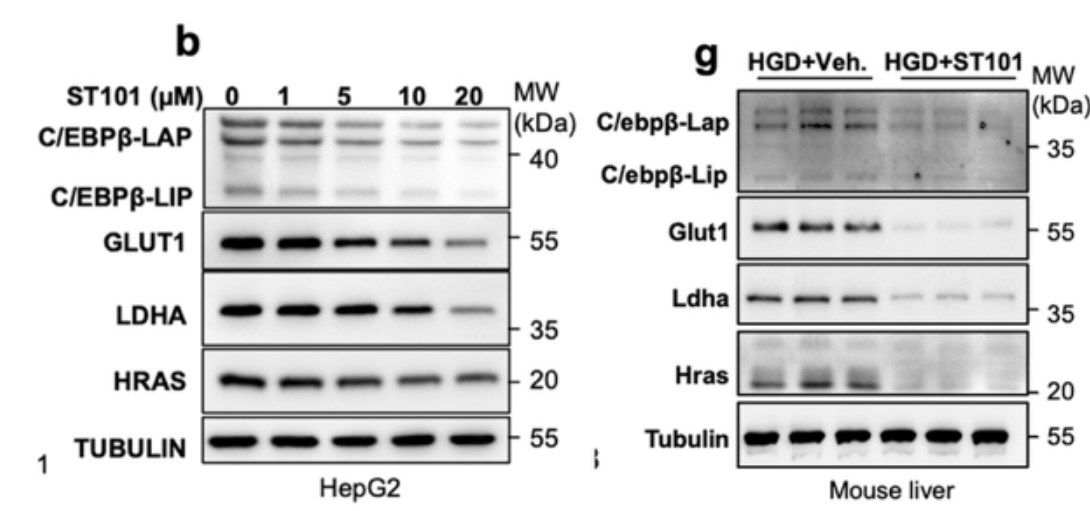

Referee Fig. 14. (Fig 7 in manuscript).

The authors mention the mouse model is hyperglycemia-induced HCC progression. However, how was HCC induced? Are these LAP1-LTP mice? HCC induced with DEN/CC14?

It is DEN/CCl4 model, just as we discribed in results (Page 11, Line 245) and methods sections (Page 25, line 554), and illustrated in **Fig. 5g and Fig. 6b.**
Result:
"To investigate the in vivo mechanism of hyperglycemia-driven hepatocellular carcinoma (HCC) progression, we employed a N-nitrosodiethylamine/carbon tetrachloride (DEN/CCl_4)-induced HCC mouse model that recapitulates key features of human HCC pathogenesis, including inflammation and fibrogenesis"
Methods:
"DEN/CCl_4 Hepatocellular Carcinoma Model Male mice received intraperitoneal N-nitrosodiethylamine (DEN; 1 mg/kg) at postnatal day 14, followed by twice-weekly carbon tetrachloride (CCl_4; 0.2 mL/kg i.p.). 100% of male mice developed hepatocellular carcinoma by 22 weeks."

Methods
Animals: What is exactly in the Rosa26-LSL-LAP allele? LAP1 or LAP2 sequences?

Thank you for your question regarding the Rosa26-LSL-LAP allele! We have now clarified that the Rosa26-LSL-LAP allele, which we now refer to as Rosa26-LSL-**LAP1**, expresses the full-length form of C/EBPβ (LAP1). We have added this detail to the description in the manuscript (**Page 13, line 272**) and methods section (**Page 23, line 501**).

Reviewer #2 (Remarks to the Author):

This study identifies the transcription factor C/EBPB as a missing mechanistic link between hyperglycemia and accelerated hepatocellular carcinoma (HCC) progression. By delineating the ROS-pERK-eIF2alpha-ATF4 signaling axis as the primary driver of C/EBPB upregulation, the authors provide a molecular explanation for the poor prognosis observed in diabetic cancer patients. They have a good amount of mouse data and patient data to help drive the importance of studying this pathway. Furthermore, the successful suppression of tumor growth using the clinical-phase peptide ST101 validates it as a high-value therapeutic target, offering a precision medicine approach to treat glucose-fueled malignancies.

We sincerely thank you for your positive comments regarding our study. Your acknowledgment of the significance of our findings is greatly appreciated and means a lot to us!

•The in vitro data indicates an increase in Glut1, but the patient data in figure 1a indicates no difference in diabetic vs non. How do the authors reconcile these results?

Thank you for bringing this to our attention! In our previous manuscript, while we observed an increase in GLUT1 expression, the results did not reach statistical significance due to the limited sample size and the heterogeneity of HCC cases. Following the advice from several reviewers, we expanded our sample size through collaboration with Xinhua Hospital affiliated with Shanghai Jiao Tong University and The First Affiliated Hospital of Guangxi Medical University. As a result, the number of patients in each group increased from 7 to 17 (patient information is updated in the Supplementary Table 1-3, also at the end of this response letter).

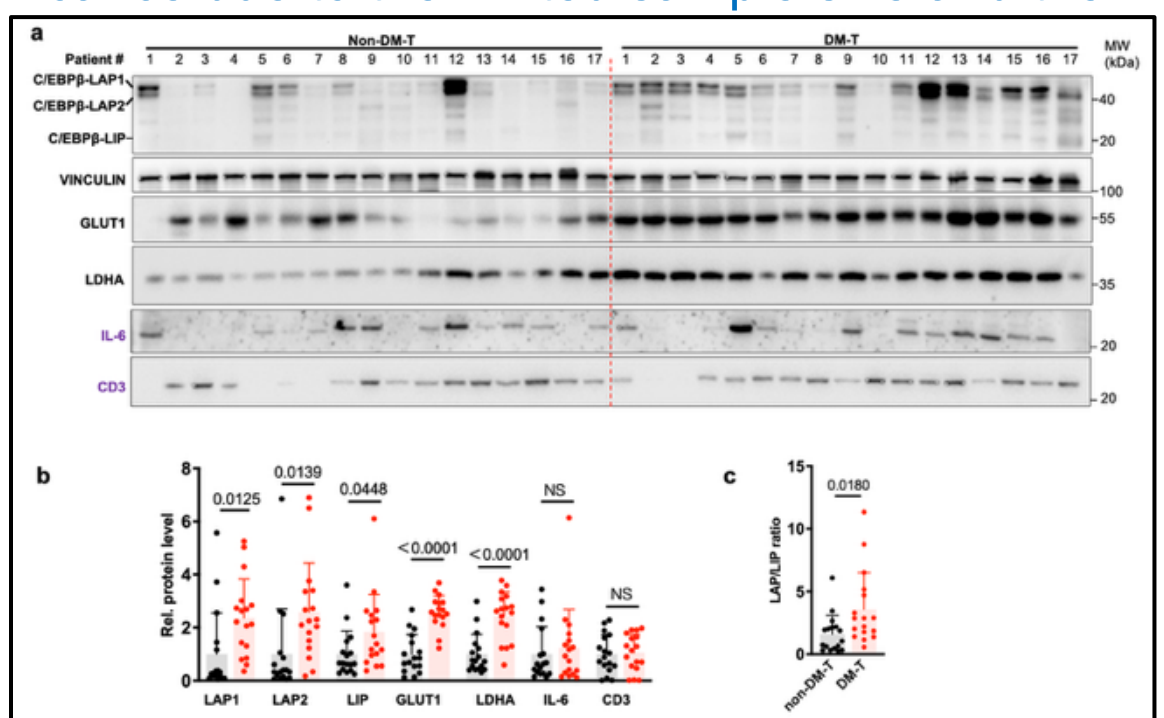

Referee Fig. 14 (Fig 1). **Increased hepatic C/EBPβ expression in HCC patients with DM.** **a, b,** Immunoblotting **(a)** and Quantification **(b)** in tumor (T) from Hepatocellular Carcinoma (HCC) patients with or without Diabetes mellitus (DM). **c,** The LAP/LIP ratio in tumor from HCC patients with or without DM. LAP/LIP ratio was calculated by adding LAP1 and LAP2 and dividing this value by LIP.

By analyzing these combined data from both the new and original samples, we found that GLUT1 expression is significantly increased in diabetic patients compared to non-diabetic patients (As shown in **Referee Fig 14**, or Fig 1a,b in manuscript), which aligns with the literature[32,35,36] and supports our *in vitro* and *in vivo* mouse model results.

•In many of their western blots, the authors switched between vinculin and tubulin as their housekeeping proteins, but it is not clear why.

Thank you for your observation regarding the use of vinculin and tubulin as housekeeping proteins in our Western blots! Both of these proteins are commonly utilized as loading controls in the field[37-42], because they are expected to maintain stable expression under our experimental conditions. We acknowledge the importance of consistency in selecting housekeeping proteins. The decision to use either vinculin or tubulin was made to accommodate variations across different experiments, enhance sample usage efficiency, and ensure accurate normalization of our data sets. We have added further clarification in the methods section (**Page 26, line 292**, in red) regarding this choice.

To enhance the rigor of our results, we will ensure that future experiments try to consistently use one housekeeping protein for comparison. This will help eliminate any potential confusion and provide clearer data interpretation.

•In Figure 1a, while I see their trend in the HCC tumor, in the HCC paracancerous tissue, it seems like it is just one patient that is driving the increase in C/EBPbeta expression more than in the tumor samples. As such, those blots do not appear to validate their claim that C/EBPbeta LAP increases in HCC paracancerous tissue. Also, it is not clear why they included the paracancerous tissue.

Thank you for your insightful comments regarding Figure 1a. In the previous version of the manuscript, we analyzed paracancerous and tumor tissues together. C/EBPβ expression was relatively enriched in the tumor group, leading to generally weak signals in the paracancerous group under the same exposure conditions. The original purpose of including paracancerous tissue was to investigate whether C/EBPβ is also upregulated in non-tumor tissue. Therefore, indeed, direct comparisons with tumor tissue were not necessary.

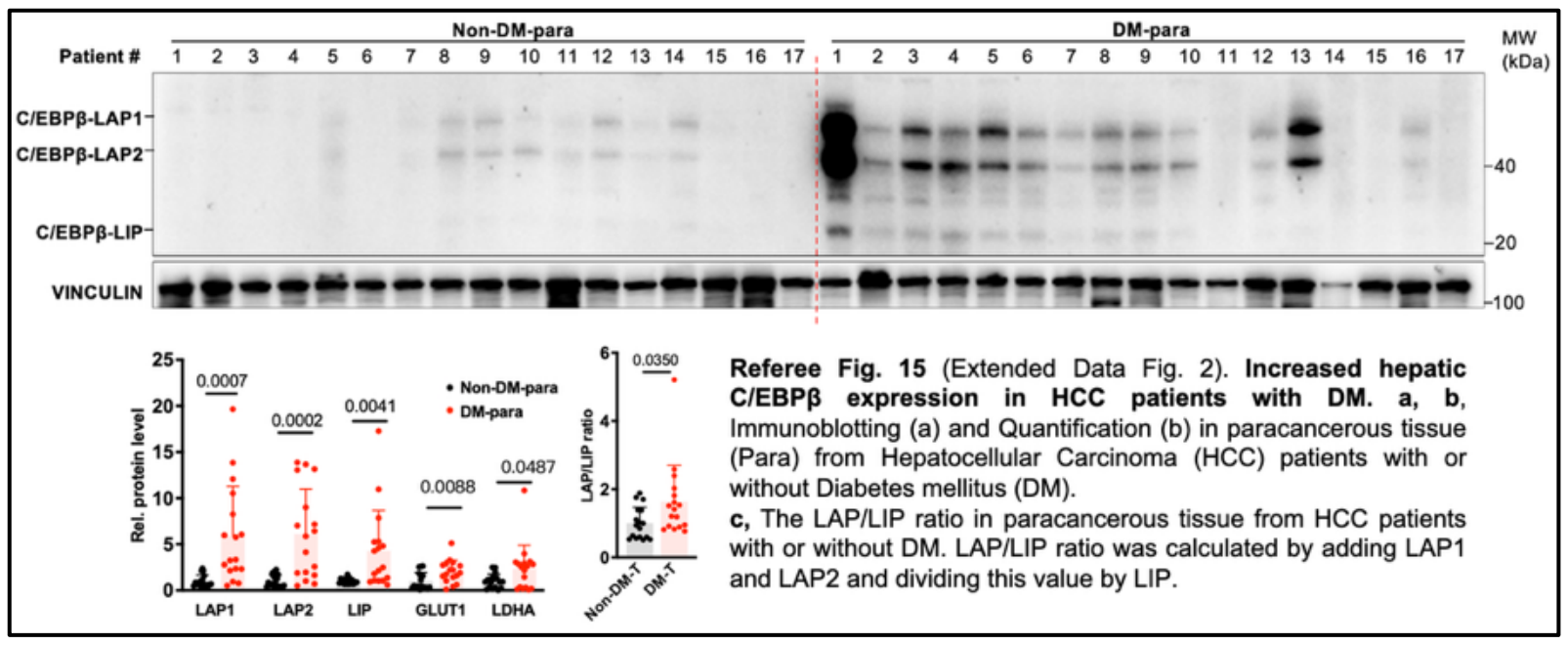

Referee Fig. 15 (Extended Data Fig. 2). **Increased hepatic C/EBPβ expression in HCC patients with DM. a, b,** Immunoblotting (a) and Quantification (b) in paracancerous tissue (Para) from Hepatocellular Carcinoma (HCC) patients with or without Diabetes mellitus (DM).
c, The LAP/LIP ratio in paracancerous tissue from HCC patients with or without DM. LAP/LIP ratio was calculated by adding LAP1 and LAP2 and dividing this value by LIP.

In response to this, we have reanalyzed the paracancerous tissues separately using a longer exposure time. The revised results demonstrate that C/EBPβ expression is clear, and significantly increased in the diabetic (DM) paracancerous group compared to the non-diabetic (non-DM) paracancerous control group (see **Referee Fig 15**, or Extended Fig 2). We have updated the relevant text and figure presentation in the revised manuscript accordingly.

•The authors only use the high-glucose diet mouse model for their model of hyperglycemia (normally regarded as a type 2 diabetes model). In their intro they indicate cancer mortality is not affected by the type of diabetes. Can they validate their results in a type 1 model to make sure that difference is still there between C/EBPbeta protein expression, the glycolytic enzyme expression, and those downstream ROS-PERK-eIF2α-ATF4 axis proteins they investigate with their high glucose diet model.

REDACTED

Thank you for your valuable comments! This is a very good suggestion. We acknowledge that our original study primarily utilized a high-glucose diet mouse model, which is typically regarded as a type 2 diabetes model. To address your concern, we have now incorporated a well-established type 1 diabetes model using STZ-treated mice. Our findings demonstrate that C/EBPβ, GLUT1, and LDHA expression are upregulated in the livers of these diabetic mice, regardless of diabetes type (**Referee Fig 15 right**, or Extended Fig 1e). Furthermore, we observed an increase in BIP (key marker of ER stress) and ATF4 in the livers of these diabetic models, indicating the activation of the BIP-PERK-eIF2α-ATF4 signaling axis, which is found to be required for high glucose-induced C/EBPβ expression in our work (**Referee Fig 15)**. We also quantified the Western blot results to provide more robust statistical support for our findings.

These results enhance the validity of our original findings and confirm that the observed differences in protein expression are consistent across both diabetes models.

•The authors only use the patient data to investigate the increased expression of C/EBPbeta LAP and the glycolytic-linked proteins, but they should also test the patient data to determine if the downstream proteins

they measure in Figure 2 are also elevated in the patient samples to strengthen their in vitro/in vivo comparison.

In response to your comment, we have analyzed the patient samples to assess the expression levels of BIP and ATF4, which are downstream proteins in the signaling axis we investigated.

Our findings indicate that both BIP and ATF4 are significantly upregulated in diabetic HCC samples compared to non-diabetic controls (see **Referee Fig 16**, or Extended Fig 1). This analysis strengthens the overall conclusions of our study regarding the impact of diabetes on C/EBPβ signaling and its downstream effects.

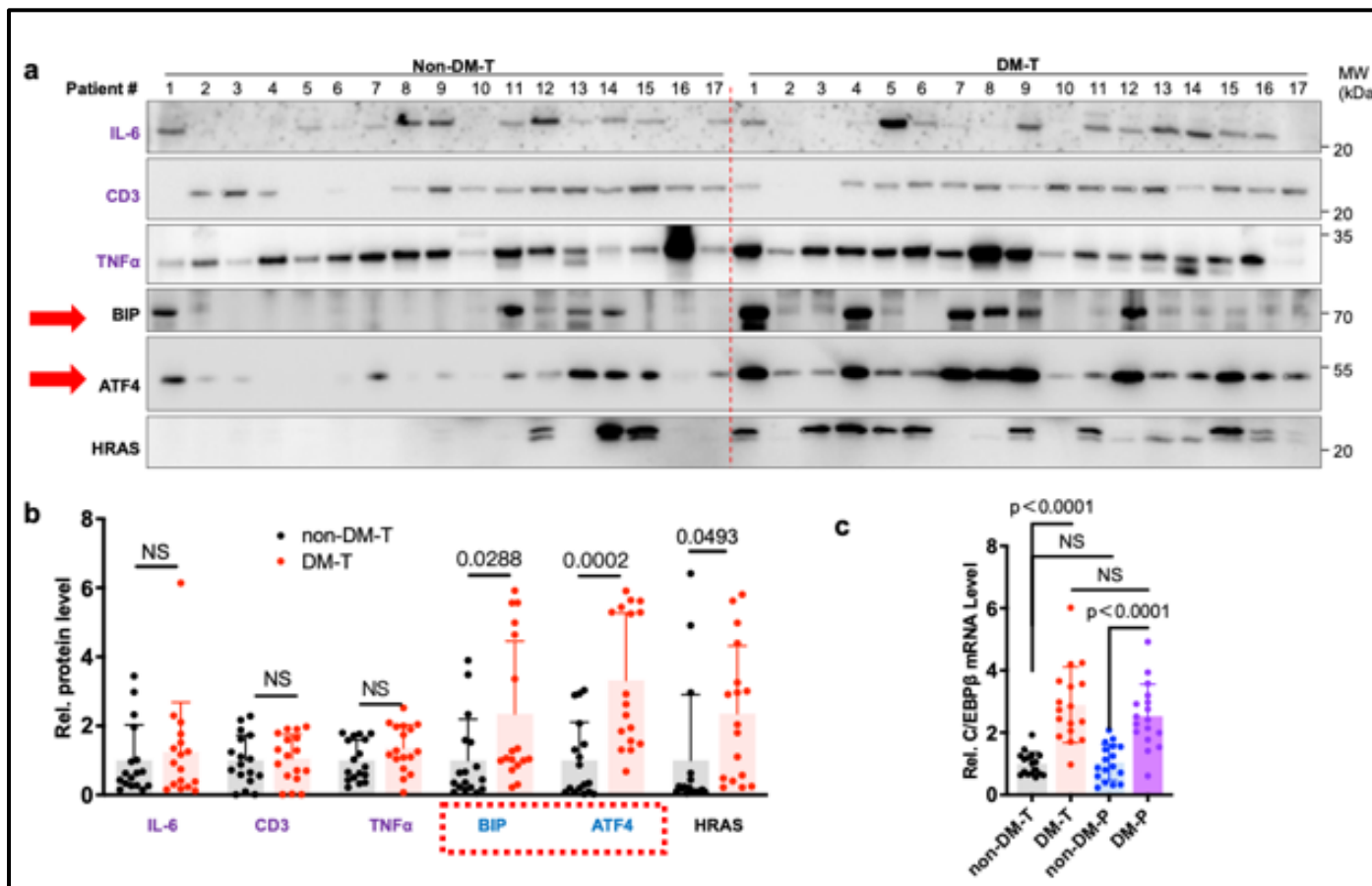

Referee Fig. 16 (Extended Data Fig 1) **a**, **b**, (related to Fig. 1a) Immunoblotting **(a)** and Quantification **(b)** in tumor (T) from Hepatocellular Carcinoma (HCC) patients with or without Diabetes mellitus (DM).

•Convention issue: the authors use n instead of N when they are referring to biological replicates

We have corrected all instances of "n" to "N" where appropriate to indicate biological replicates (highlighted in red in manuscript). Thank you for your attention to the details, and we appreciate your feedback in improving the quality of our manuscript!

•It is not a metastasis paper (as it mostly focuses on the primary tumor), but if the authors are seeing a difference in metastasis between their HGD and NCD conditions, or at least differences in invasion potential or migratory phenotype instead of just growth and proliferation, and the opposite trend when targeting their protein of interest, would also make their story more compelling.

Thank you for your valuable feedback regarding the focus on metastasis in our study! We agree that monitoring metastasis could enhance the compelling nature of our work.

In our DEN/CCL4-induced HCC mouse model, we did investigate potential metastasis to organs such as the lung and kidney. However, due to the limited developmental time and the inherent limitations of this model for studying metastatic behavior, we did not observe significant metastasis. Additionally, previous studies utilizing similar models did not reported any metastatic behavior[43-45]. As you noted, this is not a metastasis paper, and given the limitations of our current model, we have to not focus on metastasis.

We sincerely appreciate your constructive advice and recognize the importance of exploring differences in metastasis. We will certainly consider this aspect in our future studies, particularly by employing alternative tumor models (eg, MMTV-PyMT mice) that may be more suitable for investigating metastatic behavior.

Thank you once again for your insightful suggestions!

Reviewer #3 (Remarks to the Author):

This manuscript describes the mechanism by which hyperglycemia promotes the development of hepatocellular carcinoma (HCC). The authors present evidence linking hyperglycemia to the activation of the transcription factor C/EBPβ. They demonstrate that increased glucose levels lead to ROS-dependent activation of the PERK–eIF2α–ATF4 signaling pathway, resulting in enhanced transcription of C/EBPβ. Elevated levels of C/EBPβ subsequently promote glycolysis and cell proliferation in liver cancer through the upregulation of key glycolytic effectors and the oncoprotein HRAS. Furthermore, the authors illustrate that C/EBPβ overexpression drives HCC development, while genetic deletion or pharmacological inhibition of C/EBPβ reduces hyperglycemia-driven HCC progression.

Overall, the manuscript is well-structured and thoughtfully designed, with a clear and thorough experimental approach. The conclusions are strong for regulation, however the data do NOT support mediation. This needs to be corrected throughout the manuscript text, figure legends, the title, and the discussion section.

Thank you for your thorough review and constructive feedback regarding our manuscript! We appreciate your positive remarks about the structure and experimental approach of our study.

We agree your point regarding the terminology used throughout the manuscript. We have revised the text, figure legends, title, and discussion section to ensure clarity and accuracy. Please see the details below.

Major Concerns
1. The phrasing "by driving glycolysis and proliferation" implies a mechanistic causal pathway. The authors can only use this construction if the data directly demonstrate that glycolysis and proliferation mediate the phenotype downstream of C/EBPβ in a causality reversal experiment. The following statements need to be edited, or experiments need to be performed to prove causality. A few examples are noted below, but the authors are required to edit throughout.
Title: C/EBPβ Modulates Hyperglycemia-elicited Tumor Progression by Promoting Glycolysis
3 and Cell Proliferation - replace BY with AND

Extended Data Fig 6. C/EBPb-LAP fuels HCC development AND (not by) upregulating glycolytic genes and RAS.

Thank you for your valuable feedback regarding the terminology used in our manuscript! We agree that the phrasing incorrectly implies a mechanistic causal pathway.

In response to this, we have revised the title to: "C/EBPβ Modulates Hyperglycemia-Elicited Glycolysis, Cell Proliferation, and Tumor Progression." Additionally, we have updated the figure titles for Figure 6 and Extended Data Figure 6 to: "C/EBPβ-LAP1 upregulates glycolytic genes, Hras, and fuels HCC development." We have carefully reviewed the manuscript and made the necessary revisions throughout the text accordingly. Thank you again for your constructive input, which has helped us enhance the clarity and accuracy of our manuscript.

2. C/EBPβ is known to regulate pro-inflammatory molecules and influence immune responses, which are relevant in oncogenesis. It would be informative to evaluate downstream inflammatory effects, such as cytokine expression (IL-6, TNFα) or markers of immune cell infiltration (e.g., F4/80 or CD68 in mice, CD3/CD8 in human samples). This would clarify whether hyperglycemia-driven C/EBPβ activity promotes an inflammatory microenvironment that supports HCC development.

Thank you for your insightful suggestions regarding the role of C/EBPβ in regulating pro-inflammatory molecules and its impact on immune responses in the context of oncogenesis. We agree that understanding the inflammatory microenvironment is crucial for elucidating the mechanisms of hyperglycemia-driven HCC development.

In response to your comment, we examined the expression of the inflammatory cytokine IL-6, TNFa and the extent of immune T cell infiltration (CD3) in human HCC samples. Our findings indicate that while IL-6 expression is slightly elevated in diabetic HCC (DM-HCC) compared to non-diabetic HCC, neither IL-6, TNFa nor CD3 showed statistically significant differences (**Referee Fig 17 a-b**, or Extended Fig 1). Furthermore, we investigated the expression of IL-6, TNFα, and CD68 in mouse liver samples following specific deletion or overexpression of C/EBPβ (C/ebpβ^{WT}, C/ebpβ^{LKO}, C/ebpβ^{LTG}), and again found no significant changes (**Referee Fig 17** d-e, Fig 5g and 6c in manuscript).

REDACTED

Based on these results, we conclude that hyperglycemia-driven C/EBPβ activity does not promote HCC development through the induction of an inflammatory microenvironment. We have incorporated these data into the manuscript and modified the relevant descriptions accordingly (**Page 6 Line 116; Page 12, Line 252**).

3. The authors convincingly demonstrate that C/EBPβ upregulates HRAS across multiple models and that its knockdown attenuates HCC. It would strengthen the manuscript to examine whether other oncogenes, such as c-MYC or CCND1, are also regulated by C/EBPβ. Since p53 is known to be inhibited by C/EBPβ, it would be interesting to look at p53 levels in the patient samples.

Thank you for your positive feedback on our work and for your insightful suggestions! We also share your curiosity about the potential regulation of additional oncogenes by C/EBPβ.

To address this, we examined the expression levels of c-MYC, CCND1, NRAS, and KRAS in HepG2 cells following knockdown of C/EBPβ and overexpression of its isoforms (**Referee Fig 18**, or Fig 4 e-f in manuscript). Our results indicate that the expression levels of these oncogenes did not change significantly, confirming that only HRAS is the key downstream effector of C/EBPβ in the context of HCC progression.

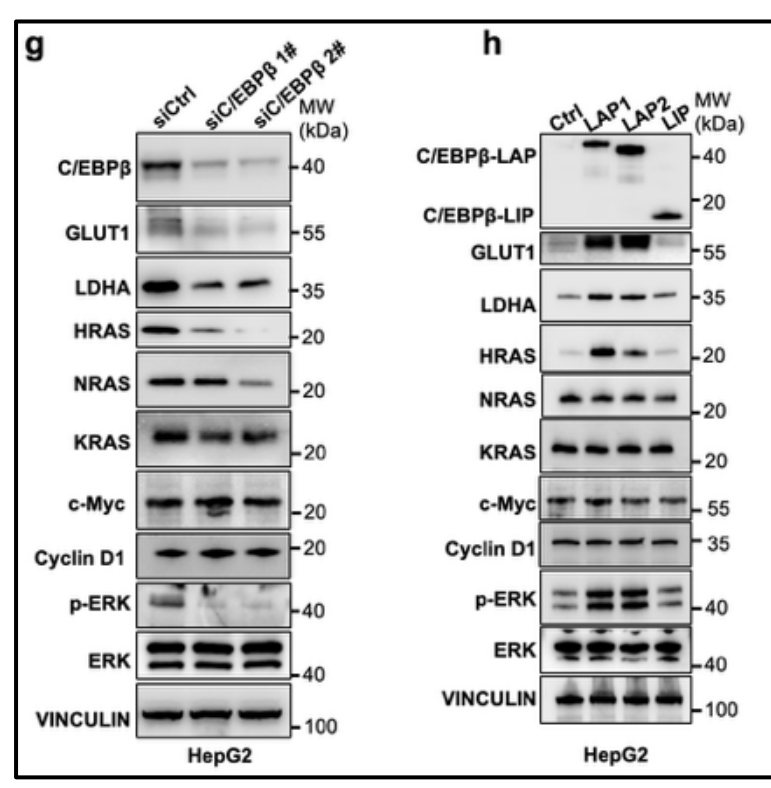

Referee Fig. 18 related to Fig 4, e-f C/EBPβ-LAP1 and LAP2 upregulate the expression of key glycolytic genes and HRAS in liver cancer cells.

Regarding P53, We investigated P53 expression in human hepatocellular carcinoma (HCC) patient samples and found that P53 levels decreased in the diabetic (DM) group compared to non-diabetic controls (**Referee Fig 20**); however, this difference did not reach statistical significance.

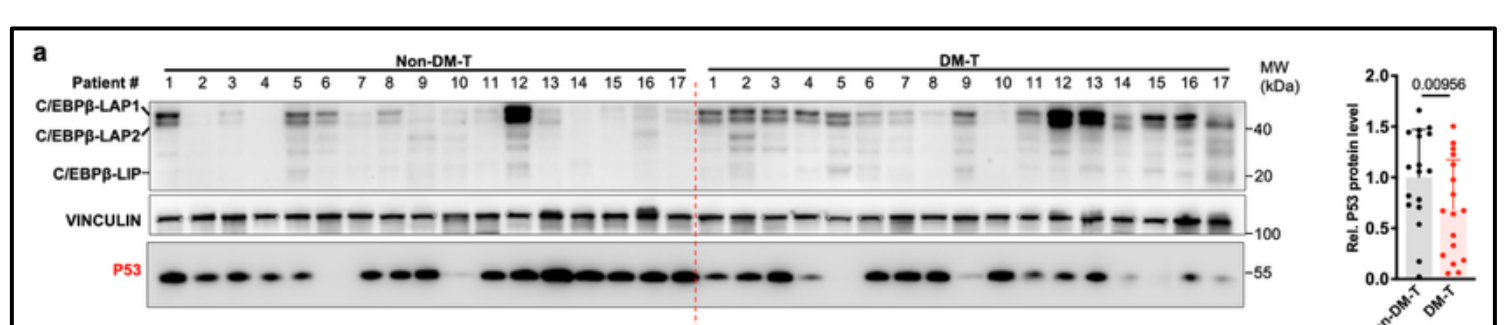

Referee Fig. 20 P53 protein level in human HCC tumor samples

While it is documented that C/EBPβ interacts to suppress p53 transcriptional activity[16,17], the regulation of p53 is extremely complex. Its levels are influenced by a variety of factors, including protein stability (such

as ubiquitination), protein activity, and subcellular distribution[46]. Given that p53 is known as "the guardian of the genome" and is mutated in approximately 50% of cancer cases, it plays a vital role as a tumor suppressor. In contrast, mutant forms of p53 can exhibit oncogenic properties[47]. To fully understand the functions of p53 in human samples, comprehensive information on the p53 genotype is essential. Unfortunately, genotype data was not fully available for our patient samples. As our study is not primarily focused on p53 or tumor suppression, we chose not to include this data to avoid potential misleading interpretations.

We appreciate your understanding and will seek to obtain complete p53 information in future studies.

4. For the patient cohort in extended figure 1, are the cancer stages for the patients known? Does the "phase 1/phase 2" designation address this? If so, please clarify. Furthermore, is there information on whether the patients have diabetes alone or if they fall within the spectrum of non-alcoholic fatty liver disease (NAFLD)? This information could be particularly valuable given the interplay between metabolism, inflammation, and tumor development.

We apologize for the lack of clarity in the information provided. We have now updated the manuscript to include detailed information on cancer stages in **Supplementary Table 1-3** (also see in the end of this response letter). The "Phase 1/Phase 2" designation does correspond to the cancer stages of the patients, and we have clarified this in the revised text.

Additionally, we have gathered information on whether the patients have diabetes alone or if they fall within the spectrum of fatty liver disease, based on their ultrasonography and medical history. Specifically, Patient #13 from the non-DM group, and Patients #9 and #11 from the DM group were identified as also suffering from fatty liver disease. This information has been incorporated into the patient information tables. We note that classifying detailed NAFLD-related characteristics would typically require additional analyses, such as HE staining and Oil Red O staining. However, we did not collect samples for these analyses, and since this study is not focused on fatty acid metabolism, we are limited to providing the current information.

Thank you for your constructive feedback, which has been invaluable in enhancing the clarity of our manuscript.

5. In the TCGA and GTEx cohorts analyzed in extended data 2, it would be helpful to report whether C/EBPβ mRNA differs between tumor and non-tumor samples or across stages. Stratification by diabetes status (if possible) could further clarify the relevance of hyperglycemia. If the mRNA expression data do not closely correlate with the protein levels (like fig 1a and extended fig 1c), the potential for regulation at the post-translational level should be discussed.

Thank you for your valuable suggestions regarding the analysis of C/EBPβ mRNA expression in cancer databases! We show here a comparison of C/EBPβ mRNA levels between HCC tumor and non-tumor samples in the revised manuscript in cancer databases (**Referee Fig 19,** or Extended data Fig 4c). However, these databases do not provide information regarding diabetes status. Our analysis of HCC samples form DM/non-DM patients, indicates that hepatic C/EBPβ levels are indeed elevated in DM groups.

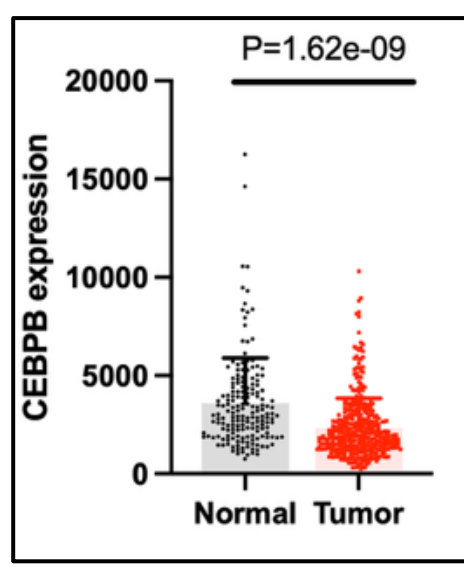

Referee Fig. 19 CEBPB mRNA expression in the liver samples from non-cancerous patients (N=225) and HCC patients (N=371). Data from TNMplot, which includes samples from GEO, GTEx, TCGA, and TARGET repositories,

It is noteworthy that we observed a lack of correlation between mRNA expression levels and protein levels in HCC patients. This discrepancy suggests that, during HCC oncogenesis, additional regulatory mechanisms, such as the ubiquitylation of C/EBPβ, may be involved. We discussed This discrepancy in the discussion section (Page 6 Line 129, and Page 19 Line 426, in red), and we plan to work on it in the future research. Thank you again for your thoughtful feedback, which has aided us in enhancing our manuscript's clarity and depth!

Minor Concerns

6. Some references require verification. For instance, the citations related to elevated fasting glucose and liver cancer risk (line 59) and the strong epidemiological link between HCC, colon adenocarcinoma, and hyperglycemia or diabetes mellitus (line 114) need to be clarified.

We have added references (line 63, line 118, in red).

7. The introduction/discussion section would benefit from a clearer overview of the current understanding of the roles of C/EBPβ and its isoforms, including LIP and the LAPs, in hepatocellular carcinoma. At present, it is difficult to place the findings in the context of existing literature. If the role of C/EBPβ in HCC remain poorly characterized, explicitly stating this would help emphasize the novelty and significance of the authors' observations.

We added a full paragraph discussing the current understanding of the roles of C/EBPβ and its isoforms, including LIP and the LAPs, in hepatocellular carcinoma and other oncology (**Page 15, Line 319 to 354,** in red).

8. In addition, it would be valuable to include a discussion of how ST101 is thought to act on C/EBPβ isoforms, particularly LIP and LAP1/2, and how this mechanism may be relevant in the context of hyperglycemia-driven HCC. A brief paragraph outlining the known or proposed effects of ST101 on C/EBPβ isoform balance, and how this could intersect with metabolic stress or hyperglycemia-induced signaling pathways, would strengthen the mechanistic interpretation of the therapeutic data presented in this study.

Thank you for pointing out this limitation! We have now addressed it in the Discussion (**Page 17, Line 371**, in red), highlighting how ST101 affects different isoforms and its impact on tumor treatment.

9. Edit to avoid overstatement of the results. "Thus, C/EBPβ is a critical regulator and therapeutic target for glucose-fueled cancer progression by driving glycolysis and proliferation". Please change to potential therapeutic target.

We agree that it is important to avoid overstating our findings. We have revised the statement as suggested (**Page 3, lin53; Page 21, Line 474**).

We appreciate your guidance on our work, which has helped improve the accuracy and clarity of our manuscript.

Reviewer #4 (Remarks to the Author):

I co-reviewed this manuscript with one of the reviewers who provided the listed reports. This is part of the Nature Communications initiative to facilitate training in peer review and to provide appropriate recognition for Early Career Researchers who co-review manuscripts.

Thank you for your valuable input.

Reference:

1 Kumar, A. *et al.* 2-Deoxyglucose drives plasticity via an adaptive ER stress-ATF4 pathway and elicits stroke recovery and Alzheimer's resilience. *Neuron* **111**, 2831-2846 e2810 (2023). https://doi.org/10.1016/j.neuron.2023.06.013

2 Scheuner, D. *et al.* Translational control is required for the unfolded protein response and in vivo glucose homeostasis. *Mol Cell* **7**, 1165-1176 (2001). https://doi.org/10.1016/s1097-2765(01)00265-9

3 Oyadomari, S., Harding, H. P., Zhang, Y., Oyadomari, M. & Ron, D. Dephosphorylation of translation initiation factor 2alpha enhances glucose tolerance and attenuates hepatosteatosis in mice. *Cell Metab* **7**, 520-532 (2008). https://doi.org/10.1016/j.cmet.2008.04.011

4 Eizirik, D. L., Cardozo, A. K. & Cnop, M. The role for endoplasmic reticulum stress in diabetes mellitus. *Endocr Rev* **29**, 42-61 (2008). https://doi.org/10.1210/er.2007-0015

5 Cnop, M., Toivonen, S., Igoillo-Esteve, M. & Salpea, P. Endoplasmic reticulum stress and eIF2alpha phosphorylation: The Achilles heel of pancreatic beta cells. *Mol Metab* **6**, 1024-1039 (2017). https://doi.org/10.1016/j.molmet.2017.06.001

6 Han, H. *et al.* Blood glucose concentration and risk of liver cancer: systematic review and meta-analysis of prospective studies. *Oncotarget* **8**, 50164-50173 (2017). https://doi.org/10.18632/oncotarget.16816

7 Zaini, M. A. *et al.* A screening strategy for the discovery of drugs that reduce C/EBPbeta-LIP translation with potential calorie restriction mimetic properties. *Sci Rep* **7**, 42603 (2017). https://doi.org/10.1038/srep42603

8 Calkhoven, C. F., Muller, C. & Leutz, A. Translational control of C/EBPalpha and C/EBPbeta isoform expression. *Genes Dev* **14**, 1920-1932 (2000).

9 Zidek, L. M. *et al.* Deficiency in mTORC1-controlled C/EBPbeta-mRNA translation improves metabolic health in mice. *Embo Rep* **16**, 1022-1036 (2015). https://doi.org/10.15252/embr.201439837

10 Kowenz-Leutz, E. & Leutz, A. A C/EBP beta isoform recruits the SWI/SNF complex to activate myeloid genes. *Mol Cell* **4**, 735-743 (1999). https://doi.org/10.1016/s1097-2765(00)80384-6

11 Liu, M., Du, Q., Mao, G., Dai, N. & Zhang, F. MYB proto-oncogene like 2 promotes hepatocellular carcinoma growth and glycolysis via binding to the Optic atrophy 3 promoter and activating its expression. *Bioengineered* **13**, 5344-5356 (2022). https://doi.org/10.1080/21655979.2021.2017630

12 Chang, Y. S. *et al.* Long Noncoding RNA NTT Context-Dependently Regulates MYB by Interacting With Activated Complex in Hepatocellular Carcinoma Cells. *Front Oncol* **11**, 592045 (2021). https://doi.org/10.3389/fonc.2021.592045

13 Liu, X. Z. *et al.* C/EBPB-dependent adaptation to palmitic acid promotes tumor formation in hormone receptor negative breast cancer. *Nat Commun* **13**, 69 (2022). https://doi.org/10.1038/s41467-021-27734-2

14 Li, W. *et al*. Aerobic Glycolysis Controls Myeloid-Derived Suppressor Cells and Tumor Immunity via a Specific CEBPB Isoform in Triple-Negative Breast Cancer. *Cell Metab* **28**, 87-103 e106 (2018). https://doi.org/10.1016/j.cmet.2018.04.022

15 Wang, Y. *et al*. Divergent Roles of C/EBPbeta Isoforms LAP and LIP in Shaping T Cell Dysfunction and Tumor Progression in Triple-Negative Breast Cancer. *FASEB J* **39**, e70984 (2025). https://doi.org/10.1096/fj.202501330R

16 Schneider-Merck, T. *et al*. Physical interaction and mutual transrepression between CCAAT/enhancer-binding protein β and the p53 tumor suppressor. *Journal of Biological Chemistry* **281**, 269-278 (2006). https://doi.org/10.1074/jbc.M503459200

17 Ewing, S. J., Zhu, S., Zhu, F., House, J. S. & Smart, R. C. C/EBPβ represses p53 to promote cell survival downstream of DNA damage independent of oncogenic Ras and p19. *Cell Death Differ* **15**, 1734-1744 (2008). https://doi.org/10.1038/cdd.2008.105

18 Lei, K. *et al*. C/EBPbeta mediates NQO1 and GSTP1 anti-oxidative reductases expression in glioblastoma, promoting brain tumor proliferation. *Redox Biol* **34**, 101578 (2020). https://doi.org/10.1016/j.redox.2020.101578

19 Portale, F. *et al*. C/EBPbeta-dependent autophagy inhibition hinders NK cell function in cancer. *Nat Commun* **15**, 10343 (2024). https://doi.org/10.1038/s41467-024-54355-2

20 Buck, M., Poli, V., Hunter, T. & Chojkier, M. C/EBPbeta phosphorylation by RSK creates a functional XEXD caspase inhibitory box critical for cell survival. *Mol Cell* **8**, 807-816 (2001). https://doi.org/10.1016/s1097-2765(01)00374-4

21 Ackermann, T. *et al*. C/EBPbeta-LIP induces cancer-type metabolic reprogramming by regulating the let-7/LIN28B circuit in mice. *Commun Biol* **2**, 208 (2019). https://doi.org/10.1038/s42003-019-0461-z

22 Ackermann, T. *et al*. C/EBPbeta-LIP mediated activation of the malate-aspartate shuttle sensitizes cells to glycolysis inhibition. *Mol Metab* **72**, 101726 (2023). https://doi.org/10.1016/j.molmet.2023.101726

23 Bitto, A., Tatom, N., Krivak, T., Grotz, P. & Kaeberlein, M. Evidence that C/EBP-beta LAP Increases Fat Metabolism and Protects Against Diet-Induced Obesity in Response to mTOR Inhibition. *Front Aging* **2**, 738512 (2021). https://doi.org/10.3389/fragi.2021.738512

24 Wethmar, K. *et al*. C/EBPbetaDeltauORF mice--a genetic model for uORF-mediated translational control in mammals. *Genes Dev* **24**, 15-20 (2010). https://doi.org/10.1101/gad.557910

25 Begay, V. *et al*. Deregulation of the endogenous C/EBPbeta LIP isoform predisposes to tumorigenesis. *J Mol Med (Berl)* **93**, 39-49 (2015). https://doi.org/10.1007/s00109-014-1215-5

26 Gao, C. *et al*. High glucose-upregulated PD-L1 expression through RAS signaling-driven downregulation of PTRH1 leads to suppression of T cell cytotoxic function in tumor environment. *J Transl Med* **21**, 461 (2023). https://doi.org/10.1186/s12967-023-04302-4

27 Peeters, K. *et al*. Fructose-1,6-bisphosphate couples glycolytic flux to activation of Ras. *Nat Commun* **8**, 922 (2017). https://doi.org/10.1038/s41467-017-01019-z

28 Kowluru, R. A., Kowluru, A., Chakrabarti, S. & Khan, Z. Potential contributory role of H-Ras, a small G-protein, in the development of retinopathy in diabetic rats. *Diabetes* **53**, 775-783 (2004). https://doi.org/10.2337/diabetes.53.3.775

29 Vairaktaris, E. *et al.* Diabetes enhances the expression of H-ras and suppresses the expression of EGFR leading to increased cell proliferation. *Histol Histopathol* **24**, 531-539 (2009). https://doi.org/10.14670/HH-24.531

30 Ndoja, A. *et al.* Ubiquitin Ligase COP1 Suppresses Neuroinflammation by Degrading c/EBPbeta in Microglia. *Cell* **182**, 1156-1169 e1112 (2020). https://doi.org/10.1016/j.cell.2020.07.011

31 Wang, Z. H. *et al.* C/EBPbeta regulates delta-secretase expression and mediates pathogenesis in mouse models of Alzheimer's disease. *Nat Commun* **9**, 1784 (2018). https://doi.org/10.1038/s41467-018-04120-z

32 Bornstein, C. *et al.* A negative feedback loop of transcription factors specifies alternative dendritic cell chromatin States. *Mol Cell* **56**, 749-762 (2014). https://doi.org/10.1016/j.molcel.2014.10.014

33 Srivastava, D. & Mahony, S. Sequence and chromatin determinants of transcription factor binding and the establishment of cell type-specific binding patterns. *Biochim Biophys Acta Gene Regul Mech* **1863**, 194443 (2020). https://doi.org/10.1016/j.bbagrm.2019.194443

34 Lee, B. K. *et al.* Cell-type specific and combinatorial usage of diverse transcription factors revealed by genome-wide binding studies in multiple human cells. *Genome Res* **22**, 9-24 (2012). https://doi.org/10.1101/gr.127597.111

35 Sokolovska, J. *et al.* Comparison of the effects of glibenclamide on metabolic parameters, GLUT1 expression, and liver injury in rats with severe and mild streptozotocin-induced diabetes mellitus. *Medicina (Kaunas)* **48**, 532-543 (2012).

36 Chen, X. *et al.* Intracellular galectin-3 is a lipopolysaccharide sensor that promotes glycolysis through mTORC1 activation. *Nat Commun* **13**, 7578 (2022). https://doi.org/10.1038/s41467-022-35334-x

37 Su, Y. *et al.* Glucose-induced CRL4(COP1)-p53 axis amplifies glycometabolism to drive tumorigenesis. *Mol Cell* **83**, 2316-2331 e2317 (2023). https://doi.org/10.1016/j.molcel.2023.06.010

38 Chen, T. *et al.* NSUN2 is a glucose sensor suppressing cGAS/STING to maintain tumorigenesis and immunotherapy resistance. *Cell Metab* **35**, 1782-1798 e1788 (2023). https://doi.org/10.1016/j.cmet.2023.07.009

39 Su, Y. *et al.* Glucose-induced CRL4COP1-p53 axis amplifies glycometabolism to drive tumorigenesis. *Molecular Cell* **83**, 2316-+ (2023). https://doi.org/10.1016/j.molcel.2023.06.010

40 Hay, N. Reprogramming glucose metabolism in cancer: can it be exploited for cancer therapy? *Nat Rev Cancer* **16**, 635-649 (2016). https://doi.org/10.1038/nrc.2016.77

41 Miao, W. *et al.* Glucose dissociates DDX21 dimers to regulate mRNA splicing and tissue differentiation. *Cell* **186**, 80-97 e26 (2023). https://doi.org/10.1016/j.cell.2022.12.004

42 Lopez-Pajares, V. *et al*. Glucose modulates IRF6 transcription factor dimerization to enable epidermal differentiation. *Cell Stem Cell* **32**, 795-810 e710 (2025). https://doi.org/10.1016/j.stem.2025.02.017
43 Uehara, T., Pogribny, I. P. & Rusyn, I. The DEN and CCl(4) -Induced Mouse Model of Fibrosis and Inflammation-Associated Hepatocellular Carcinoma. *Curr Protoc* **1**, e211 (2021). https://doi.org/10.1002/cpz1.211
44 Dapito, D. H. *et al*. Promotion of Hepatocellular Carcinoma by the Intestinal Microbiota and TLR4. *Cancer Cell* **21**, 504-516 (2012). https://doi.org/10.1016/j.ccr.2012.02.007
45 Zhang, Q. *et al*. Proteomic analysis of DEN and CCl

-induced hepatocellular carcinoma mouse model. *Sci Rep-Uk* **14** (2024). https://doi.org/ARTN 8013

10.1038/s41598-024-58587-6
46 Sullivan, K. D., Galbraith, M. D., Andrysik, Z. & Espinosa, J. M. Mechanisms of transcriptional regulation by p53. *Cell Death Differ* **25**, 133-143 (2018). https://doi.org/10.1038/cdd.2017.174
47 Yue, X. *et al*. Mutant p53 in Cancer: Accumulation, Gain-of-Function, and Therapy. *J Mol Biol* **429**, 1595-1606 (2017). https://doi.org/10.1016/j.jmb.2017.03.030

Supplementary Table 1.
Characteristics of LIHC patients, whose samples were collected for this study. Data are expressed as means ± SD. FPG, fasting plasma glucose.

characteristic	Non-diabetic patients	Diabetic patients	P value
**Gender (Male/Female)**	15/2	15/2	n.a.
**Age (years)**	61.5±10.71	62.9±10.1	0.669
**FPG (μmol/l)**	5.82±0.93	7.42±2.80	0.021
**BMI (kg/m^2)**	22.3±2.7	23.7±2.6	0.127
**HCC stage (Phase 1/2/3/4)**	3/13/1/0	3/13/0/1	n.a.

Supplementary Table 2.
Non-diabetic patient sample information

Patient #	DM/non-DM	Gender	Age (years)	FPG (μmol/l)	BMI (kg/m^2)	HCC stage	Fatty liver disease	# in the previous manuscript
1	non-DM	Male	50	6.98	22.48	1	No	#2
2	non-DM	Male	44	5.00	22.49	1	No	#1
3	non-DM	Male	67	5.23	19.23	1	No	#3
4	non-DM	Male	78	5.96	23.44	2	No	#4
5	non-DM	Male	51	5.92	24.17	2	No	#5
6	non-DM	Male	66	6.29	20.08	2	No	#6
7	non-DM	Male	56	6.19	27.55	2	No	#7
8	non-DM	Male	58	5.9	20.8	2	No	n.a.
9	non-DM	Male	50	4.55	19.2	2	No	n.a.
10	non-DM	Male	69	6.4	19.0	2	No	n.a.
11	non-DM	Male	47	5.67	21.8	2	No	n.a.
12	non-DM	Male	77	8.28	26.1	2	No	n.a.
13	non-DM	Female	65	5.99	23.0	2	Fatty liver disease	n.a.
14	non-DM	Male	59	4.27	19.1	3	No	n.a.
15	non-DM	Male	76	5.36	26.4	2	No	n.a.
16	non-DM	Male	68	5.91	23.1	2	No	n.a.
17	non-DM	Female	65	5.11	20.9	2	No	n.a.

Supplementary Table 3.
Diabetic patient sample information

Patient #	DM/non-DM	Gender	Age (years)	FPG (μmol/l)	BMI (kg/m^2)	HCC stage	Fatty liver disease	# in the previous manuscript
1	Type 2 DM	Male	55	8.54	29.7	2	No	n.a.

2	Type 2 DM	Male	71	14.65	27.1	2	No	n.a.
3	Type 2 DM	Male	47	6.9	21.8	2	No	n.a.
4	Type 2 DM	Female	86	8.33	21.4	2	No	n.a.
5	Type 2 DM	Male	69	5.9	20.8	2	No	n.a.
6	Type 2 DM	Male	67	4.9	27.7	2	No	n.a.
7	Type 2 DM	Male	51	6.47	24.3	2	No	n.a.
8	Type 2 DM	Male	61	5.49	25.1	2	No	n.a.
9	Type 2 DM	Male	61	6.9	23.9	4	Fatty liver disease	n.a.
10	Type 2 DM	Male	76	7.03	22.9	2	No	n.a.
11	Type 2 DM	Female	68	8.87	27.11	1	Fatty liver disease	#1
12	Type 2 DM	Male	69	14.57	22.96	2	No	#2
13	Type 2 DM	Male	51	10.37	26.57	1	No	#3
14	Type 2 DM	Male	62	10.17	21.08	1	No	#4
15	Type 2 DM	Male	56	8.02	26.57	2	No	#5
16	Type 2 DM	Male	60	9.15	25.30	2	No	#6
17	Type 2 DM	Male	54	6.71	24.91	2	No	#7

A point-by-point response to the reviewers' comments

Reviewer #1 (Remarks to the Author):

The authors have adequately addressed all of my concerns and suggestions. In particular, the increased sample size has substantially strengthened the statistical power of the analyses, and the statistical evaluation has been appropriately updated. Although C/EBPβ detection in non DM para samples remains relatively low, the increase in signal in DM para samples is now clearly discernible (as shown in referee Fig. 1).

With respect to the distinct functions of LAP1 versus LAP2, the authors provide sufficient evidence to support the conclusion that, in this experimental context, LAP1 can functionally substitute for both isoforms. I recognize that generating additional mouse models to further dissect isoform specific roles would go beyond a reasonable effort for the scope of this study.

Overall, this study, together with previous work, supports the view that the physiological and pathological functions of C/EBPβ isoforms are highly context dependent. While the mechanistic basis for this context specificity remains incompletely understood, it provides a strong rationale and motivation for further investigation.

ANSWER: Thank you for your thoughtful comments and positive feedback on our study. We sincerely appreciate your valuable input, which has greatly improved our manuscript. We also deeply appreciate your recognition of our efforts. We will continue advancing our work in this exciting field and look forward to further exploring the unknowns.

Reviewer #2 (Remarks to the Author):

Overall the authors have done a good job in responding to reviews and increasing the rigor of their results.

ANSWER: Thank you for your encouraging remarks regarding our revisions. We appreciate your acknowledgment of the improvements made to the rigor of our results in response to previous feedback.

Reviewer #3 (Remarks to the Author):

The authors have performed additional experiments and clarified/edited where requested.

ANSWER: We sincerely appreciate your recognition of our efforts to address the comments provided. The additional experiments conducted and the clarifications made enhanced the clarity and impact of our study.
